# Association of triglyceride-glucose index (TyG) and a body shape index (ABSI) with cognitive decline and dementia risk

**DOI:** 10.1371/journal.pone.0354418

**Published:** 2026-07-22

**Authors:** Shuangshuang Yang, Lili Cui, Jinxin Zhang, Ying Yang, Junhao Huo, Yuyan Ding, Jingni Zhang, Shulan He, Jiangping Li

**Affiliations:** 1 Department of Epidemiology and Health Statistics, School of Public Health, Ningxia Medical University, Yinchuan, Ningxia Hui Autonomous Region, China; 2 Department of Science and Education, Qinghai Provincial People’s Hospital, Xining, Qinghai, PR China; 3 Key Laboratory of Environmental Factors and Chronic Disease Control, Ningxia Medical University, Yinchuan, Ningxia Hui Autonomous Region, China; The First Affiliated Hospital of Soochow University, CHINA

## Abstract

**Background:**

Longitudinal evidence linking the body shape index (ABSI) to dementia and its subtypes remains scarce. Furthermore, the use of ABSI and TyG-ABSI as predictors of cognitive decline and dementia is infrequent. Therefore, this study aimed to examine the relationships between the triglyceride-glucose (TyG) index, ABSI, and TyG-ABSI indices and their predictive value for cognitive decline and dementia risk.

**Methods:**

This study included 370,744 participants from the UK Biobank. The TyG and ABSI indices were calculated using their respective formulas, while the TyG-ABSI index was derived by multiplying TyG and ABSI. Cognitive decline was defined as scoring in the bottom 25% on cognitive tests, while dementia was diagnosed from first-recorded incidents and algorithmically-defined outcomes. Logistic regressions were employed to assess the associations of TyG, ABSI and TyG-ABSI indices with cognitive decline; Cox regressions were used to analyze the associations of these indices with the risk of all-cause dementia (ACD) and its subtypes.

**Result:**

The TyG, ABSI, and TyG-ABSI indices were all significantly associated with cognitive decline. Additionally, compared with the lowest quartiles, the highest quartiles of TyG, ABSI and TyG-ABSI indices were associated with a significantly increased risk of ACD by 33% (HR = 1.33, 95% CI: 1.13–1.57), 79% (HR = 1.79, 95% CI: 1.65–1.94) and 67% (HR = 1.67, 95% CI: 1.54–1.82), respectively. These indices were also significantly associated with the risk of Alzheimer’s disease (AD) and vascular dementia (VD) (all P < 0.05).

**Conclusion:**

The TyG, ABSI, and TyG-ABSI indices are significantly and positively linked to the risk of cognitive decline and dementia.

## 1. Introduction

With the intensification of global population aging, the prevalence of age-related cognitive decline and dementia is projected to rise significantly worldwide [1,2]. Projections indicate that by 2050, the global number of dementia patients will reach 131.5 million, making it a major public health issue that severely threatens the quality of life in older adults [3]. Cognitive decline is a gradual reduction in cognitive function from a normal level, while dementia is its severe stage with common subtypes including Alzheimer’s disease (AD) and vascular dementia (VD) [4,5]. Currently, there is no effective cure for dementia; treatments can only slow its progression [6]. Therefore, there is an urgent need for early identification of risk factors for cognitive decline and dementia, which is critical for implementing preventive measures and timely interventions.

The triglyceride-glucose index (TyG), calculated from fasting triglycerides (TG) and fasting plasma glucose (FPG), is a simple and cost-effective surrogate marker for insulin resistance (IR) [7,8]. IR is a condition characterized by reduced insulin efficacy, and it is also closely associated with visceral obesity. Visceral obesity not only leads to the development of insulin resistance but also sustains this condition by further exacerbating systemic metabolic disturbances [9,10]. Furthermore, visceral obesity can be assessed using a novel metric called the Body Mass Index (ABSI), which is a new indicator calculated based on waist circumference (WC), body mass index (BMI), and height. This metric enables independent evaluation of visceral adipose tissue beyond BMI alone and demonstrates superior performance in assessing metabolic risk compared to traditional indicators such as BMI, WC, and waist-to-height ratio (WHtR) [11,12]. It is well-established that both IR and obesity are significant risk factors for a spectrum of chronic conditions, such as cardiovascular disease, type 2 diabetes, and cognitive decline [13,14]. For instance, in the field of cognitive health, multiple cross-sectional studies have demonstrated a positive correlation between higher ABSI levels and the risk of cognitive impairment or dementia [15]. However, longitudinal evidence on the association between ABSI and dementia incidence remains critically scarce. Beyond their individual effects, the combined impact of IR and visceral obesity is of urgent research priority. Although previous studies have demonstrated that TyG and ABSI (integrated into the TyG-ABSI index) exhibit a significant combined predictive effect for cardiovascular diseases and stroke [16,17]. Nevertheless, the relationship between the TyG-ABSI index and cognitive decline as well as dementia risk has not been sufficiently studied and remains unclear.

Therefore, this study aimed to investigate the relationships of TyG, ABSI, and TyG-ABSI indices with cognitive decline and dementia risk, and assess their predictive value. It seeks to provide novel tools for early clinical identification and targeted intervention.

## 2. Methods

### 2.1 Study design and population

The UK Biobank is a large prospective cohort study that recruited over 500,000 participants across 22 centers in the UK between 2006 and 2010 [18,19]. The end date of follow-up for this study is September 2024. Participants, all registered with the National Health Service (NHS), completed detailed questionnaires, underwent physical assessments. The UK Biobank study received ethical approve from the North West Multi-center Research Ethics Committee (MREC) (REC reference: 11/NW/0382), and written informed consents were obtained from all participants. In accordance with the access agreement of the UK Biobank, all data provided to researchers were de-identified. The authors did not obtain any directly or indirectly identifying information of participants during the analysis. Ethical approval was obtained (North West MREC, 21/NW/0157), and all participants provided informed consent under the Helsinki Declaration. The Strengthening the Reporting of Observational Studies in Epidemiology (STROBE) guidelines for cohort studies were strictly adhered to in this study, ensuring standardized, high-quality, and complete reporting. This study is conducted under application number 98124 for UK Biobank Resource.

**Fig 1:**
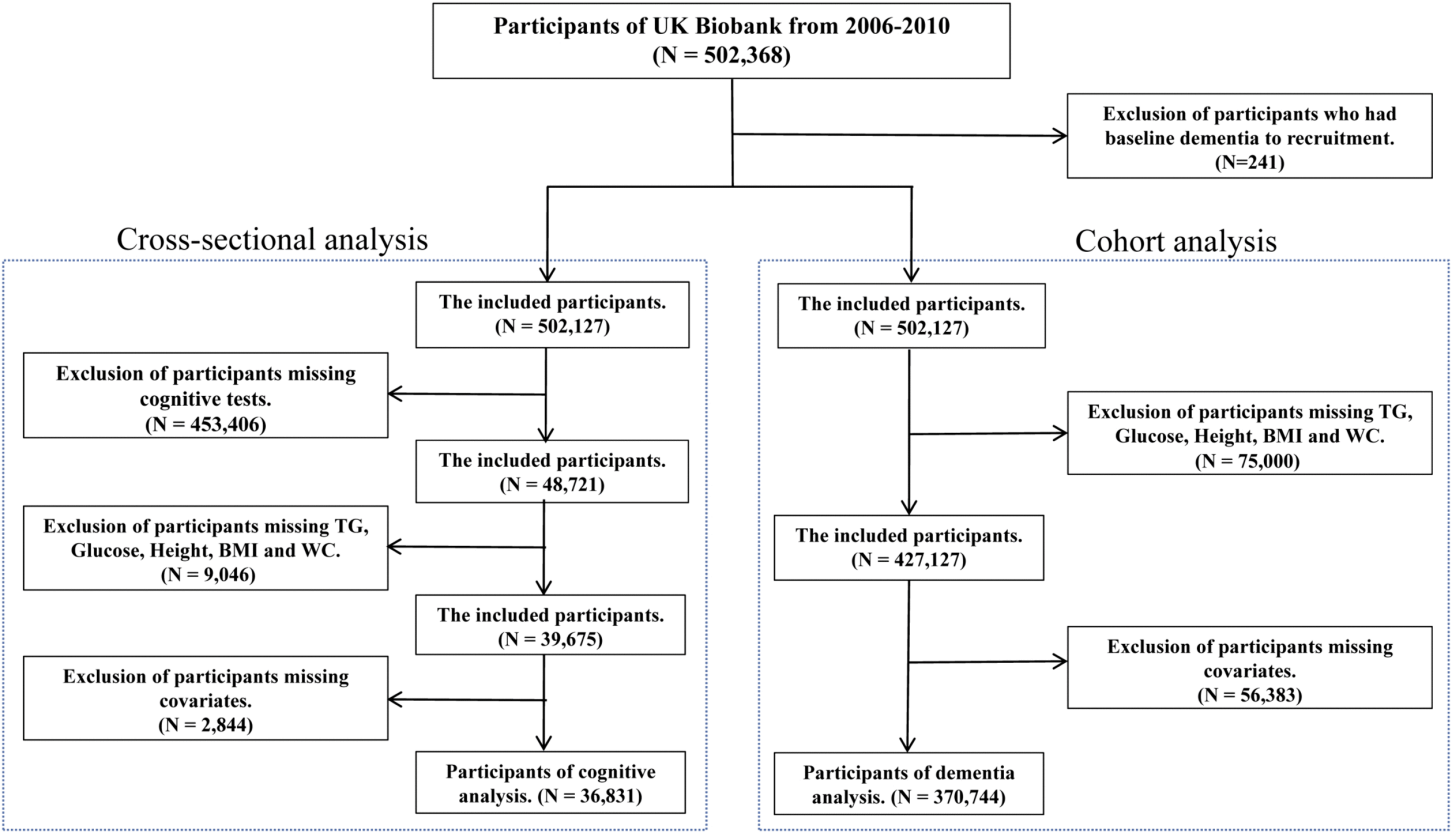
Flowchart of participant recruitment and exclusion criteria.

The study initially included 502,368 participants from the UK Biobank. We excluded 241 participants with pre-existing dementia. For the cross-sectional analysis examining the relationship between the TyG, ABSI, and TyG-ABSI indices and cognitive decline, 36,831 participants were ultimately included. Additionally, for the cohort study exploring the associations of the TyG, ABSI, and TyG-ABSI indices with dementia and its subtypes, 370,744 participants were ultimately included in the dementia analysis. Flowchart is shown in (Fig 1).

### 2.2 Assessment of TyG, ABSI and TyG-ABSI indices

In the UK Biobank, peripheral venous blood samples were collected from all participants at baseline, with the collection protocol validated for the UK Biobank study [20]. Collecting and processing fasting blood samples in large populations presents challenges; therefore, blood samples should be obtained randomly. Coefficients of variation for concentrations of TG, high sensitivity C-reactive protein, and creatinine were less than 3% and for glucose, total cholesterol, HDL-C, LDL-C, and uric acid were less than 2%. The TyG, ABSI, and TyG-ABSI indices were categorized into quartiles for analysis based on prior research, respectively [17]. TyG and ABSI were calculated using established formulas [21,22]:


$$\begin{array}{c} \text{TyG}=\text{ln}\;[\text{TG}\;(\text{mg}/\text{dL})\times \text{FPG}\;(\text{mg}/\text{dL})/\text{2}]; \end{array}$$

$$\begin{array}{c} \text{ABSI}=\text{WC}\;(\text{m})\;/\;[\text{BMI}\;{\left(\text{kg}/{\text{m}}^{\text{2}}\right)}^{\text{2}/\text{3}}\times \text{height}\;{\left(\text{m}\right)}^{\text{1}/\text{2}}]; \end{array}$$

$$\begin{array}{c} \text{TyG}-\text{ABSI}=\text{TyG}\times \text{ABSI}.\; \end{array}$$

### 2.3 Assessment of cognitive function

In the UK Biobank, self-administered computerized cognitive tests have been developed for population-scale assessment, including reaction time (RT), verbal-numerical reasoning, numeric memory, prospective memory, and reasoning [23]. Additionally, the details of the cognitive test scoring methods were described elsewhere [24]. We used the first principal component (explaining 43.03% variance) as the cognitive score, where higher values indicated better performance. Currently, no formal standard of cutoff point has been established to identify low cognitive function in the UK Biobank. In accordance with previously published studies [25,26], this study defined the 25th quantile of the cognitive test score as the cutoff point.

### 2.4 Assessment of dementia

The cases of ACD are identified based on the algorithmically defined outcomes (field 42018), and the cases of AD and VD are determined by the earliest first occurrence among the algorithmically- defined outcomes (fields 42020 and 42022) and the first occurrences (fields 130836 and 130838) [27]. Inpatient records came from hospital statistics databases in England, Scotland and Wales; death registry records were from the NHS and statistical authorities of the three countries. The ICD 10 codes used to ascertain dementia were selected and validated by the UK Biobank outcome adjudication group (Supplemental File Table S1 in S1 File).

### 2.5 Covariates

Covariates were selected based on prior studies [28–30]. Detailed definitions of these covariates were as follows: Age was categorized in accordance with the latest WHO criteria (≤65 years, >65 years), and sex was categorized as male or female; sociodemographic characteristics included race (White, other), residence (urban, rural), and educational level (college or above, others); lifestyle factors comprised drinking status (current, former, never) and smoking status (current, former, never); the socioeconomic indicator was TDI quartiles [31] (a composite deprivation measure); family or medical history included history of stroke (yes, no), history of anxiety (yes, no), and family history of diabetes (yes, no); laboratory measures (systolic blood pressure [SBP], high-density lipoprotein cholesterol [HDL-C], C-reactive protein [CRP]).

### 2.6 Statistical analysis

Baseline characteristics were analyzed after grouping by TyG‑ABSI quartiles: continuous variables were presented as median (interquartile range) and compared using the Kruskal‑Wallis test, while categorical variables were summarized as numbers (percentages) and compared using the χ² test. For variables with a significant overall test, post‑hoc pairwise comparisons were performed using Dunn’s test with Bonferroni correction for continuous variables and pairwise χ² tests with Bonferroni correction for categorical variables.

Kaplan-Meier curves were drawn to estimate cumulative incidence of dementia and its subtypes, with log-rank tests used to assess group differences. To explore the potential nonlinear dose-response relationships of TyG, ABSI, and TyG-ABSI indices with cognitive decline and dementia risk, restricted cubic spline (RCS) models were fitted with four knots placed at the 5th, 35th, 65th, and 95th percentiles of each index’s distribution. Logistic regression models were used to examine independent associations of TyG, ABSI and TyG-ABSI indices with cognitive decline, reported as adjusted odds ratios (ORs) and 95% confidence intervals (95% CIs). For Cox hazard regression models examining associations of TyG, ABSI and TyG-ABSI indices with ACD and subtypes, stratified Cox regressions were applied to covariates violating proportional hazards assumptions, reported as adjusted hazard ratios (HRs) and 95% CIs. The association between indices and outcomes was evaluated using two models: one for continuous variables (for each one-standard-deviation increase) and another for quartile grouping (using the lowest quartile as reference). Model 1 was unadjusted; Model 2 was adjusted for age, sex, race, and residence; Model 3 was further adjusted for educational level, drinking and smoking status, TDI, family history of diabetes, history of anxiety and stroke, SBP, HDL-C and CRP levels. The Pearson correlation coefficient and variance inflation factor (VIF) were used to assess the collinearity between TyG and ABSI. The results showed a correlation coefficient of 0.300 (95% CI: 0.297–0.303), with both VIF values below 2, indicating no collinearity issue. Evaluating diagnostic value using receiver operating characteristic (ROC) curves. The area under the ROC curve (AUC) was calculated to quantify the discriminative ability of each index. The 95% confidence interval (CI) of AUC was estimated using the DeLong method (a non-parametric approach for paired AUC comparisons). Comparisons between AUC values of different metrics were performed using the DeLong test.

Subgroup analyses were performed to examine the relationships of TyG, ABSI, and TyG-ABSI indices with cognitive decline, dementia and its subtypes across different groups, using fully adjusted models. Statistical significance of subgroup differences was evaluated by likelihood ratio tests for the interaction terms between each index and subgroup variable. Finally, several sensitivity analyses were conducted to assess the robustness of our findings: (1) excluding participants who developed dementia and its subtypes within 5 and 10 years of follow-up to control for reverse causality; (2) performing multiple imputation by chained equations for missing covariate values (Supplementary File Table S2 in S1 File); (3) removing outliers of five variables (TyG, ABSI, SBP, HDL, and CRP) using the interquartile range method to optimize data distribution; (4) adding sleep duration to the model based on relevant literature [32,33]; (5) fitting a competing risk model with death as a competing event for dementia and its subtypes [34]; (6) in the fully adjusted model, the two covariates HDL-C and CRP were removed, and the primary analysis was repeated to examine whether the results were affected by adjustment for these two metabolic indicators; (7) we additionally adjusted for HbA1c to reduce the potential influence of non-fasting blood glucose [35]; (8) sensitivity analyses were performed using alternative definitions of cognitive decline: bottom 20%, bottom 30%, and 1 SD below the mean.

Data cleaning was performed using Stata 18, and all statistical analyses were conducted using R (Version 4.4.3). Two-tailed tests were used to determine statistical significance, with a significance threshold of *P* < 0.05.

## 3. Results

### 3.1 Cognitive decline

Among the 36,831 participants included in the analysis of cognitive function baseline characteristics, all variables exhibited statistically significant differences between groups (*P* < 0.05; Table 1). Additionally, compared with the first quartile (Q1), participants in the fourth quartile (Q4) of TyG-ABSI were more likely to be older (>65 years), male, of non-white race, and urban-dwelling. They had lower educational qualifications and lower TDI, higher rates of current/previous smoking, current/previous drinking, a family history of diabetes, and a history of stroke, while their prevalence of anxiety was lower. Further categorization by disease status showed 9,197 individuals with cognitive decline (Supplementary File Table S3 in S1 File). For cognitive decline, post-hoc tests were conducted to compare pairwise baseline characteristics that showed significant differences in global assessments (Supplementary File Table S4 in S1 File).

**Table 1:** Baseline characteristics of cognitive decline categorized by TyG-ABSI index.

Characteristic	Total (N = 36831)	Q1 (N = 9213)	Q2 (N = 9206)	Q3 (N = 9215)	Q4 (N = 9197)	*P*-value
Age, n (%)						<0.001
≤65	31677 (86.01)	8456 (91.78)	7937 (86.22)	7783 (84.46)	7501 (81.56)	
>65	5154 (13.99)	757 (8.22)	1269 (13.78)	1432 (15.54)	1696 (18.44)	
Sex, n (%)						<0.001
Male	16954 (46.03)	1147 (12.45)	3407 (37.01)	5507 (59.76)	6893 (74.95)	
Female	19877 (53.97)	8066 (87.55)	5799 (62.99)	3708 (40.24)	2304 (25.05)	
Race, n (%)						<0.001
White	35614 (96.70)	8940 (97.04)	8933 (97.03)	8944 (97.06)	8797 (95.65)	
Others	1217 (3.30)	273 (2.96)	273 (2.97)	271 (2.94)	400 (4.35)	
Residence, n (%)						<0.001
Urban	27635 (75.03)	6772 (73.50)	6849 (74.40)	6972 (75.66)	7042 (76.57)	
Rural	9196 (24.97)	2441 (26.50)	2357 (25.60)	2243 (24.34)	2155 (23.43)	
Educational level, n (%)						<0.001
College/Above	11908 (32.33)	3285 (35.66)	2996 (32.54)	2832 (30.73)	2795 (30.39)	
Others	24923 (67.67)	5928 (64.34)	6210 (67.46)	6383 (69.27)	6402 (69.61)	
Smoking status, n (%)						<0.001
Current	3619 (9.83)	654 (7.10)	854 (9.28)	971 (10.54)	1140 (12.40)	
Previous	13142 (35.68)	2688 (29.18)	3073 (33.38)	3418 (37.09)	3963 (43.09)	
Never	20070 (54.49)	5871 (63.73)	5279 (57.34)	4826 (52.37)	4094 (44.51)	
Drinking status, n (%)						<0.001
Current	34183 (92.81)	8569 (93.01)	8556 (92.94)	8552 (92.81)	8506 (92.49)	
Previous	1328 (3.61)	286 (3.10)	308 (3.35)	346 (3.75)	388 (4.22)	
Never	1320 (3.58)	358 (3.89)	342 (3.71)	317 (3.44)	303 (3.29)	
TDI, n (%)						<0.001
Q1	9248 (25.11)	2488 (27.01)	2371 (25.75)	2300 (24.96)	2089 (22.71)	
Q2	9194 (24.96)	2438 (26.46)	2362 (25.66)	2242 (24.33)	2152 (23.40)	
Q3	9205 (24.99)	2279 (24.74)	2257 (24.52)	2310 (25.07)	2359 (25.65)	
Q4	9184 (24.94)	2008 (21.80)	2216 (24.07)	2363 (25.64)	2597 (28.24)	
History of anxiety, n (%)						<0.001
No	16379 (44.47)	3593 (39.00)	3994 (43.38)	4280 (46.45)	4512 (49.06)	
Yes	20452 (55.53)	5620 (61.00)	5212 (56.62)	4935 (53.55)	4685 (50.94)	
Family history of diabetes, n (%)						<0.001
No	30506 (82.83)	7819 (84.87)	7673 (83.35)	7604 (82.52)	7410 (80.57)	
Yes	6325 (17.17)	1394 (15.13)	1533 (16.65)	1611 (17.48)	1787 (19.43)	
History of stroke, n (%)						<0.001
No	35699 (96.93)	9042 (98.14)	8952 (97.24)	8926 (96.86)	8779 (95.46)	
Yes	1132 (3.07)	171 (1.86)	254 (2.76)	289 (3.14)	418 (4.54)	
SBP	82.00 (75.00, 90.00)	79.00 (72.00,86.00)	82.00 (75.00,89.00)	84.00 (77.00,91.00)	85.00 (78.00,92.00)	<0.001
HDL-C	1.41 (1.18, 1.68)	1.67 (1.43,1.94)	1.49 (1.27,1.73)	1.32 (1.15,1.54)	1.19 (1.03,1.39)	<0.001
CRP	1.31 (0.65, 2.67)	0.89 (0.47,1.86)	1.23 (0.61,2.54)	1.46 (0.75,2.89)	1.76 (0.93,3.37)	<0.001
TyG	8.72 (8.36, 9.12)	8.22 (7.98,8.46)	8.58 (8.34,8.82)	8.86 (8.61,9.12)	9.30 (9.02,9.61)	<0.001
ABSI	0.08 (0.07, 0.08)	0.07 (0.07,0.07)	0.07 (0.07,0.08)	0.08 (0.08,0.08)	0.08 (0.08,0.08)	<0.001
TyG-WC	778.65 (678.43, 880.93)	627.19 (580.59,680.62)	733.59 (683.79,790.94)	819.80 (766.52,879.90)	927.93 (861.12,1002.80)	<0.001
TyG-BMI	233.95 (205.25, 267.83)	202.61 (182.66,227.67)	224.73 (201.95,253.24)	242.89 (218.55,271.76)	265.46 (238.44,297.30)	<0.001
TyG-WHtR	4.60 (4.06, 5.17)	3.80 (3.52,4.13)	4.37 (4.07,4.73)	4.80 (4.49,5.17)	5.37 (4.98,5.84)	<0.001
TyG-ABSI	0.67 (0.61, 0.72)	0.58 (0.56,0.60)	0.64 (0.63,0.65)	0.69 (0.68,0.70)	0.75 (0.73,0.78)	<0.001

Abbreviations: Q1-Q4: Q1 to Q4 represent the first to fourth quartiles of the TyG-ABSI index in ascending order. TDI: Townsend Deprivation Index; SBP: Systolic Blood Pressure; HDL-C: High-Density Lipoprotein Cholesterol; CRP: C-Reactive Protein; TyG: Triglyceride glucose; ABSI: A body shape index; TyG-WC: Triglyceride-glucose index-Waist Circumference; TyG-BMI: Triglyceride-glucose index-Body Mass Index; TyG-WHtR: Triglyceride-glucose index-Waist-to-Height Ratio; TyG-ABSI: Triglyceride Glucose-A Body Shape Index.

As the TyG, ABSI and TyG-ABSI indices increased, the RCS of cognitive decline tended to rise (Supplementary File Fig. S1-S3 in S1 File). RCS analysis revealed a significant non-linear association between the TyG index and cognitive decline (*P*-non-linear < 0.05). In contrast, no statistically significant non-linear relationships were observed between the ABSI and the TyG-ABSI index, and cognitive decline.

In continuous analyses, each one–SD increase in the TyG, ABSI, and TyG-ABSI indices was associated with a higher risk of cognitive decline: OR = 1.06 (95% CI: 1.04–1.09; *P* < 0.001), OR = 1.13 (95% CI: 1.10–1.17; *P* < 0.001), and OR = 1.13 (95% CI: 1.10–1.16; *P* < 0.001), respectively (Supplementary File Table S5 in S1 File).

Consistent with these linear trends, the quartile analysis revealed that participants in the Q4 of TyG, ABSI, and TyG-ABSI had respective OR (95% CI) of 1.16 (95% CI: 1.07–1.25), 1.36 (95% CI: 1.25–1.47), and 1.36 (95% CI: 1.25–1.48) for cognitive decline compared with the Q1 (Table 2).

**Table 2:** Logistic results for quartile-categorized TyG, ABSI, and TyG-ABSI in relation to cognitive decline.

Variable	Model1		Model2		Model3	
	OR (95%CI)	*P*	OR (95%CI)	*P*	OR (95%CI)	*P*
**TyG**						
Q1	Ref.		Ref.		Ref.	
Q2	1.14 (1.06 ~ 1.22)	<0.001	1.13 (1.05 ~ 1.21)	<0.001	1.11 (1.04 ~ 1.19)	0.003
Q3	1.18 (1.11 ~ 1.27)	<0.001	1.20 (1.12 ~ 1.28)	<0.001	1.16 (1.07 ~ 1.24)	<0.001
Q4	1.18 (1.10 ~ 1.26)	<0.001	1.21 (1.13 ~ 1.30)	<0.001	1.16 (1.07 ~ 1.25)	<0.001
**ABSI**						
Q1	Ref.		Ref.		Ref.	
Q2	1.05 (0.98 ~ 1.12)	0.159	1.12 (1.04 ~ 1.20)	0.001	1.10 (1.03 ~ 1.18)	0.008
Q3	1.05 (0.98 ~ 1.12)	0.145	1.23 (1.15 ~ 1.33)	<0.001	1.20 (1.11 ~ 1.29)	<0.001
Q4	1.22 (1.14 ~ 1.30)	<0.001	1.44 (1.33 ~ 1.56)	<0.001	1.36 (1.25 ~ 1.47)	<0.001
**TyG-ABSI**						
Q1	Ref.		Ref.		Ref.	
Q2	1.11 (1.04 ~ 1.19)	0.002	1.16 (1.08 ~ 1.24)	<0.001	1.14 (1.06 ~ 1.22)	<0.001
Q3	1.15 (1.08 ~ 1.23)	<0.001	1.29 (1.20 ~ 1.39)	<0.001	1.25 (1.15 ~ 1.35)	<0.001
Q4	1.24 (1.16 ~ 1.33)	<0.001	1.42 (1.31 ~ 1.53)	<0.001	1.36 (1.25 ~ 1.48)	<0.001

Abbreviations: TyG: Triglyceride glucose; ABSI: A body shape index; TyG-ABSI: Triglyceride Glucose-A Body Shape Index; OR (95% CI): Odds Ratio (95% Confidence Interval); TDI: Townsend Deprivation Index; CRP: C-Reactive Protein; SBP: Systolic Blood Pressure; HDL-C: High-Density Lipoprotein Cholesterol.

Model1: Crude.

Model2: Adjusted for age, sex, race, and residence.

Model3: Age, sex, race, residence, educational level, smoking status and drinking status, TDI, history of anxiety, history of stroke, family history of diabetes, CRP, SBP, and HDL-C were adjusted for.

Subgroup analyses further demonstrated that the associations of the indices with cognitive decline varied by certain variables, with significant interaction effects observed (all *P* for interaction < 0.05; Supplementary File Tables S6-S8 in S1 File). Specifically, the association of TyG with cognitive decline was modified by sex, with a significant interaction effect; the association of ABSI with cognitive decline was modified by race, TDI, and history of anxiety, with significant interaction effects; and the association of TyG-ABSI with cognitive decline was modified by sex, race, with significant interaction effects. Additionally, consistent findings were observed in sensitivity analyses (Supplementary File Table S9-S14 in S1 File).

### 3.2 Dementia and its subtypes

Table 3 presented the baseline characteristics of dementia and its subtypes, categorized by TyG-ABSI. Further categorization by disease status showed 6,938 cases of ACD, 3,066 of AD, 1,545 of VD (Supplementary File Table S15-S17 in S1 File). For dementia, post-hoc tests were conducted to compare pairwise baseline characteristics that showed significant differences in global assessments(Supplementary File Table S17 in S1 File). For the baseline analysis (n = 370,744), follow-up duration reached approximately 18 years (median: 15.7 years), which was consistent across dementia and its subtypes. Compared with the Q1, participants in the Q4 of TyG-ABSI were more likely to be older (>65 years), male, of non-white race, reside in urban areas, have a lower education level and a higher TDI, current/previous smokers and previous/never drinkers, have anxiety, a family history of diabetes, or a history of stroke.

**Table 3:** Baseline characteristics of dementia categorized by TyG-ABSI index.

Characteristic	Total (N = 370,744)	Q1(N = 92,808)	Q2(N = 92,725)	Q3(N = 92,648)	Q4(N = 92,563)	*P*-value
Age, n (%)						<0.001
≤65	316,571 (85.39)	84,689 (91.25)	79,969 (86.24)	77,101 (83.22)	74,812 (80.82)	
>65	54,173 (14.61)	8,119 (8.75)	12,756 (13.76)	15,547 (16.78)	17,751 (19.18)	
Sex, n (%)						<0.001
Male	170,349 (45.95)	11,894 (12.82)	34,422 (37.12)	54,306 (58.62)	69,727 (75.33)	
Female	200,395 (54.05)	80,914 (87.18)	58,303 (62.88)	38,342 (41.38)	22,836 (24.67)	
Race, n (%)						<0.001
White	352,090 (94.97)	87,692 (94.49)	88,109 (95.02)	88,388 (95.40)	87,901 (94.96)	
Others	18,654 (5.03)	5,116 (5.51)	4,616 (4.98)	4,260 (4.60)	4,662 (5.04)	
Residence, n (%)						<0.001
Urban	318,077 (85.79)	79,031 (85.16)	79,358 (85.58)	79,576 (85.89)	80,112 (86.55)	
Rural	52,667 (14.21)	13,777 (14.84)	13,367 (14.42)	13,072 (14.11)	12,451 (13.45)	
Educational level, n (%)						<0.001
College/Above	121,246 (32.70)	34,802 (37.50)	30,898 (33.32)	29,136 (31.45)	26,410 (28.53)	
Others	249,498 (67.30)	58,006 (62.50)	61,827 (66.68)	63,512 (68.55)	66,153 (71.47)	
Smoking status, n (%)						<0.001
Current	38,178 (10.30)	6,845 (7.38)	8,852 (9.55)	10,067 (10.87)	12,414 (13.41)	
Previous	129,424 (34.91)	27,397 (29.52)	30,502 (32.90)	33,697 (36.37)	37,828 (40.87)	
Never	203,142 (54.79)	58,566 (63.10)	53,371 (57.56)	48,884 (52.76)	42,321 (45.72)	
Drinking status, n (%)						<0.001
Current	342,037 (92.26)	86,020 (92.69)	85,696 (92.42)	85,630 (92.43)	84,691 (91.50)	
Previous	13,061 (3.52)	2,830 (3.05)	3,054 (3.29)	3,230 (3.49)	3,947 (4.26)	
Never	15,646 (4.22)	3,958 (4.26)	3,975 (4.29)	3,788 (4.09)	3,925 (4.24)	
TDI, n (%)						<0.001
Q1	93,125 (25.12)	24,387 (26.28)	23,765 (25.63)	23,251 (25.10)	21,722 (23.47)	
Q2	92,549 (24.96)	23,736 (25.58)	23,230 (25.05)	23,226 (25.07)	22,357 (24.15)	
Q3	92,530 (24.96)	23,226 (25.03)	23,095 (24.91)	23,142 (24.98)	23,067 (24.92)	
Q4	92,540 (24.96)	21,459 (23.12)	22,635 (24.41)	23,029 (24.86)	25,417 (27.46)	
History of anxiety, n (%)						<0.001
No	162,265 (43.77)	36,116 (38.91)	39,576 (42.68)	42,370 (45.73)	44,203 (47.75)	
Yes	208,479 (56.23)	56,692 (61.09)	53,149 (57.32)	50,278 (54.27)	48,360 (52.25)	
Family history of diabetes, n (%)						<0.001
No	306,043 (82.55)	78,049 (84.10)	76,996 (83.04)	76,358 (82.42)	74,640 (80.64)	
Yes	64,701 (17.45)	14,759 (15.90)	15,729 (16.96)	16,290 (17.58)	17,923 (19.36)	
History of stroke, n (%)						<0.001
No	357,843 (96.52)	90,942 (97.99)	89,855 (96.90)	89,106 (96.18)	87,940 (95.01)	
Yes	12,901 (3.48)	1,866 (2.01)	2,870 (3.10)	3,542 (3.82)	4,623 (4.99)	
SBP	82 (75.00,89.00)	78 (72.00,86.00)	81 (75.00,89.00)	83 (76.00,90.00)	84 (77.00,91.00)	<0.001
HDL-C	1.40 (1.17,1.68)	1.67 (1.44,1.93)	1.48 (1.27,1.72)	1.33 (1.14,1.55)	1.18 (1.02,1.37)	<0.001
CRP	1.32 (0.66,2.75)	0.89 (0.45,1.89)	1.24 (0.61,2.62)	1.47 (0.76,2.98)	1.79 (0.95,3.43)	<0.001
TyG	8.68 (8.31,9.07)	8.17 (7.94,8.41)	8.53 (8.29,8.78)	8.82 (8.57,9.07)	9.27 (8.99,9.58)	<0.001
ABSI	0.08 (0.07,0.08)	0.07 (0.07,0.07)	0.08 (0.07,0.08)	0.08 (0.08,0.08)	0.08 (0.08,0.08)	<0.001
TyG-WC	781.72 (680.71,884.89)	628.59 (581.27,684.06)	736.64 (685.96,795.36)	822.97 (769.22,883.19)	931.40 (866.06,1,009.51)	<0.001
TyG-BMI	233.08 (204.50, 267.04)	201.45 (181.88, 226.68)	223.82 (201.51, 252.19)	241.79 (218.10,270.38)	264.76 (238.29,296.32)	<0.001
TyG-WHtR	4.63 (4.08,5.20)	3.81 (3.53,4.15)	4.39 (4.09, 4.76)	4.82 (4.51,5.20)	5.40 (5.02,5.87)	<0.001
TyG-ABSI	0.67 (0.62,0.72)	0.58 (0.56, 0.60)	0.64 (0.63, 0.66)	0.69 (0.68, 0.71)	0.75 (0.73,0.78)	<0.001

Abbreviations: Q1-Q4: Q1 to Q4 represent the first to fourth quartiles of the TyG-ABSI index in ascending order. TDI: Townsend Deprivation Index; SBP: Systolic Blood Pressure; HDL-C: High-Density Lipoprotein Cholesterol; CRP: C-Reactive Protein; TyG: Triglyceride glucose; ABSI: A body shape index; TyG-WC: Triglyceride-glucose index-Waist Circumference; TyG-BMI: Triglyceride-glucose index-Body Mass Index; TyG-WHtR: Triglyceride-glucose index-Waist-to-Height Ratio; TyG-ABSI: Triglyceride Glucose-A Body Shape Index.

**Fig 2:**
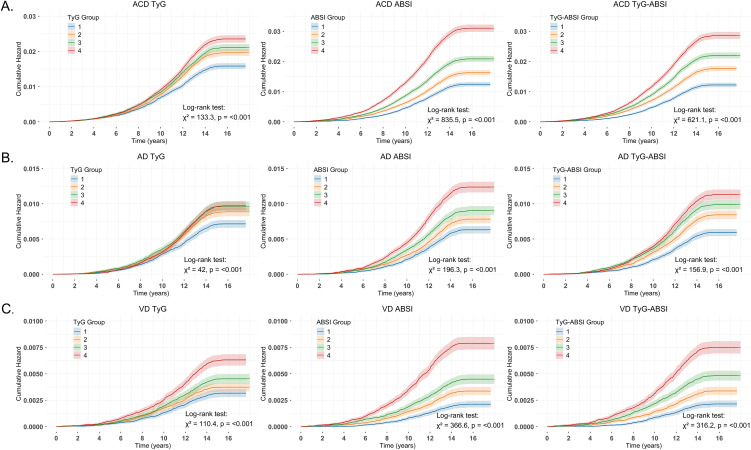
Kaplan-Meier survival curves for all-cause dementia (ACD), Alzheimer’s disease (AD), and vascular dementia (VD).

Kaplan-Meier curves showed cumulative incidence of ACD and its subtypes increased with higher TyG, ABSI and TyG-ABSI quartiles (all log-rank *P* < 0.001; Fig 2). Moreover, for each dementia subtype, cumulative hazard exhibited the steepest increase with rising ABSI levels, followed by TyG-ABSI, and then TyG. This indicated that, in comparison to TyG and TyG-ABSI, the elevation in cumulative risk for dementia and its subtypes was most pronounced with increasing ABSI index.

With increasing TyG, ABSI and TyG -ABSI indices, the RCS of ACD and its subtypes all showed an upward trend (Supplementary File Fig.S1-S3 in S1 File). Specifically, RCS analysis revealed a significant non-linear association between the TyG index and each of ACD, AD, and VD (all *P*-non-linear < 0.05). Second, ABSI showed a non-linear relationship with ACD (*P*-non-linear < 0.05) but not with AD or VD. Additionally, no statistically significant non-linear relationships existed between the TyG-ABSI index and ACD, AD, or VD.

In continuous analyses, each one-SD increase in TyG, ABSI, and TyG-ABSI was associated with a higher risk of dementia and its subtypes (Supplementary File Table S19 in S1 File). Under the fully adjusted model, the HR (95% CI) for ACD were 1.09 (1.06–1.12), 1.26 (1.22–1.30), and 1.24 (1.21–1.28); for AD were 1.08 (1.04–1.13), 1.20 (1.15–1.25), and 1.19 (1.14–1.25); and for VD were 1.19 (1.12–1.26), 1.29 (1.21–1.37), and 1.34 (1.26–1.43), respectively (all *P* < 0.001).

After adjusting for all potential confounding variables (Table 4), in Model 3 of ACD, using Q1 as the reference group, participants in Q4 of TyG, ABSI and TyG-ABSI had HR of 1.33 (95% CI: 1.13–1.57), 1.79 (95% CI: 1.65–1.94) and 1.67 (95% CI: 1.54–1.82); For AD, using Q1 as the reference group, participants in Q4 of TyG, ABSI and TyG-ABSI had HR of 1.14 (95% CI: 1.02–1.29), 1.58 (95% CI: 1.39–1.78) and 1.54 (95% CI: 1.36–1.75); For VD, using Q1 as the reference group, participants in Q4 of TyG, ABSI and TyG-ABSI had HR of 1.18 (95% CI: 1.09–1.27), 1.98 (95% CI: 1.64–2.40) and 1.67 (95% CI: 1.54–1.82), respectively.

**Table 4:** Cox model results for quartile-categorized TyG, ABSI and TyG-ABSI with ACD and its subtypes.

Classify	Model1		Model2		Model3	
	HR (95%CI)	*P*	HR (95%CI)	*P*	HR (95%CI)	*P*
**TyG**						
ACD						
Q1	Ref.		Ref.		Ref.	
Q2	1.18 (1.01 ~ 1.39)	0.039	0.98 (0.84 ~ 1.15)	0.829	0.95 (0.81 ~ 1.12)	0.572
Q3	1.43 (1.23 ~ 1.67)	<0.001	1.10 (0.95 ~ 1.29)	0.207	1.05 (0.89 ~ 1.23)	0.557
Q4	2.00 (1.73 ~ 2.30)	<0.001	1.50 (1.30 ~ 1.74)	<0.001	1.33 (1.13 ~ 1.57)	<0.001
AD						
Q1	Ref.		Ref.		Ref.	
Q2	1.24 (1.12 ~ 1.38)	<0.001	1.06 (0.95 ~ 1.18)	0.301	1.07 (0.97 ~ 1.20)	0.191
Q3	1.35 (1.22 ~ 1.50)	<0.001	1.10 (0.99 ~ 1.22)	0.072	1.13 (1.01 ~ 1.26)	0.027
Q4	1.35 (1.22 ~ 1.50)	<0.001	1.11 (1.00 ~ 1.24)	0.045	1.14 (1.02 ~ 1.29)	0.023
VD						
Q1	Ref.		Ref.		Ref.	
Q2	1.24 (1.15 ~ 1.33)	<0.001	1.06 (0.98 ~ 1.13)	0.124	1.06 (0.99 ~ 1.14)	0.095
Q3	1.33 (1.24 ~ 1.43)	<0.001	1.07 (1.00 ~ 1.15)	0.046	1.09 (1.01 ~ 1.17)	0.029
Q4	1.48 (1.38 ~ 1.59)	<0.001	1.19 (1.11 ~ 1.27)	<0.001	1.18 (1.09 ~ 1.27)	<0.001
**ABSI**						
ACD						
Q1	Ref.		Ref.		Ref.	
Q2	1.31 (1.21 ~ 1.41)	<0.001	1.22 (1.13 ~ 1.33)	<0.001	1.21 (1.12 ~ 1.31)	<0.001
Q3	1.68 (1.56 ~ 1.81)	<0.001	1.48 (1.36 ~ 1.60)	<0.001	1.42 (1.31 ~ 1.54)	<0.001
Q4	2.50 (2.33 ~ 2.69)	<0.001	1.95 (1.80 ~ 2.12)	<0.001	1.79 (1.65 ~ 1.94)	<0.001
AD						
Q1	Ref.		Ref.		Ref.	
Q2	1.24 (1.11 ~ 1.38)	<0.001	1.18 (1.06 ~ 1.33)	0.003	1.19 (1.06 ~ 1.33)	0.003
Q3	1.43 (1.28 ~ 1.60)	<0.001	1.32 (1.18 ~ 1.49)	<0.001	1.31 (1.17 ~ 1.48)	<0.001
Q4	1.97 (1.78 ~ 2.18)	<0.001	1.63 (1.45 ~ 1.84)	<0.001	1.58 (1.39 ~ 1.78)	<0.001
VD						
Q1	Ref.		Ref.		Ref.	
Q2	1.60 (1.33 ~ 1.92)	<0.001	1.41 (1.17 ~ 1.70)	<0.001	1.33 (1.10 ~ 1.60)	0.003
Q3	2.13 (1.79 ~ 2.54)	<0.001	1.66 (1.38 ~ 2.00)	<0.001	1.45 (1.20 ~ 1.76)	<0.001
Q4	3.77 (3.21 ~ 4.44)	<0.001	2.47 (2.05 ~ 2.97)	<0.001	1.98 (1.64 ~ 2.40)	<0.001
**TyG-ABSI**						
ACD						
Q1	Ref.		Ref.		Ref.	
Q2	1.44 (1.34 ~ 1.56)	<0.001	1.23 (1.14 ~ 1.33)	<0.001	1.23 (1.13 ~ 1.33)	<0.001
Q3	1.80 (1.67 ~ 1.94)	<0.001	1.40 (1.29 ~ 1.51)	<0.001	1.40 (1.29 ~ 1.52)	<0.001
Q4	2.34 (2.18 ~ 2.51)	<0.001	1.70 (1.57 ~ 1.84)	<0.001	1.67 (1.54 ~ 1.82)	<0.001
AD						
Q1	Ref.		Ref.		Ref.	
Q2	1.42 (1.27 ~ 1.59)	<0.001	1.24 (1.10 ~ 1.38)	<0.001	1.26 (1.12 ~ 1.41)	<0.001
Q3	1.68 (1.50 ~ 1.87)	<0.001	1.36 (1.22 ~ 1.53)	<0.001	1.41 (1.25 ~ 1.59)	<0.001
Q4	1.91 (1.72 ~ 2.13)	<0.001	1.49 (1.33 ~ 1.68)	<0.001	1.54 (1.36 ~ 1.75)	<0.001
VD						
Q1	Ref.		Ref.		Ref.	
Q2	1.44 (1.34 ~ 1.56)	<0.001	1.23 (1.14 ~ 1.33)	<0.001	1.23 (1.13 ~ 1.33)	<0.001
Q3	1.80 (1.67 ~ 1.94)	<0.001	1.40 (1.29 ~ 1.51)	<0.001	1.40 (1.29 ~ 1.52)	<0.001
Q4	2.34 (2.18 ~ 2.51)	<0.001	1.70 (1.57 ~ 1.84)	<0.001	1.67 (1.54 ~ 1.82)	<0.001

Abbreviations: TyG: Triglyceride glucose; ABSI: A body shape index; TyG-ABSI: Triglyceride Glucose-A Body Shape Index; HR (95% CI): Hazard Ratio (95% Confidence Interval); TDI: Townsend Deprivation Index; CRP: C-Reactive Protein; SBP: Systolic Blood Pressure; HDL-C: High-Density Lipoprotein Cholesterol.

Model1: Crude.

Model2: Adjusted for age, sex, race, and residence.

Model3: Age, sex, race, residence, educational level level, smoking status and drinking status, TDI, history of anxiety, history of stroke, family history of diabetes, CRP, SBP, and HDL-C were adjusted for.

Subgroup analyses further revealed significant interaction effects (*P* for interaction < 0.05; Supplementary File Tables S20-S28 in S1 File): For the association with ACD, TyG was significantly interacted by age, sex, drinking status, and history of stroke; ABSI was significantly interacted by age, drinking status, history of anxiety and stroke; and TyG-ABSI was significantly interacted by age, sex, TDI, and history of stroke. For the association with AD, TyG was significantly interacted by age and sex; ABSI was significantly interacted by age and race; and TyG-ABSI was significantly interacted by age. For the association with VD, TyG was significantly interacted by age and sex; ABSI was significantly interacted by age, educational level, and history of stroke; and TyG-ABSI was significantly interacted by age and history of stroke. Finally, consistent findings were observed in sensitivity analyses (Supplementary File Tables S29-S36 in S1 File).

### 3.3 ROC analysis

**Fig 3:**
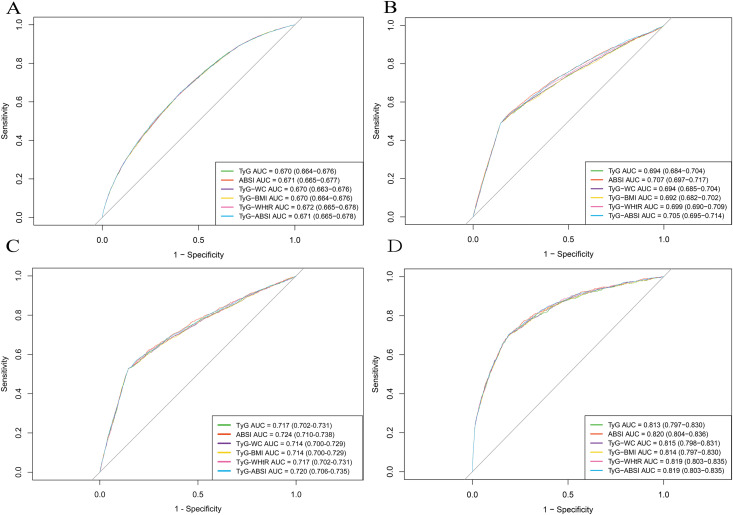
ROC for different IR surrogate indices to predict cognitive decline (A), all-cause dementia (B), Alzheimer’s disease (C) and vascular dementia (D).

ROC curves were derived from the fully adjusted Model 3 (Fig 3). For cognitive decline (3A), ABSI and TyG-ABSI showed nearly identical discriminative ability (AUC = 0.671), marginally lower than that of TyG-WHtR (AUC = 0.672). For ACD (3B), ABSI (0.707) and TyG-ABSI (0.705) demonstrated higher AUC than TyG (0.694), TyG-WC (0.694), TyG-BMI (0.692) and TyG-WHtR (0.699). For AD (3C), the AUC values of ABSI (0.724) and TyG-ABSI (0.720) were slightly higher than those of TyG (0.717), TyG-WC (0.714) and TyG-BMI (0.714) and TyG-WHtR (0.717). For VD (3D), ABSI yielded the highest AUC (0.820), followed by TyG-ABSI (0.819), TyG-WHtR (0.819), TyG-WC (0.815) and TyG-BMI (0.814), all of which outperformed the simple TyG index (0.813).

## 4. Discussion

The key findings of this study are as follows: (1) Both the TyG index and ABSI were significantly associated with cognitive decline, dementia, and all its subtypes. Notably, this study provided critical longitudinal evidence for the temporal association between ABSI and dementia. (2) The TyG-ABSI index, generated by combining the TyG index and ABSI, also exhibited a significant association with cognitive impairment, dementia, and its subtypes. (3) For prediction performance, ABSI had the relatively highest AUC, outperforming the TyG index, its derived indicators, and combined TyG-ABSI.

The results of this study revealed that both continuous and quartile-based TyG, ABSI, and TyG-ABSI indices were significantly positively associated with cognitive decline and dementia. In recent years, accumulating evidence within the field of cognitive research has increasingly highlighted the potential influence of metabolic and adiposity markers. A meta-analysis has demonstrated a positive correlation between the TyG index and the risk of cognitive impairment and dementia [36]. Similarly, research conducted among elderly populations has consistently indicated that individuals in the Q4 of the TyG index have a significantly elevated risk of cognitive impairment compared to those in the Q1 [37], findings that align with our results. The TyG index is a simple and reliable marker of insulin resistance; its contribution to cognitive decline and dementia likely arises from impairing cerebral insulin-signaling pathways and from exacerbating neurodegenerative processes through the induction of oxidative stress and inflammatory responses [38,39]. Additionally, the study results of Szu-Han Huang [40] and another cross-sectional study [41] respectively demonstrate that higher ABSI values are significantly associated with lower cognitive scores and a higher risk of dementia. As an indicator that is not influenced by the “obesity paradox,” ABSI provides a more precise reflection of body fat distribution, particularly in terms of VAT accumulation [22,42]. ABSI may impact cognition and dementia through two key mechanisms: elevated ABSI triggers chronic inflammation and oxidative stress, with pro-inflammatory mediators crossing the blood–brain barrier to induce neuroinflammation and cognitive impairment [43,44]. It also disrupts brain function by inducing gut microbiota dysbiosis, which affects the central nervous system via the vagus nerve [45].

Recent studies have combined the TyG index with obesity markers (BMI, WC, WHtR) to enhance diagnostic accuracy of related diseases, and ABSI is no exception. For example, a cohort study found that the combined effect of TyG and ABSI had a significant impact on stroke risk [16]. However, no systematic study has examined the dose-response relationship and predictive performance of TyG-ABSI for cognitive decline and dementia. To address this research gap, we conducted a related analysis to evaluate the clinical utility of TyG-ABSI in predicting cognition and dementia. Finally, the results indicate that the TyG-ABSI index not only has a significant impact on cognitive decline and dementia but also exhibits superior predictive value compared to the individual TyG index or its derived indices (TyG-BMI, TyG-WC and TyG-WHtR) in ACD and AD, similar with the study by Hao-Ming He [17]. These findings can be explained through several mechanisms. Impairing vascular function (via endothelial dysfunction and atherosclerosis) to reduce cerebral blood flow [46,47]; and directly damaging neural function by regulating A*β* deposition, compromising synaptic integrity, triggering neuroinflammation, and inducing pathological tau phosphorylation and neuronal death [47,48]. These combined effects cause neurodegenerative changes and increase cognitive decline and dementia risk [49].

RCS analyses revealed distinct dose-response patterns: TyG demonstrated significant non-linear associations with cognitive decline and all dementia subtypes, exhibiting threshold effects particularly for AD and VD at higher levels, which aligns closely with the results of numerous recent large-scale studies and meta-analyses [36,50]. In contrast, ABSI showed a specific non-linear association only with all-cause dementia, suggesting distinct pathological pathways. These findings underscore the complementary value of metabolic and morphological indicators for dementia risk stratification.

The Kaplan-Meier analysis consistently showed a lower cumulative risk for TyG-ABSI than for ABSI alone across subgroups, reinforcing ABSI’s superior predictive utility. Therefore, based on this finding and the ROC curve analysis results, ABSI—as a body shape indicator independent of BMI—may be more accurate than the combined metabolic-obesity index in identifying brain health risks associated with central fat distribution [15,51]. Furthermore, multiple imaging and pathological studies have demonstrated that the mechanisms by which obesity leads to cerebral atrophy and cognitive decline are independent of the AD-associated protein pathways (amyloid-β and tau) and are more likely related to cerebrovascular factors or the physiobiological effects of adipose tissue itself [52]. Consequently, the TyG variable reflecting metabolic abnormalities may account for only a small portion of obesity-related brain damage, whereas the morphological information on fat distribution captured by ABSI contributes to independent risk prediction capabilities. It implies that central body fat distribution, as measured by ABSI, may hold more direct or proximal relevance to the neuropathological substrates of dementia than a combined metabolic-morphological score. This is supported by growing evidence highlighting the unique detrimental role of VAT on brain health [53]. For instance, a recent large-scale study demonstrated that VAT had the strongest association with altered brain structure and accelerated brain aging compared to fat stored in other regions [54]. Further research is needed to elucidate the underlying mechanisms for this counterintuitive result.

Additionally, subgroup analysis further clarified the complexity of the associations between the three indices and cognitive decline, dementia, and its subtypes, which are modulated by demographic and related factors. For ACD, advancing age amplifies the brain-damaging effects of abnormal index levels due to the decline in metabolic regulation and neuroprotective mechanisms [55]. The associations demonstrated gender heterogeneity, likely influenced by sex-specific hormonal factors, which is consistent with previous reports of a more pronounced impact of midlife obesity and insulin resistance on cognition in women [56], and that postmenopausal women face an increased risk of cognitive impairment [57,58]. Alcohol exacerbates metabolic stress, while stroke directly damages brain tissue and forms a “synergistic effect” with metabolic disorders—both factors enhance the risk prediction role of the indices [59,60]. This aligns with prior findings that alcohol affects metabolic pathways and that stroke is associated with vascular damage [61]. The associations of metabolic indices with AD and VD are also modulated by multiple factors, with differences in modulating factors linked to the pathological characteristics of the subtypes. For example, ethnicity modifies the effect of ABSI in AD [62], and education exerts an influence through cognitive reserve in VD [63]. However, a large number of interaction effects may be affected by certain issues, which requires verification by larger cohorts and mechanistic studies.

Notably, HDL-C and CRP were included as covariates in Model 3. From a biological perspective, these variables may act as mediators rather than pure confounders, because insulin resistance can dysregulate lipid metabolism and provoke systemic inflammation; adjusting for them could lead to over-adjustment and underestimation. However, sensitivity analyses excluding these two covariates yielded results essentially unchanged from the primary analysis, indicating that the observed associations are robust and not materially influenced by the inclusion of these two potential mediators.

This study provides the first longitudinal evidence on the ABSI-dementia link and introduces the novel application of the combined TyG-ABSI index for predicting cognitive outcomes. Furthermore, this study offers strengths such as a large sample (more than 370,000 individuals) and a long follow-up period (median 15.7 years). Despite the achievements of this study, several limitations should be acknowledged. First, the study sample was primarily drawn from the United Kingdom, which may limit the generalizability of the findings. Second, the assessment of cognitive function primarily employed standardized psychometric tools and lacked support from objective indicators such as neuroimaging, but these instruments have been validated against imaging-based diagnoses and remain the primary screening method in large-scale epidemiological studies. Third, the frequency and interval of repeated cognitive assessments in the UK Biobank were insufficient to support robust cross-time lag analyses in this study population. Fourth, this study cannot completely exclude all confounding factors; however, on the basis of a large sample size, confounding factors were controlled to the greatest extent possible, and all covariates were assessed at baseline. This approach may not fully capture the long-term dynamic changes in health status and behavior during the follow-up period. Additionally, the TyG-related indicators and ABSI were measured only at baseline, preventing us from further investigating the dynamic changes between these indices and dementia disease risk. The TyG-related indicators were calculated using non-fasting blood glucose levels, which may influence TyG levels. Based on previous studies [35], we conducted a sensitivity analysis in the fully adjusted model with additional adjustment for HbA1c, yielding results consistent with the primary findings. Finally, Cognitive function decline was defined based on the 25th percentile of baseline scores (single assessment). Although no prior reference data were available, sensitivity analyses using thresholds of 20%, 30%, and below one standard deviation yielded consistent results; adjustments for age and education were made. Future studies require replication assessments for validation. Additionally, future research should expand the sample to diverse populations globally to validate the generalizability of the TyG-ABSI index. Incorporating objective measures like neuroimaging could elucidate the mechanisms linking TyG-ABSI to cognitive decline and dementia. Comparing ABSI with other obesity indicators and dynamically tracking changes in TyG and ABSI indices would provide stronger evidence for clinical application. To exclude the influence of reverse causality, the study excluded participants who developed dementia and its subtypes during the 5–10 year follow-up period, with results unchanged from the primary analysis. Nevertheless, future studies using Mendelian randomization (MR) are recommended to validate these associations and determine causal direction.

## 5. Conclusion

In UK Biobank, revealed significant associations between the TyG index, ABSI and the combined TyG-ABSI index with cognitive decline and dementia risk. The ABSI showed superior predictive performance for cognitive decline and dementia, thereby offering a powerful tool for early risk detection.

## Supporting information

S1 File Additional file (Supplementary tables, figures). (DOCX)

S2 File Graphical abstarct. (TIF)

## Abbreviations

ABSI: a body shape index
ACD: all-cause dementia
AD: alzheimer’s disease
BMI: body mass index
FPG: fasting plasma glucose
IR: insulin resistance
RCS: restricted cubic spline
ROC: receiver Operating Characteristic
TG: triglycerides
TyG: triglyceride-glucose index
TyG-ABSI: Triglyceride Glucose-A Body Shape Index
TyG-BMI: Triglyceride-glucose index-Body Mass Index
TyG-WC: Triglyceride-glucose index-Waist Circumference
TyG-WHtR: Triglyceride-glucose index-Waist-to-Height Ratio
VC: vascular dementia
WC: waist circumference
WHtR: waist-to-height ratio.
