# Supplementary material for: Association of triglyceride-glucose index (TyG) and a body shape index (ABSI) with cognitive decline and dementia risk
Source: PLoS One. 2026 Jul 22;21(7):e0354418. doi: 10.1371/journal.pone.0354418 (PMC13390943; doi:10.1371/journal.pone.0354418)
Supplement: S1 File — (DOCX) [file pone.0354418.s001.docx]

**Supplementary Materials**

**Table S1** International Classification of Disease codes used to ascertain dementia.

**Table S2** The proportion of missing covariates and imputation methods.

**Table S3** The baseline characteristics of cognitive decline stratified by the presence and absence of the condition.

**Table S4** For cognitive decline, post-hoc tests were conducted to compare pairwise baseline characteristics that showed significant differences in global assessments.

**Table S5** Logistic results of standardized continuous TyG, ABSI, and TyG-ABSI associated with cognition decline.

**Table S6-8** Subgroup analysis for TyG, ABSI and TyG-ABSI indices of cognitive decline.

**Table S9** Sensitivity analysis for logistic regression analyses of TyG,ABSI and TyG-ABSI indices with cognitive decline post-imputation.

**Table S10** Sensitivity analysis for performing logistic regression analyses of TyG,ABSI and TyG-ABSI indices with cognitive decline after removing outliers.

**Table S11** Sensitivity analysis for performing logistic regression analyses of TyG, ABSI and TyG-ABSI indices with cognitive decline after adding sleep duration to the full adjustment model.

**Table S12** Sensitivity analysis for Logistic regression analyses of TyG, ABSI and TyG-ABSI indices with cognitive decline was performed without adjusting for HDL-C and CRP in the fully adjusted model.

**Table S13** Sensitivity analysis for Logistic regression analyses of TyG, ABSI and TyG-ABSI indices with cognitive decline was performed additionally adjusting for glycated haemoglobin (HbA1c) in the fully adjusted model.

**Table S14** Sensitivity analysis for TyG, ABSI, and TyG-ABSI indices with cognitive decline (bottom 20%, bottom 30%, and 1 SD below mean).

**Table S15-17** The baseline characteristics of all-cause dementia, Alzheimer's dementia and vascular dementia stratified by disease presence and absence.

**Table S18** For dementia, post-hoc tests were conducted to compare pairwise baseline characteristics that showed significant differences in global assessments.

**Table S19** Cox for standardized continuous TyG, ABSI, and TyG-ABSI associated with dementia and its subtypes.

**Table S20-28** Subgroup analysis for TyG, ABSI and TyG-ABSI indices of all-cause dementia, Alzheimer's dementia and vascular dementia.

**Table S29** Sensitivity analysis for Cox regression analyses of TyG,ABSI and TyG-ABSI indices with dementia and subtypes excluding participants who developed dementia within 5 years after the follow-up period.

**Table S30** Sensitivity analysis for Cox regression analyses of TyG,ABSI and TyG-ABSI indices with dementia and subtypes excluding participants who developed dementia within 10 years after the follow-up period.

**Table S31** Sensitivity analysis for Cox regression analyses of TyG,ABSI and TyG-ABSI indices with dementia and subtypes post-imputation.

**Table S32** Sensitivity analysis for performing Cox regression analyses of TyG,ABSI and TyG-ABSI indices with dementia and subtypes after removing outliers.

**Table S33** Sensitivity analysis for performing Cox regression analyses of TyG, ABSI and TyG-ABSI indices with dementia and subtypes after adding sleep duration to the full adjustment model.

**Table S34** Sensitivity analysis was performed using a Fine-Gray model to evaluate the robustness of the association between indices and dementia and subtypes.

**Table S35** Sensitivity analysis for Cox regression analyses of TyG, ABSI and TyG-ABSI indices with dementia and subtypes was performed without adjusting for HDL-C and CRP in the fully adjusted model.

**Table S36** Sensitivity analysis for Cox regression analyses of TyG, ABSI and TyG-ABSI indices with dementia and subtypes was performed additionally adjusting for glycated haemoglobin (HbA1c) in the fully adjusted model.

**Fig. S1-3** RCS curves for TyG, ABSI and TyG-ABSI indices with cognitive function, all-cause dementia, Alzheimer's dementia and vascular dementia (TyG: A; ABSI: B; TyG-ABSI: C).

**Table S1** International Classification of Disease codes used to ascertain dementia.

| ICD-9 | ICD-10 |
| --- | --- |
| 331.0, 290.4, 331.1, 290.2, 290.3, 291.2, 294.1, 331.2, 331.5 | F00, F00.0, F00.1, F00.2, F00.9, G30, G30.0, G30.1, G30.8, G30.9, F01, F01.0, F01.1, F01.2, F01.3, F01.8, F01.9, I67.3, F02.0, G31.0, A81.0, F02, F02.1, F02.2, F02.3, F02.4, F02.8, F03, F05.1, F10.6, G31.1, G31.8 |

| **Table S2** The proportion of missing covariates and imputation methods. | | | |
| --- | --- | --- | --- |
| Variable | Number of missing data | Percentage of missing data (%) | Imputation methods |
| Race | 1977 | 0.46 | logreg |
| Residence | 4322 | 1.01 | logreg |
| Educational level | 5040 | 1.18 | logreg |
| Smoking status | 2126 | 0.50 | polyreg |
| Drinking status | 1023 | 0.24 | polyreg |
| TDI | 520 | 0.12 | pmm |
| History of anxiety | 12242 | 2.87 | logreg |
| Family history of diabetes | 7054 | 1.65 | logreg |
| SBP | 27440 | 6.42 | pmm |
| HDL-C | 102 | 0.02 | pmm |
| CRP | 939 | 0.22 | pmm |
| Abbreviations: TDI: Townsend Deprivation Index; SBP: Systolic Blood Pressure; HDL-C: High-Density Lipoprotein Cholesterol; CRP: C-Reactive Protein; | | | |

To address missing predictors, we employed the “mice” package in R for imputation, tailoring the method to the data type: (1) Continuous variables were imputed using predictive mean matching; (2) Binary variables via logistic regression; (3) unordered categorical variables via polytomous logistic regression; (4) Ordered categorical variables via proportional odds models. We created five imputed datasets and selected the mode of each variable across these datasets as the final values.

**Table S3** The baseline characteristics of cognitive function stratified by the presence and absence of the condition.

| Characteristic | Total (N = 36831) | Non-cognitive decline  (N = 27634) | Cognitive decline  (N = 9197) | *P*-value |
| --- | --- | --- | --- | --- |
| Age, n(%) | | | | <0.001 |
| ≤65 | 31677 (86.01) | 24547 (88.83) | 7130 (77.53) |  |
| ＞65 | 5154 (13.99) | 3087 (11.17) | 2067 (22.47) |  |
| Sex, n(%) |  |  |  | <0.001 |
| Male | 16954 (46.03) | 13150 (47.59) | 3804 (41.36) |  |
| Female | 19877 (53.97) | 14484 (52.41) | 5393 (58.64) |  |
| Race,n(%) |  |  |  | <0.001 |
| White | 35614 (96.70) | 27043 (97.86) | 8571 (93.19) |  |
| Others | 1217 (3.30) | 591 (2.14) | 626 (6.81) |  |
| Residence, n(%) |  |  |  | <0.001 |
| Urban | 27635 (75.03) | 20385 (73.77) | 7250 (78.83) |  |
| Rural | 9196 (24.97) | 7249 (26.23) | 1947 (21.17) |  |
| Educational level, n(%) | | | | <0.001 |
| College/Above | 11908 (32.33) | 10172 (36.81) | 1736 (18.88) |  |
| Others | 24923 (67.67) | 17462 (63.19) | 7461 (81.12) |  |
| Smoking status, n(%) |  |  |  | <0.001 |
| Current | 3619 (9.83) | 2609 (9.44) | 1010 (10.98) |  |
| Previous | 13142 (35.68) | 9772 (35.36) | 3370 (36.64) |  |
| Never | 20070 (54.49) | 15253 (55.20) | 4817 (52.38) |  |
| Drinking status, n(%) |  |  |  | <0.001 |
| Current | 34183 (92.81) | 25976 (94.00) | 8207 (89.24) |  |
| Previous | 1328 (3.61) | 876 (3.17) | 452 (4.91) |  |
| Never | 1320 (3.58) | 782 (2.83) | 538 (5.85) |  |
| TDI,n(%) |  |  |  | <0.001 |
| Q1 | 9248 (25.11) | 7256 (26.26) | 1992 (21.66) |  |
| Q2 | 9194 (24.96) | 7074 (25.60) | 2120 (23.05) |  |
| Q3 | 9205 (24.99) | 6884 (24.91) | 2321 (25.24) |  |
| Q4 | 9184 (24.94) | 6420 (23.23) | 2764 (30.05) |  |
| History of anxiety, n(%) | | | | <0.001 |
| No | 16379 (44.47) | 12605 (45.61) | 3774 (41.04) |  |
| Yes | 20452 (55.53) | 15029 (54.39) | 5423 (58.96) |  |
| Family history of diabetes, n(%) | | | | 0.432 |
| No | 30506 (82.83) | 22913 (82.92) | 7593 (82.56) |  |
| Yes | 6325 (17.17) | 4721 (17.08) | 1604 (17.44) |  |
| History of stroke, n(%) | | | | <0.001 |
| No | 35699 (96.93) | 26918 (97.41) | 8781 (95.48) |  |
| Yes | 1132 (3.07) | 716 (2.59) | 416 (4.52) |  |
| SBP | 82.00 (75.00, 90.00) | 82.00 (75.00, 90.00) | 82.00 (75.00, 89.00) | 0.016 |
| HDL-C | 1.41 (1.18, 1.68) | 1.40 (1.18, 1.67) | 1.42 (1.18, 1.70) | 0.140 |
| CRP | 1.31 (0.65, 2.67) | 1.26 (0.63, 2.56) | 1.48 (0.72, 3.07) | <0.001 |
| TyG | 8.72 (8.36, 9.12) | 8.71 (8.35, 9.11) | 8.74 (8.39, 9.13) | <0.001 |
| ABSI | 0.08 (0.07, 0.08) | 0.08 (0.07, 0.08) | 0.08 (0.07, 0.08) | <0.001 |
| TyG-WC | 778.65 (678.43, 880.93) | 777.12 (676.59, 880.00) | 783.53 (684.25, 883.10) | <0.001 |
| TyG-BMI | 233.95 (205.25, 267.83) | 232.91 (204.38, 266.93) | 236.96 (207.93, 270.25) | <0.001 |
| TyG-WHtR | 4.60 (4.06, 5.17) | 4.57 (4.04, 5.13) | 4.68 (4.15, 5.25) | <0.001 |
| TyG-ABSI | 0.67 (0.61, 0.72) | 0.66 (0.61, 0.71) | 0.67 (0.62, 0.72) | <0.001 |
| Abbreviations: TDI: Townsend Deprivation Index; SBP: Systolic Blood Pressure; HDL-C: High-Density Lipoprotein Cholesterol; CRP: C-Reactive Protein; TyG: Triglyceride glucose; ABSI: A body shape index; TyG-WC: Triglyceride-glucose index-Waist Circumference; TyG-BMI: Triglyceride-glucose index-Body Mass Index; TyG-WHtR: Triglyceride-glucose index-Waist-to-Height Ratio; TyG-ABSI: Triglyceride Glucose-A Body Shape Index. | | | | |

**Table S4** For cognitive decline, post-hoc tests were conducted to compare pairwise baseline characteristics that showed significant differences in global assessments.

| variable/Group | Q1 vs Q2 | Q1 vs Q3 | Q1 vs Q4 | Q2 vs Q3 | Q2 vs Q4 | Q3 vs Q4 |
| --- | --- | --- | --- | --- | --- | --- |
| Age, n (%) | ** | ** | ** | ** | ** | ** |
| Sex, n (%) | ** | ** | ** | ** | ** | ** |
| Race, n (%) | ns | ns | ** | ns | ** | ** |
| Residence, n (%) | ns | * | ** | ns | ** | ns |
| Educational level, n (%) | ** | * | ** | * | ** | ns |
| Smoking status, n (%) | ** | ** | ** | ** | ** | ** |
| Drinking status, n (%) | ns | ns | ** | ns | * | ns |
| TDI, n (%) | ** | ** | ** | * | ** | ** |
| History of anxiety, n (%) | ** | ** | ** | ** | ** | ** |
| Family history of diabetes, n (%) | * | ** | ** | ns | ** | ** |
| History of stroke, n (%) | ** | ns | ** | ns | ** | ** |
| SBP | *** | *** | *** | *** | *** | *** |
| HDL-C | *** | *** | *** | *** | *** | *** |
| CRP | *** | *** | *** | *** | *** | *** |
| TyG | *** | *** | *** | *** | *** | *** |
| ABSI | *** | *** | *** | *** | *** | *** |
| TyG-WC | *** | *** | *** | *** | *** | *** |
| TyG-BMI | *** | *** | *** | *** | *** | *** |
| TyG-WHtR | *** | *** | *** | *** | *** | *** |
| TyG-ABSI | *** | *** | *** | *** | *** | *** |
| Note: ***p < 0.001, **p < 0.01, *p < 0.05; ns = not significant. All p-values are Holm-corrected. Q 1–4 correspond to tygabsi quartiles (lowest to highest). | | | | | | |

**Table S5** Logistic regression results of standardized continuous TyG, ABSI, and TyG-ABSI associated with cognition decline.

| Classify | Model1 | | Model2 | | Model3 | |
| --- | --- | --- | --- | --- | --- | --- |
|  | OR (95%CI) | *P* | OR (95%CI) | *P* | OR (95%CI) | *P* |
| TyG | 1.06 (1.04~1.09) | <0.001 | 1.08 (1.05~1.10) | <0.001 | 1.06 (1.03~1.09) | <0.001 |
| ABSI | 1.08 (1.06~1.11) | <0.001 | 1.16 (1.12~1.19) | <0.001 | 1.13 (1.10~1.17) | <0.001 |
| TyG-ABSI | 1.09 (1.07~1.12) | <0.001 | 1.15 (1.12~1.18) | <0.001 | 1.13 (1.10~1.16) | <0.001 |
| Abbreviations: TyG: Triglyceride glucose; ABSI: A body shape index; TyG-ABSI: Triglyceride Glucose-A Body Shape Index; OR (95% CI): Odds Ratio (95% Confidence Interval); TDI: Townsend Deprivation Index; CRP: C-Reactive Protein; SBP: Systolic Blood Pressure; HDL-C: High-Density Lipoprotein Cholesterol.  Model1: Crude.  Model2: Adjusted for age, sex, race, and residence.  Model3: Age, sex, race, residence, educational level, smoking status and drinking status, TDI, history of anxiety, history of stroke, family history of diabetes, CRP, SBP, and HDL-C were adjusted for. | | | | | | |

**Table S6** Subgroup analysis for TyG of cognitive decline.

| Variable | Q1 | Q2 | Q3 | Q4 | *P* for interaction |
| --- | --- | --- | --- | --- | --- |
| Age |  |  |  |  | 0.085 |
| ≤65 | Ref. | 1.12(1.04~1.21) | 1.16(1.07~1.25) | 1.14(1.06~1.23) |  |
| ＞65 | Ref. | 0.90(0.75~1.06) | 0.94(0.7~1.11) | 0.98(0.83~1.16) |  |
| Sex |  |  |  |  | <0.001 |
| Male | Ref. | 1.08(0.96~1.21) | 1.03(0.92~1.15) | 0.97(0.87~1.08) |  |
| Female | Ref. | 1.19(1.09~1.30) | 1.37(1.26~1.50) | 1.56(1.42~1.70) |  |
| Race |  |  |  |  | 0.427 |
| White | Ref. | 1.16(1.08~1.24) | 1.20(1.12~1.28) | 1.18(1.10~1.26) |  |
| Others | Ref. | 0.97(0.71~1.34) | 1.23(0.89~1.69) | 1.30(0.96~1.75) |  |
| Residence |  |  |  |  | 0.571 |
| Urban | Ref. | 1.14(1.06~1.23) | 1.21(1.12~1.31) | 1.20(1.11~1.29) |  |
| Rural | Ref. | 1.12(0.97~1.29) | 1.10(0.95~1.27) | 1.10(0.95~1.27) |  |
| Educational level |  |  |  |  | 0.720 |
| College/Above | Ref. | 1.04(0.90~1.19) | 1.10(0.95~1.26) | 1.04(0.90~1.20) |  |
| Others | Ref. | 1.13(1.04~1.22) | 1.12(1.04~1.22) | 1.11(1.03~1.20) |  |
| Smoking status |  |  |  |  | 0.254 |
| Current | Ref. | 1.16(0.93~1.44) | 1.09(0.88~1.35) | 1.06(0.86~1.31) |  |
| Previous | Ref. | 1.10(0.98~1.24) | 1.07(0.95~1.20) | 1.11(0.99~1.24) |  |
| Never | Ref. | 1.14(1.04~1.24) | 1.26(1.15~1.38) | 1.22(1.11~1.34) |  |
| Drinking status |  |  |  |  | 0.542 |
| Current | Ref. | 1.15(1.07~1.23) | 1.18(1.10~1.26) | 1.15(1.07~1.24) |  |
| Previous | Ref. | 1.00(0.70~1.41) | 1.24(0.89~1.73) | 1.26(0.92~1.72) |  |
| Never | Ref. | 0.93(0.67~1.29) | 1.11(0.80~1.54) | 1.22(0.89~1.67) |  |
| TDI |  |  |  |  | 0.433 |
| Q1 | Ref. | 1.03(0.90~1.19) | 1.01(0.87~1.16) | 1.06(0.92~1.22) |  |
| Q2 | Ref. | 1.19(1.03~1.36) | 1.27(1.11~1.46) | 1.26(1.09~1.45) |  |
| Q3 | Ref. | 1.21(1.06~1.39) | 1.22(1.06~1.39) | 1.17(1.02~1.33) |  |
| Q4 | Ref. | 1.10(0.96~1.25) | 1.22(1.07~1.39) | 1.16(1.02~1.32) |  |
| History of anxiety |  |  |  |  | 0.527 |
| Yes | Ref. | 1.15(1.06~1.26) | 1.23(1.13~1.34) | 1.23(1.13~1.35) |  |
| No | Ref. | 1.12(1.00~1.24) | 1.13(1.02~1.26) | 1.13(1.02~1.25) |  |
| History of stroke |  |  |  |  | 0.793 |
| Yes | Ref. | 0.98(0.67~1.41) | 1.00(0.70~1.42) | 1.01(0.72~1.43) |  |
| No | Ref. | 1.14(1.06~1.22) | 1.18(1.11~1.27) | 1.17(1.10~1.26) |  |
| Family history of diabetes | | | | | 0.598 |
| Yes | Ref. | 1.15(0.97~1.36) | 1.09(0.92~1.29) | 1.16(0.99~1.37) |  |
| No | Ref. | 1.13(1.05~1.22) | 1.20(1.12~1.29) | 1.18(1.09~1.27) |  |
| Abbreviations: TDI: Townsend Deprivation Index. | | | | | |

**Table S7** Subgroup analysis for ABSI of cognitive decline.

| Variable | Q1 | Q2 | Q3 | Q4 | *P* for interaction |
| --- | --- | --- | --- | --- | --- |
| Age |  |  |  |  | 0.068 |
| ≤65 | Ref. | 1.03(0.96~1.11) | 0.99(0.92~1.07) | 1.15(1.07~1.24) |  |
| ＞65 | Ref. | 0.99(0.83~1.19) | 1.02(0.86~1.21) | 0.97(0.82~1.14) |  |
| Sex |  |  |  |  | 0.098 |
| Male | Ref. | 1.02(0.83~1.26) | 1.27(1.04~1.55) | 1.60(1.32~1.95) |  |
| Female | Ref. | 1.22(1.13~1.31) | 1.34(1.23~1.47) | 1.59(1.43~1.77) |  |
| Race |  |  |  |  | 0.010 |
| White | Ref. | 1.03(0.96~1.10) | 1.03(0.96~1.10) | 1.15(1.08~1.24) |  |
| Others | Ref. | 1.37(0.96~1.95) | 1.43(1.01~2.04) | 2.05(1.47~2.87) |  |
| Residence |  |  |  |  | 0.839 |
| Urban | Ref. | 1.05(0.97~1.13) | 1.05(0.98~1.14) | 1.22(1.13~1.32) |  |
| Rural | Ref. | 1.03(0.90~1.19) | 1.01(0.88~1.16) | 1.14(0.99~1.31) |  |
| Educational level |  |  |  |  | 0.059 |
| College/Above | Ref. | 0.88(0.76~1.02) | 0.93(0.81~1.07) | 1.09(0.95~1.25) |  |
| Others | Ref. | 1.10(1.02~1.19) | 1.09(1.01~1.17) | 1.26(1.17~1.36) |  |
| Smoking status |  |  |  |  | 0.475 |
| Current | Ref. | 1.05(0.84~1.33) | 1.12(0.89~1.39) | 1.38(1.11~1.70) |  |
| Previous | Ref. | 1.02(0.90~1.14) | 1.01(0.90~1.13) | 1.10(0.98~1.23) |  |
| Never | Ref. | 1.06(0.97~1.16) | 1.04(0.95~1.14) | 1.24(1.13~1.36) |  |
| Drinking status |  |  |  |  | 0.392 |
| Current | Ref. | 1.04(0.97~1.12) | 1.05(0.98~1.13) | 1.21(1.13~1.30) |  |
| Previous | Ref. | 1.41(1.01~1.97) | 1.22(0.86~1.72) | 1.68(1.22~2.33) |  |
| Never | Ref. | 1.11(0.82~1.49) | 1.28(0.94~1.74) | 1.23(0.91~1.67) |  |
| TDI |  |  |  |  | 0.034 |
| Q1 | Ref. | 1.00(0.87~1.15) | 0.89(0.78~1.02) | 1.05(0.92~1.21) |  |
| Q2 | Ref. | 1.11(0.97~1.27) | 0.97(0.84~1.11) | 1.19(1.04~1.36) |  |
| Q3 | Ref. | 0.96(0.84~1.10) | 1.11(0.97~1.27) | 1.19(1.05~1.36) |  |
| Q4 | Ref. | 1.08(0.95~1.24) | 1.17(1.03~1.34) | 1.30(1.14~1.47) |  |
| History of anxiety |  |  |  |  | 0.049 |
| Yes | Ref. | 1.11(1.02~1.21) | 1.12(1.03~1.23) | 1.22(1.12~1.33) |  |
| No | Ref. | 0.98(0.88~1.10) | 0.99(0.89~1.10) | 1.25(1.13~1.39) |  |
| History of stroke |  |  |  |  | 0.282 |
| Yes | Ref. | 0.87(0.58~1.32) | 1.24(0.84~1.85) | 1.23(0.85~1.77) |  |
| No | Ref. | 1.05(0.98~1.13) | 1.04(0.97~1.11) | 1.19(1.12~1.28) |  |
| Family history of diabetes | | | | | 0.309 |
| Yes | Ref. | 1.10(0.93~1.29) | 0.99(0.84~1.17) | 1.31(1.11~1.54) |  |
| No | Ref. | 1.04(0.97~1.12) | 1.06(0.99~1.15) | 1.20(1.12~1.29) |  |
| Abbreviations: TDI: Townsend Deprivation Index. | | | | | |

**Table S8** Subgroup analysis for TyG-ABSI of cognitive decline.

| Variable | Q1 | Q2 | Q3 | Q4 | *P* for interaction |
| --- | --- | --- | --- | --- | --- |
| Age | | | | | 0.251 |
| ≤65 | Ref. | 1.07(0.99~1.15) | 1.10(1.02~1.19) | 1.16(1.08~1.25) |  |
| ＞65 | Ref. | 0.96(0.80~1.15) | 0.92(0.77~1.10) | 0.98(0.82~1.17) |  |
| Sex |  |  |  |  | 0.001 |
| Male | Ref. | 0.95(0.80~1.12) | 1.13(0.97~1.32) | 1.24(1.07~1.45) |  |
| Female | Ref. | 1.28(1.18~1.38) | 1.40(1.29~1.53) | 1.79(1.62~1.98) |  |
| Race |  |  |  |  | 0.012 |
| White | Ref. | 1.11(1.03~1.19) | 1.15(1.07~1.23) | 1.19(1.11~1.28) |  |
| Others | Ref. | 1.23(0.88~1.72) | 1.34(0.96~1.88) | 1.95(1.43~2.66) |  |
| Residence |  |  |  |  | 0.310 |
| Urban | Ref. | 1.15(1.06~1.24) | 1.16(1.08~1.26) | 1.27(1.18~1.37) |  |
| Rural | Ref. | 1.00(0.87~1.15) | 1.08(0.94~1.25) | 1.12(0.97~1.29) |  |
| Educational level | | | | | 0.272 |
| College/Above | Ref. | 1.01(0.88~1.17) | 1.00(0.87~1.16) | 1.07(0.93~1.23) |  |
| Others | Ref. | 1.11(1.03~1.20) | 1.14(1.06~1.24) | 1.24(1.15~1.34) |  |
| Smoking status |  |  |  |  | 0.248 |
| Current | Ref. | 1.11(0.88~1.40) | 1.07(0.85~1.34) | 1.34(1.08~1.66) |  |
| Previous | Ref. | 1.07(0.95~1.21) | 1.04(0.92~1.17) | 1.14(1.02~1.28) |  |
| Never | Ref. | 1.12(1.02~1.22) | 1.22(1.11~1.33) | 1.24(1.13~1.36) |  |
| Drinking status |  |  |  |  | 0.532 |
| Current | Ref. | 1.10(1.02~1.18) | 1.13(1.06~1.22) | 1.22(1.14~1.31) |  |
| Previous | Ref. | 1.27(0.90~1.80) | 1.25(0.89~1.76) | 1.57(1.13~2.18) |  |
| Never | Ref. | 1.28(0.94~1.73) | 1.49(1.10~2.04) | 1.38(1.01~1.89) |  |
| TDI |  |  |  |  | 0.120 |
| Q1 | Ref. | 1.09(0.96~1.25) | 0.97(0.85~1.12) | 1.08(0.94~1.25) |  |
| Q2 | Ref. | 1.07(0.93~1.22) | 1.25(1.09~1.44) | 1.22(1.06~1.40) |  |
| Q3 | Ref. | 1.16(1.01~1.33) | 1.19(1.04~1.36) | 1.22(1.07~1.39) |  |
| Q4 | Ref. | 1.10(0.96~1.25) | 1.13(0.99~1.29) | 1.31(1.15~1.49) |  |
| History of anxiety |  |  |  |  | 0.268 |
| Yes | Ref. | 1.18(1.08~1.29) | 1.21(1.11~1.32) | 1.30(1.19~1.42) |  |
| No | Ref. | 1.03(0.93~1.15) | 1.10(0.99~1.22) | 1.22(1.10~1.36) |  |
| History of stroke |  |  |  |  | 0.690 |
| Yes | Ref. | 0.98(0.65~1.48) | 1.25(0.84~1.86) | 1.21(0.83~1.75) |  |
| No | Ref. | 1.11(1.04~1.19) | 1.14(1.06~1.22) | 1.23(1.14~1.31) |  |
| Family history of diabetes | | | | | 0.885 |
| Yes | Ref. | 1.04(0.88~1.24) | 1.12(0.95~1.32) | 1.20(1.02~1.41) |  |
| No | Ref. | 1.13(1.05~1.21) | 1.16(1.07~1.24) | 1.25(1.16~1.35) |  |
| Abbreviations: TDI: Townsend Deprivation Index. | | | | | |

**Table S9** Sensitivity analysis for logistic regression analyses of TyG,ABSI and TyG-ABSI indices with cognitive decline post-imputation.

| Variables | Model1 | | Model2 | | Model3 | |
| --- | --- | --- | --- | --- | --- | --- |
|  | OR (95%CI) | *P* | OR (95%CI) | *P* | OR (95%CI) | *P* |
| TyG |  |  |  |  |  |  |
| Q1 | Ref. |  | Ref. |  | Ref. |  |
| Q2 | 1.13 (1.06 ~ 1.21) | <0.001 | 1.14 (1.06 ~ 1.21) | <0.001 | 1.11 (1.04 ~ 1.19) | 0.002 |
| Q3 | 1.19 (1.11 ~ 1.27) | <0.001 | 1.21 (1.13 ~ 1.29) | <0.001 | 1.16 (1.08 ~ 1.25) | <0.001 |
| Q4 | 1.17 (1.09 ~ 1.25) | <0.001 | 1.20 (1.13 ~ 1.29) | <0.001 | 1.15 (1.06 ~ 1.24) | <0.001 |
| ABSI |  |  |  |  |  |  |
| Q1 | Ref. |  | Ref. |  | Ref. |  |
| Q2 | 1.04 (0.98 ~ 1.11) | 0.207 | 1.11 (1.04 ~ 1.19) | 0.002 | 1.09 (1.02 ~ 1.17) | 0.011 |
| Q3 | 1.04 (0.98 ~ 1.11) | 0.201 | 1.22 (1.14 ~ 1.31) | <0.001 | 1.18 (1.10 ~ 1.27) | <0.001 |
| Q4 | 1.23 (1.16 ~ 1.32) | <0.001 | 1.45 (1.35 ~ 1.57) | <0.001 | 1.37 (1.27 ~ 1.48) | <0.001 |
| TyG-ABSI |  |  |  |  |  |  |
| Q1 | Ref. |  | Ref. |  | Ref. |  |
| Q2 | 1.10 (1.03 ~ 1.17) | 0.005 | 1.14 (1.07 ~ 1.22) | <0.001 | 1.12 (1.04 ~ 1.20) | 0.002 |
| Q3 | 1.15 (1.08 ~ 1.23) | <0.001 | 1.28 (1.20 ~ 1.38) | <0.001 | 1.24 (1.15 ~ 1.34) | <0.001 |
| Q4 | 1.24 (1.17 ~ 1.33) | <0.001 | 1.41 (1.31 ~ 1.52) | <0.001 | 1.35 (1.25 ~ 1.47) | <0.001 |
| Abbreviations: TyG: Triglyceride glucose; ABSI: A body shape index; TyG-ABSI: Triglyceride Glucose-A Body Shape Index; OR (95% CI): Odds Ratio (95% Confidence Interval); TDI: Townsend Deprivation Index; CRP: C-Reactive Protein; SBP: Systolic Blood Pressure; HDL-C: High-Density Lipoprotein Cholesterol.  Model1: Crude.  Model2: Adjusted for age, sex, race, and residence.  Model3: Age, sex, race, residence, educational level, smoking status and drinking status, TDI, history of anxiety, history of stroke, family history of diabetes, CRP, SBP, and HDL-C were adjusted for. | | | | | | |

**Table S10** Sensitivity analysis for performing logistic regression analyses of TyG,ABSI and TyG-ABSI indices with cognitive decline after removing outliers.

| Variables | Model1 | | Model2 | | Model3 | |
| --- | --- | --- | --- | --- | --- | --- |
|  | OR (95%CI) | *P* | OR (95%CI) | *P* | OR (95%CI) | *P* |
| **TyG** |  |  |  |  |  |  |
| Q1 | Ref. |  | Ref. |  | Ref. |  |
| Q2 | 1.14 (1.06 ~ 1.22) | <0.001 | 1.14 (1.06 ~ 1.22) | <0.001 | 1.11 (1.03 ~ 1.20) | 0.006 |
| Q3 | 1.18 (1.09 ~ 1.26) | <0.001 | 1.20 (1.11 ~ 1.29) | <0.001 | 1.15 (1.07 ~ 1.25) | <0.001 |
| Q4 | 1.19 (1.10 ~ 1.28) | <0.001 | 1.23 (1.14 ~ 1.33) | <0.001 | 1.17 (1.08 ~ 1.28) | <0.001 |
| **ABSI** |  |  |  |  |  |  |
| Q1 | Ref. |  | Ref. |  | Ref. |  |
| Q2 | 1.05 (0.97 ~ 1.13) | 0.206 | 1.13 (1.05 ~ 1.22) | 0.002 | 1.11 (1.03 ~ 1.20) | 0.007 |
| Q3 | 1.04 (0.96 ~ 1.11) | 0.343 | 1.23 (1.14 ~ 1.34) | <0.001 | 1.20 (1.10 ~ 1.30) | <0.001 |
| Q4 | 1.20 (1.11 ~ 1.29) | <0.001 | 1.42 (1.30 ~ 1.55) | <0.001 | 1.35 (1.23 ~ 1.48) | <0.001 |
| **TyG-ABSI** |  |  |  |  |  |  |
| Q1 | Ref. |  | Ref. |  | Ref. |  |
| Q2 | 1.10 (1.02 ~ 1.18) | 0.013 | 1.15 (1.07 ~ 1.24) | <0.001 | 1.13 (1.04 ~ 1.22) | 0.003 |
| Q3 | 1.15 (1.07 ~ 1.23) | <0.001 | 1.30 (1.21 ~ 1.41) | <0.001 | 1.26 (1.16 ~ 1.37) | <0.001 |
| Q4 | 1.22 (1.13 ~ 1.31) | <0.001 | 1.41 (1.29 ~ 1.53) | <0.001 | 1.35 (1.23 ~ 1.48) | <0.001 |
| Abbreviations: TyG: Triglyceride glucose; ABSI: A body shape index; TyG-ABSI: Triglyceride Glucose-A Body Shape Index; OR (95% CI): Odds Ratio (95% Confidence Interval); TDI: Townsend Deprivation Index; CRP: C-Reactive Protein; SBP: Systolic Blood Pressure; HDL-C: High-Density Lipoprotein Cholesterol.  Model1: Crude.  Model2: Adjusted for age, sex, race, and residence.  Model3: Age, sex, race, residence, educational level, smoking status and drinking status, TDI, history of anxiety, history of stroke, family history of diabetes, CRP, SBP, and HDL-C were adjusted for. | | | | | | |

**Table S11** Sensitivity analysis for performing logistic regression analyses of TyG, ABSI and TyG-ABSI indices with cognitive decline after adding sleep duration to the full adjustment model.

| Variables | Model1 | | Model2 | | Model3 | |
| --- | --- | --- | --- | --- | --- | --- |
|  | OR (95%CI) | *P* | OR (95%CI) | *P* | OR (95%CI) | *P* |
| TyG |  |  |  |  |  |  |
| Q1 | Ref. |  | Ref. |  | Ref. |  |
| Q2 | 1.14 (1.06 ~ 1.22) | <0.001 | 1.13 (1.05 ~ 1.21) | <0.001 | 1.11 (1.04 ~ 1.19) | 0.004 |
| Q3 | 1.18 (1.10 ~ 1.26) | <0.001 | 1.19 (1.11 ~ 1.28) | <0.001 | 1.15 (1.07 ~ 1.24) | <0.001 |
| Q4 | 1.17 (1.10 ~ 1.26) | <0.001 | 1.21 (1.13 ~ 1.30) | <0.001 | 1.15 (1.06 ~ 1.24) | <0.001 |
| ABSI |  |  |  |  |  |  |
| Q1 | Ref. |  | Ref. |  | Ref. |  |
| Q2 | 1.05 (0.98 ~ 1.12) | 0.170 | 1.12 (1.04 ~ 1.20) | 0.002 | 1.10 (1.02 ~ 1.18) | 0.010 |
| Q3 | 1.05 (0.98 ~ 1.12) | 0.143 | 1.23 (1.15 ~ 1.33) | <0.001 | 1.20 (1.11 ~ 1.29) | <0.001 |
| Q4 | 1.22 (1.14 ~ 1.30) | <0.001 | 1.44 (1.33 ~ 1.56) | <0.001 | 1.35 (1.24 ~ 1.47) | <0.001 |
| TyG-ABSI |  |  |  |  |  |  |
| Q1 | Ref. |  | Ref. |  | Ref. |  |
| Q2 | 1.11 (1.04 ~ 1.19) | 0.002 | 1.16 (1.08 ~ 1.24) | <0.001 | 1.13 (1.05 ~ 1.22) | <0.001 |
| Q3 | 1.15 (1.07 ~ 1.23) | <0.001 | 1.29 (1.20 ~ 1.39) | <0.001 | 1.24 (1.15 ~ 1.34) | <0.001 |
| Q4 | 1.24 (1.16 ~ 1.33) | <0.001 | 1.42 (1.31 ~ 1.53) | <0.001 | 1.35 (1.24 ~ 1.47) | <0.001 |
| Abbreviations: TyG: Triglyceride glucose; ABSI: A body shape index; TyG-ABSI: Triglyceride Glucose-A Body Shape Index; OR (95% CI): Odds Ratio (95% Confidence Interval); TDI: Townsend Deprivation Index; CRP: C-Reactive Protein; SBP: Systolic Blood Pressure; HDL-C: High-Density Lipoprotein Cholesterol.  Model1: Crude.  Model2: Adjusted for age, sex, race, and residence.  Model3: Age, sex, race, residence, educational level, smoking status and drinking status, TDI, history of anxiety, history of stroke, family history of diabetes, CRP, SBP, and HDL-C were adjusted for. | | | | | | |

**Table S12** Sensitivity analysis for Logistic regression analyses of TyG, ABSI and TyG-ABSI indices with cognitive decline was performed without adjusting for HDL-C and CRP in the fully adjusted model.

| **Types** | **Q1** | **Q2** | | **Q3** | | **Q4** | |
| --- | --- | --- | --- | --- | --- | --- | --- |
|  |  | **OR (95%CI)** | **P value** | **OR (95%CI)** | **P value** | **OR (95%CI)** | **P value** |
| **TyG** | Ref | 1.09(1.02,1.17) | 0.014 | 1.12(1.04,1.20) | 0.003 | 1.10(1.02,1.18) | 0.012 |
| **ABSI** | Ref | 1.09(1.02,1.17) | 0.015 | 1.19(1.10,1.28) | <0.001 | 1.34(1.24,1.46) | <0.001 |
| **TyG-ABSI** | Ref | 1.11(1.03,1.19) | 0.005 | 1.19(1.11,1.29) | <0.001 | 1.28(1.18,1.39) | <0.001 |
| Abbreviations: TyG: Triglyceride glucose; ABSI: A body shape index; TyG-ABSI: Triglyceride Glucose-A Body Shape Index; OR (95% CI): Odds Ratio (95% Confidence Interval); TDI: Townsend Deprivation Index; SBP: Systolic Blood Pressure.  Model1: Crude.  Model2: Adjusted for age, sex, race, and residence.  Model3: Age, sex, race, residence, educational level, smoking status and drinking status, TDI, history of anxiety, history of stroke, family history of diabetes, SBP were adjusted for. | | | | | | | |

**Table S13** Sensitivity analysis for Logistic regression analyses of TyG, ABSI and TyG-ABSI indices with cognitive decline was performed additionally adjusting for glycated haemoglobin (HbA1c) in the fully adjusted model.

| **Types** | **Q1** | **Q2** | | **Q3** | | **Q4** | |
| --- | --- | --- | --- | --- | --- | --- | --- |
|  |  | **OR (95%CI)** | **P value** | **OR (95%CI)** | **P value** | **OR (95%CI)** | **P value** |
| **TyG** | Ref | 1.10(1.02,1.18) | 0.010 | 1.13(1.05,1.22) | 0.001 | 1.09(1.01,1.18) | 0.043 |
| **ABSI** | Ref | 1.09(1.01,1.17) | 0.021 | 1.18(1.09,1.27) | <0.001 | 1.29(1.19,1.41) | <0.001 |
| **TyG-ABSI** | Ref | 1.12(1.04,1.21) | 0.002 | 1.22(1.13,1.32) | <0.001 | 1.28(1.17,1.40) | <0.001 |
| Abbreviations: TyG: Triglyceride glucose; ABSI: A body shape index; TyG-ABSI: Triglyceride Glucose-A Body Shape Index; OR (95% CI): Odds Ratio (95% Confidence Interval); TDI: Townsend Deprivation Index; SBP: Systolic Blood Pressure; HDL-C: High-Density Lipoprotein Cholesterol; HbA1c: glycated haemoglobin.  Model1: Crude.  Model2: Adjusted for age, sex, race, and residence.  Model3: Age, sex, race, residence, educational level, smoking status and drinking status, TDI, history of anxiety, history of stroke, family history of diabetes, SBP , CRP, HDL, and **HbA1c** were adjusted for. | | | | | | | |

**Table S14** Sensitivity analysis for TyG, ABSI, and TyG-ABSI indices with cognitive decline (bottom 20%, bottom 30%, and 1 SD below mean).

| **Types** | **Q1** | **Q2** | | **Q3** | | **Q4** | |
| --- | --- | --- | --- | --- | --- | --- | --- |
|  |  | **OR (95%CI)** | **P value** | **OR (95%CI)** | **P value** | **OR (95%CI)** | **P value** |
| **Bottom 20%** |  |  |  |  |  |  |  |
| TyG | Ref | 1.13(1.05,1.23) | 0.001 | 1.17(1.08,1.26) | <0.001 | 1.17(1.07,1.27) | <0.001 |
| ABSI | Ref | 1.09(1.01,1.17) | 0.038 | 1.20(1.11,1.31) | <0.001 | 1.38(1.26,1.50) | <0.001 |
| TyG-ABSI | Ref | 1.13(1.04,1.22) | 0.003 | 1.24(1.14,1.35) | <0.001 | 1.36(1.24,1.49) | <0.001 |
| **Bottom 30%** |  |  |  |  |  |  |  |
| TyG | Ref | 1.10(1.03,1.18) | 0.005 | 1.15(1.07,1.23) | <0.001 | 1.15(1.07,1.24) | <0.001 |
| ABSI | Ref | 1.06(0.99,1.14) | 0.077 | 1.17(1.09,1.26) | <0.001 | 1.33(1.23,1.44) | <0.001 |
| TyG-ABSI | Ref | 1.11(1.04,1.19) | 0.002 | 1.22(1.14,1.32) | <0.001 | 1.33(1.23,1.44) | <0.001 |
| **Below 1 SD of mean** |  |  |  |  |  |  |  |
| TyG | Ref | 1.08(0.99,1.18) | 0.075 | 1.17(1.07,1.28) | <0.001 | 1.16(1.06,1.28) | <0.001 |
| ABSI | Ref | 1.10(1.01,1.20) | 0.028 | 1.22(1.11,1.34) | <0.001 | 1.38(1.25,1.52) | <0.001 |
| TyG-ABSI | Ref | 1.11(1.02,1.21) | 0.022 | 1.20(1.09,1.32) | <0.001 | 1.36(1.23,1.51) | <0.001 |
| Abbreviations: TyG: Triglyceride glucose; ABSI: A body shape index; TyG-ABSI: Triglyceride Glucose-A Body Shape Index; OR (95% CI): Odds Ratio (95% Confidence Interval); TDI: Townsend Deprivation Index; CRP: C-Reactive Protein; SBP: Systolic Blood Pressure; HDL-C: High-Density Lipoprotein Cholesterol. Model1: Crude. Model2: Adjusted for age, sex, race, and residence. Model3: Age, sex, race, residence, educational level, smoking status and drinking status, TDI, history of anxiety, history of stroke, family history of diabetes, CRP, SBP, and HDL-C were adjusted for. | | | | | | | |

**Table S15** The baseline characteristics of all-cause dementia stratified by disease presence and absence.

| Characteristic | Total(N = 370,744) | Non-ACD(N = 363,806) | ACD(N = 6,938) | *P*-value |
| --- | --- | --- | --- | --- |
| Age, n(%) | | | | <0.001 |
| ≤65 | 316,571 (85.39) | 313,005 (86.04) | 3,566 (51.40) |  |
| ＞65 | 54,173 (14.61) | 50,801 (13.96) | 3,372 (48.60) |  |
| Sex, n(%) |  |  |  | <0.001 |
| Male | 170,349 (45.95) | 166,741 (45.83) | 3,608 (52.00) |  |
| Female | 200,395 (54.05) | 197,065 (54.17) | 3,330 (48.00) |  |
| Race,n(%) |  |  |  | <0.001 |
| White | 352,090 (94.97) | 345,404 (94.94) | 6,686 (96.37) |  |
| Others | 18,654 (5.03) | 18,402 (5.06) | 252 (3.63) |  |
| Residence, n(%) |  |  |  | <0.001 |
| Urban | 318,077 (85.79) | 312,010 (85.76) | 6,067 (87.45) |  |
| Rural | 52,667 (14.21) | 51,796 (14.24) | 871 (12.55) |  |
| Educational level, n(%) | | | | <0.001 |
| College/Above | 121,246 (32.70) | 119,781 (32.92) | 1,465 (21.12) |  |
| Others | 249,498 (67.30) | 244,025 (67.08) | 5,473 (78.88) |  |
| Smoking status, n(%) |  |  |  | <0.001 |
| Current | 38,178 (10.30) | 37,475 (10.30) | 703 (10.13) |  |
| Previous | 129,424 (34.91) | 126,435 (34.75) | 2,989 (43.08) |  |
| Never | 203,142 (54.79) | 199,896 (54.95) | 3,246 (46.79) |  |
| Drinking status, n(%) |  |  |  | <0.001 |
| Current | 342,037 (92.26) | 336,006 (92.36) | 6,031 (86.93) |  |
| Previous | 13,061 (3.52) | 12,612 (3.47) | 449 (6.47) |  |
| Never | 15,646 (4.22) | 15,188 (4.17) | 458 (6.60) |  |
| TDI,n(%) |  |  |  | <0.001 |
| Q1 | 93,125 (25.12) | 91,508 (25.15) | 1,617 (23.31) |  |
| Q2 | 92,549 (24.96) | 90,892 (24.98) | 1,657 (23.88) |  |
| Q3 | 92,530 (24.96) | 90,826 (24.97) | 1,704 (24.56) |  |
| Q4 | 92,540 (24.96) | 90,580 (24.90) | 1,960 (28.25) |  |
| History of anxiety, n(%) | | | | <0.001 |
| No | 162,265 (43.77) | 159,421 (43.82) | 2,844 (40.99) |  |
| Yes | 208,479 (56.23) | 204,385 (56.18) | 4,094 (59.01) |  |
| Family history of diabetes, n(%) | | | | <0.001 |
| No | 306,043 (82.55) | 300,117 (82.49) | 5,926 (85.41) |  |
| Yes | 64,701 (17.45) | 63,689 (17.51) | 1,012 (14.59) |  |
| History of stroke, n(%) | | | | <0.001 |
| No | 357,843 (96.52) | 351,954 (96.74) | 5,889 (84.88) |  |
| Yes | 12,901 (3.48) | 11,852 (3.26) | 1,049 (15.12) |  |
| SBP | 82 (75.00,89.00) | 82 (75.00,89.00) | 82 (74.00,89.00) | 0.004 |
| HDL-C | 1.40 (1.17,1.68) | 1.40 (1.17,1.68) | 1.37 (1.14,1.66) | <0.001 |
| CRP | 1.32 (0.66,2.75) | 1.32 (0.66,2.74) | 1.43 (0.68,3.02) | <0.001 |
| TyG | 8.68 (8.31,9.07) | 8.68 (8.31,9.07) | 8.75 (8.39,9.15) | <0.001 |
| ABSI | 0.08 (0.07,0.08) | 0.08 (0.07,0.08) | 0.08 (0.07,0.08) | <0.001 |
| TyG-WC | 781.72 (680.71,884.89) | 781.14 (680.14,884.32) | 811.24 (713.29,912.66) | <0.001 |
| TyG-BMI | 233.08 (204.50,267.04) | 232.98 (204.40,266.95) | 238.42 (209.57,271.73) | <0.001 |
| TyG-WHtR | 4.63 (4.08,5.20) | 4.62 (4.08,5.20) | 4.82 (4.30,5.43) | <0.001 |
| TyG-ABSI | 0.67 (0.62,0.72) | 0.67 (0.62,0.72) | 0.69 (0.64,0.74) | <0.001 |
| Abbreviations: TDI: Townsend Deprivation Index; SBP: Systolic Blood Pressure; HDL-C: High-Density Lipoprotein Cholesterol; CRP: C-Reactive Protein; TyG: Triglyceride glucose; ABSI: A body shape index; TyG-WC: Triglyceride-glucose index-Waist Circumference; TyG-BMI: Triglyceride-glucose index-Body Mass Index; TyG-WHtR: Triglyceride-glucose index-Waist-to-Height Ratio; TyG-ABSI: Triglyceride Glucose-A Body Shape Index. | | | | |

**Table S16** The baseline characteristics of Alzheimer's dementia stratified by disease presence and absence.

| Characteristic | Totall(N = 370,744) | Non-AD(N = 367,678) | AD(N = 3,066) | *P*-value |
| --- | --- | --- | --- | --- |
| Age, n(%) | | | | <0.001 |
| ≤65 | 316,571 (85.39) | 315,083 (85.70) | 1,488 (48.53) |  |
| ＞65 | 54,173 (14.61) | 52,595 (14.30) | 1,578 (51.47) |  |
| Sex, n(%) |  |  |  | 0.200 |
| Male | 170,349 (45.95) | 168,905 (45.94) | 1,444 (47.10) |  |
| Female | 200,395 (54.05) | 198,773 (54.06) | 1,622 (52.90) |  |
| Race,n(%) |  |  |  | <0.001 |
| White | 352,090 (94.97) | 349,127 (94.95) | 2,963 (96.64) |  |
| Others | 18,654 (5.03) | 18,551 (5.05) | 103 (3.36) |  |
| Residence, n(%) |  |  |  | 0.081 |
| Urban | 318,077 (85.79) | 315,413 (85.79) | 2,664 (86.89) |  |
| Rural | 52,667 (14.21) | 52,265 (14.21) | 402 (13.11) |  |
| Educational level, n(%) | | | | <0.001 |
| College/Above | 121,246 (32.70) | 120,624 (32.81) | 622 (20.29) |  |
| Others | 249,498 (67.30) | 247,054 (67.19) | 2,444 (79.71) |  |
| Smoking status, n(%) | | | | <0.001 |
| Current | 38,178 (10.30) | 37,905 (10.31) | 273 (8.90) |  |
| Previous | 129,424 (34.91) | 128,120 (34.85) | 1,304 (42.53) |  |
| Never | 203,142 (54.79) | 201,653 (54.85) | 1,489 (48.56) |  |
| Drinking status, n(%) | | | | <0.001 |
| Current | 342,037 (92.26) | 339,361 (92.30) | 2,676 (87.28) |  |
| Previous | 13,061 (3.52) | 12,883 (3.50) | 178 (5.81) |  |
| Never | 15,646 (4.22) | 15,434 (4.20) | 212 (6.91) |  |
| TDI,n(%) |  |  |  | 0.130 |
| Q1 | 93,125 (25.12) | 92,365 (25.12) | 760 (24.79) |  |
| Q2 | 92,549 (24.96) | 91,825 (24.97) | 724 (23.61) |  |
| Q3 | 92,530 (24.96) | 91,763 (24.96) | 767 (25.02) |  |
| Q4 | 92,540 (24.96) | 91,725 (24.95) | 815 (26.58) |  |
| History of anxiety, n(%) | | | | 0.008 |
| No | 162,265 (43.77) | 160,996 (43.79) | 1,269 (41.39) |  |
| Yes | 208,479 (56.23) | 206,682 (56.21) | 1,797 (58.61) |  |
| Family history of diabetes, n(%) | | | | <0.001 |
| No | 306,043 (82.55) | 303,397 (82.52) | 2,646 (86.30) |  |
| Yes | 64,701 (17.45) | 64,281 (17.48) | 420 (13.70) |  |
| History of stroke, n(%) | | | | <0.001 |
| No | 357,843 (96.52) | 355,070 (96.57) | 2,773 (90.44) |  |
| Yes | 12,901 (3.48) | 12,608 (3.43) | 293 (9.56) |  |
| SBP | 82 (75.00,89.00) | 82 (75.00,89.00) | 81 (75.00,88.00) | 0.016 |
| HDL-C | 1.40 (1.17,1.68) | 1.40 (1.17,1.68) | 1.40 (1.16,1.71) | 0.900 |
| CRP | 1.32 (0.66,2.75) | 1.32 (0.66,2.75) | 1.28 (0.63,2.84) | 0.300 |
| TyG | 8.68 (8.31,9.07) | 8.68 (8.31,9.07) | 8.73 (8.39,9.11) | <0.001 |
| ABSI | 0.08 (0.07,0.08) | 0.08 (0.07,0.08) | 0.08 (0.07,0.08) | <0.001 |
| TyG-WC | 781.72 (680.71,884.89) | 781.59 (680.58,884.83) | 795.84 (697.87,894.22) | <0.001 |
| TyG-BMI | 233.08 (204.50,267.04) | 233.07 (204.47,267.06) | 234.44 (207.88,265.85) | 0.040 |
| TyG-WHtR | 4.63 (4.08,5.20) | 4.62 (4.08,5.20) | 4.75 (4.23,5.33) | <0.001 |
| TyG-ABSI | 0.67 (0.62,0.72) | 0.67 (0.62,0.72) | 0.68 (0.63,0.73) | <0.001 |
| Abbreviations: TDI: Townsend Deprivation Index; SBP: Systolic Blood Pressure; HDL-C: High-Density Lipoprotein Cholesterol; CRP: C-Reactive Protein; TyG: Triglyceride glucose; ABSI: A body shape index; TyG-WC: Triglyceride-glucose index-Waist Circumference; TyG-BMI: Triglyceride-glucose index-Body Mass Index; TyG-WHtR: Triglyceride-glucose index-Waist-to-Height Ratio; TyG-ABSI: Triglyceride Glucose-A Body Shape Index. | | | | |

**Table S17** The baseline characteristics of vascular dementia stratified by disease presence and absence.

| Characteristic | Totall(N = 370,744) | Non-VD(N = 369,199) | VD(N = 1,545) | *P*-value |
| --- | --- | --- | --- | --- |
| Age, n(%) | | | | <0.001 |
| ≤65 | 316,571 (85.39) | 315,839 (85.55) | 732 (47.38) |  |
| ＞65 | 54,173 (14.61) | 53,360 (14.45) | 813 (52.62) |  |
| Sex, n(%) |  |  |  | <0.001 |
| Male | 170,349 (45.95) | 169,439 (45.89) | 910 (58.90) |  |
| Female | 200,395 (54.05) | 199,760 (54.11) | 635 (41.10) |  |
| Race,n(%) |  |  |  | 0.016 |
| White | 352,090 (94.97) | 350,602 (94.96) | 1,488 (96.31) |  |
| Others | 18,654 (5.03) | 18,597 (5.04) | 57 (3.69) |  |
| Residence, n(%) |  |  |  | <0.001 |
| Urban | 318,077 (85.79) | 316,694 (85.78) | 1,383 (89.51) |  |
| Rural | 52,667 (14.21) | 52,505 (14.22) | 162 (10.49) |  |
| Educational level, n(%) | | | | <0.001 |
| College/Above | 121,246 (32.70) | 120,979 (32.77) | 267 (17.28) |  |
| Others | 249,498 (67.30) | 248,220 (67.23) | 1,278 (82.72) |  |
| Smoking status, n(%) | | | | <0.001 |
| Current | 38,178 (10.30) | 37,999 (10.29) | 179 (11.59) |  |
| Previous | 129,424 (34.91) | 128,729 (34.87) | 695 (44.98) |  |
| Never | 203,142 (54.79) | 202,471 (54.84) | 671 (43.43) |  |
| Drinking status, n(%) | | | | <0.001 |
| Current | 342,037 (92.26) | 340,705 (92.28) | 1,332 (86.21) |  |
| Previous | 13,061 (3.52) | 12,951 (3.51) | 110 (7.12) |  |
| Never | 15,646 (4.22) | 15,543 (4.21) | 103 (6.67) |  |
| TDI,n(%) |  |  |  | <0.001 |
| Q1 | 93,125 (25.12) | 92,790 (25.13) | 335 (21.68) |  |
| Q2 | 92,549 (24.96) | 92,184 (24.97) | 365 (23.62) |  |
| Q3 | 92,530 (24.96) | 92,157 (24.96) | 373 (24.14) |  |
| Q4 | 92,540 (24.96) | 92,068 (24.94) | 472 (30.55) |  |
| History of anxiety, n(%) | | | | 0.070 |
| No | 162,265 (43.77) | 161,624 (43.78) | 641 (41.49) |  |
| Yes | 208,479 (56.23) | 207,575 (56.22) | 904 (58.51) |  |
| Family history of diabetes, n(%) | | | | 0.017 |
| No | 306,043 (82.55) | 304,732 (82.54) | 1,311 (84.85) |  |
| Yes | 64,701 (17.45) | 64,467 (17.46) | 234 (15.15) |  |
| History of stroke, n(%) | | | | <0.001 |
| No | 357,843 (96.52) | 356,752 (96.63) | 1,091 (70.61) |  |
| Yes | 12,901 (3.48) | 12,447 (3.37) | 454 (29.39) |  |
| SBP | 82 (75.00,89.00) | 82 (75.00,89.00) | 82 (75.00,89.00) | 0.900 |
| HDL-C | 1.40 (1.17,1.68) | 1.40 (1.17,1.68) | 1.31 (1.09,1.58) | <0.001 |
| CRP | 1.32 (0.66,2.75) | 1.32 (0.66,2.74) | 1.60 (0.77,3.36) | <0.001 |
| TyG | 8.68 (8.31,9.07) | 8.68 (8.31,9.07) | 8.84 (8.45,9.27) | <0.001 |
| ABSI | 0.08 (0.07,0.08) | 0.08 (0.07,0.08) | 0.08 (0.08,0.08) | <0.001 |
| TyG-WC | 781.72 (680.71,884.89) | 781.46 (680.52,884.60) | 837.37 (739.77,951.48) | <0.001 |
| TyG-BMI | 233.08 (204.50,267.04) | 233.02 (204.45,266.97) | 247.42 (216.46,283.93) | <0.001 |
| TyG-WHtR | 4.63 (4.08,5.20) | 4.62 (4.08,5.20) | 5.01 (4.45,5.64) | <0.001 |
| TyG-ABSI | 0.67 (0.62,0.72) | 0.67 (0.62,0.72) | 0.70 (0.65,0.75) | <0.001 |
| Abbreviations: TDI: Townsend Deprivation Index; SBP: Systolic Blood Pressure; HDL-C: High-Density Lipoprotein Cholesterol; CRP: C-Reactive Protein; TyG: Triglyceride glucose; ABSI: A body shape index; TyG-WC: Triglyceride-glucose index-Waist Circumference; TyG-BMI: Triglyceride-glucose index-Body Mass Index; TyG-WHtR: Triglyceride-glucose index-Waist-to-Height Ratio; TyG-ABSI: Triglyceride Glucose-A Body Shape Index.  **Table S18** For dementia, post-hoc tests were conducted to compare pairwise baseline characteristics that showed significant differences in global assessments.   \| variable/Group \| Q1 vs Q2 \| Q1 vs Q3 \| Q1 vs Q4 \| Q2 vs Q3 \| Q2 vs Q4 \| Q3 vsQ4 \| \| --- \| --- \| --- \| --- \| --- \| --- \| --- \| \| Age, n (%) \| ** \| ** \| ** \| ** \| ** \| ** \| \| Sex, n (%) \| ** \| ** \| ** \| ** \| ** \| ** \| \| Race, n (%) \| ** \| ** \| ** \| ns \| ** \| ** \| \| Residence, n (%) \| * \| ** \| ** \| ns \| ** \| ** \| \| Educational level, n (%) \| ** \| ** \| ** \| ** \| ** \| ** \| \| Smoking status, n (%) \| ** \| ** \| ** \| ** \| ** \| ** \| \| Drinking status, n (%) \| * \| ** \| ** \| * \| ** \| ** \| \| TDI, n (%) \| ** \| ** \| ** \| * \| ** \| ** \| \| History of anxiety, n (%) \| ** \| ** \| ** \| ** \| ** \| ** \| \| Family history of diabetes, n (%) \| ** \| ** \| ** \| ** \| ** \| ** \| \| History of stroke, n (%) \| ** \| ** \| ** \| ** \| ** \| ** \| \| SBP \| *** \| *** \| *** \| *** \| *** \| *** \| \| HDL-C \| *** \| *** \| *** \| *** \| *** \| *** \| \| CRP \| *** \| *** \| *** \| *** \| *** \| *** \| \| TyG \| *** \| *** \| *** \| *** \| *** \| *** \| \| ABSI \| *** \| *** \| *** \| *** \| *** \| *** \| \| TyG-WC \| *** \| *** \| *** \| *** \| *** \| *** \| \| TyG-BMI \| *** \| *** \| *** \| *** \| *** \| *** \| \| TyG-WHtR \| *** \| *** \| *** \| *** \| *** \| *** \| \| TyG-ABSI \| *** \| *** \| *** \| *** \| *** \| *** \| \| Note: ***p < 0.001, **p < 0.01, *p < 0.05; ns = not significant. All p-values are Holm-corrected. Q 1–4 correspond to tygabsi quartiles (lowest to highest). \| \| \| \| \| \| \| | | | | |

**Table S19** Cox results for standardized continuous TyG, ABSI, and TyG-ABSI associated with dementia and its subtypes.

| **Classify** | **Model1** | | **Model2** | | **Model3** | |
| --- | --- | --- | --- | --- | --- | --- |
|  | **HR (95%CI)** | ***P*** | **HR (95%CI)** | ***P*** | **HR (95%CI)** | ***P*** |
| **TyG** | | | | | | |
| ACD | 1.17 (1.15~1.20) | <0.001 | 1.09 (1.07~1.12) | <0.001 | 1.09 (1.06~1.12) | <0.001 |
| AD | 1.13 (1.09~1.17) | <0.001 | 1.07 (1.03~1.11) | <0.001 | 1.08 (1.04~1.13) | <0.001 |
| VD | 1.34 (1.28~1.41) | <0.001 | 1.24 (1.18~1.31) | <0.001 | 1.19 (1.12~1.26) | <0.001 |
| **ABSI** | | | | | | |
| ACD | 1.44 (1.41~1.48) | <0.001 | 1.31 (1.27~1.35) | <0.001 | 1.26 (1.22~1.30) | <0.001 |
| AD | 1.32 (1.27~1.36) | <0.001 | 1.22 (1.17~1.27) | <0.001 | 1.20 (1.15~1.25) | <0.001 |
| VD | 1.64 (1.57~1.72) | <0.001 | 1.41 (1.33~1.50) | <0.001 | 1.29 (1.21~1.37) | <0.001 |
| **TyG-ABSI** | | | | | | |
| ACD | 1.38 (1.35~1.42) | <0.001 | 1.25 (1.22~1.29) | <0.001 | 1.24 (1.21~1.28) | <0.001 |
| AD | 1.28 (1.23~1.32) | <0.001 | 1.18 (1.13~1.23) | <0.001 | 1.19 (1.14~1.25) | <0.001 |
| VD | 1.64 (1.56~1.72) | <0.001 | 1.44 (1.36~1.52) | <0.001 | 1.34 (1.26~1.43) | <0.001 |
| Abbreviations: TyG: Triglyceride glucose; ABSI: A body shape index; TyG-ABSI: Triglyceride Glucose-A Body Shape Index; HR (95% CI): Hazard Ratio (95% Confidence Interval); TDI: Townsend Deprivation Index; CRP: C-Reactive Protein; SBP: Systolic Blood Pressure; HDL-C: High-Density Lipoprotein Cholesterol.  Model1: Crude.  Model2: Adjusted for age, sex, race, and residence.  Model3: Age, sex, race, residence, educational level, smoking status and drinking status, TDI, history of anxiety, history of stroke, family history of diabetes, CRP, SBP, and HDL-C were adjusted for. | | | | | | |

**Table S20** Subgroup analysis for TyG of all-cause dementia.

| Vairable | Q1 | Q2 | Q3 | Q4 | *P* for interaction |
| --- | --- | --- | --- | --- | --- |
| Age |  |  |  |  | <0.001 |
| ≤65 | Ref. | 1.22(1.11~1.35) | 1.44(1.30~1.58) | 1.63(1.48~1.79) |  |
| ＞65 | Ref. | 0.89(0.81~0.99) | 0.81(0.73~0.89) | 0.90(0.81~0.99) |  |
| Sex |  |  |  |  | <0.001 |
| Male | Ref. | 1.00(0.90~1.11) | 0.95(0.86~1.05) | 0.98(0.89~1.08) |  |
| Female | Ref. | 1.38(1.26~1.53) | 1.63(1.48~1.80) | 1.96(1.78~2.17) |  |
| Race |  |  |  |  | 0.916 |
| White | Ref. | 1.23(1.14~1.32) | 1.32(1.23~1.42) | 1.48(1.38~1.58) |  |
| Others | Ref. | 1.27(0.90~1.79) | 1.31(0.92~1.86) | 1.35(0.96~1.90) |  |
| Residence |  |  |  |  | 0.691 |
| Urban | Ref. | 1.25(1.16~1.35) | 1.34(1.25~1.45) | 1.48(1.38~1.59) |  |
| Rural | Ref. | 1.14(0.94~1.39) | 1.24(1.02~1.51) | 1.48(1.23~1.79) |  |
| Educational level |  |  |  |  | 0.735 |
| College/Above | Ref. | 1.27(1.10~1.46) | 1.33(1.15~1.54) | 1.39(1.20~1.61) |  |
| Others | Ref. | 1.18(1.09~1.28) | 1.26(1.16~1.36) | 1.40(1.29~1.51) |  |
| Smoking status |  |  |  |  | 0.609 |
| Current | Ref. | 1.27(1.00~1.63) | 1.52(1.21~1.92) | 1.49(1.19~1.87) |  |
| Previous | Ref. | 1.26(1.13~1.41) | 1.26(1.13~1.41) | 1.46(1.31~1.62) |  |
| Never | Ref. | 1.19(1.08~1.31) | 1.30(1.18~1.44) | 1.41(1.27~1.55) |  |
| Drinking status |  |  |  |  | 0.047 |
| Current | Ref. | 1.27(1.18~1.37) | 1.37(1.27~1.47) | 1.47(1.37~1.58) |  |
| Previous | Ref. | 0.88(0.66~1.17) | 0.98(0.75~1.29) | 1.18(0.92~1.52) |  |
| Never | Ref. | 1.14(0.85~1.51) | 1.09(0.82~1.45) | 1.57(1.21~2.04) |  |
| TDI |  |  |  |  | 0.112 |
| Q1 | Ref. | 1.18(1.02~1.36) | 1.23(1.07~1.42) | 1.36(1.18~1.56) |  |
| Q2 | Ref. | 1.21(1.05~1.40) | 1.24(1.07~1.42) | 1.27(1.10~1.46) |  |
| Q3 | Ref. | 1.22(1.06~1.41) | 1.35(1.17~1.55) | 1.54(1.34~1.77) |  |
| Q4 | Ref. | 1.34(1.17~1.54) | 1.51(1.32~1.73) | 1.72(1.51~1.96) |  |
| History of anxiety |  |  |  |  | 0.657 |
| Yes | Ref. | 1.26(1.15~1.38) | 1.37(1.25~1.50) | 1.54(1.41~1.69) |  |
| No | Ref. | 1.21(1.08~1.35) | 1.28(1.15~1.43) | 1.42(1.27~1.57) |  |
| History of stroke |  |  |  |  | 0.036 |
| Yes | Ref. | 1.02(0.85~1.24) | 1.00(0.83~1.21) | 1.16(0.97~1.38) |  |
| No | Ref. | 1.24(1.15~1.34) | 1.34(1.25~1.45) | 1.44(1.34~1.56) |  |
| Family history of diabetes | | | | | 0.400 |
| Yes | Ref. | 1.25(1.02~1.53) | 1.48(1.22~1.80) | 1.67(1.39~2.01) |  |
| No | Ref. | 1.24(1.15~1.34) | 1.32(1.22~1.42) | 1.47(1.37~1.58) |  |
| Abbreviations: TDI: Townsend Deprivation index. | | | | | |

**Table S21** Subgroup analysis for ABSI of all-cause dementia.

| Variable | Q1 | Q2 | Q3 | Q4 | *P* for interaction |
| --- | --- | --- | --- | --- | --- |
| Age |  |  |  |  | <0.001 |
| ≤65 | Ref. | 1.27(1.14~1.41) | 1.59(1.44~1.76) | 2.41(2.19~2.65) |  |
| ＞65 | Ref. | 1.12(1.00~1.26) | 1.25(1.12~1.40) | 1.44(1.30~1.60) |  |
| Sex |  |  |  |  | 0.648 |
| Male | Ref. | 1.41(1.11~1.80) | 1.87(1.48~2.36) | 2.90(2.30~3.65) |  |
| Female | Ref. | 1.34(1.23~1.47) | 1.80(1.64~1.98) | 2.60(2.35~2.88) |  |
| Race |  |  |  |  | 0.071 |
| White | Ref. | 1.32(1.22~1.43) | 1.67(1.54~1.80) | 2.49(2.32~2.68) |  |
| Others | Ref. | 1.08(0.68~1.74) | 2.13(1.39~3.25) | 3.16(2.12~4.70) |  |
| Residence |  |  |  |  | 0.810 |
| Urban | Ref. | 1.30(1.19~1.41) | 1.69(1.56~1.83) | 2.51(2.33~2.70) |  |
| Rural | Ref. | 1.35(1.10~1.67) | 1.60(1.30~1.97) | 2.42(1.99~2.94) |  |
| Educational level |  |  |  |  | 0.560 |
| College/Above | Ref. | 1.38(1.17~1.64) | 1.84(1.57~2.16) | 2.63(2.25~3.06) |  |
| Others | Ref. | 1.28(1.17~1.40) | 1.61(1.48~1.76) | 2.41(2.23~2.61) |  |
| Smoking status |  |  |  |  | 0.070 |
| Current | Ref. | 1.12(0.84~1.49) | 1.66(1.28~2.15) | 2.53(1.98~3.22) |  |
| Previous | Ref. | 1.44(1.26~1.64) | 1.85(1.63~2.09) | 2.69(2.39~3.02) |  |
| Never | Ref. | 1.24(1.12~1.38) | 1.50(1.36~1.67) | 2.19(1.99~2.42) |  |
| Drinking status |  |  |  |  | 0.029 |
| Current | Ref. | 1.35(1.24~1.47) | 1.74(1.61~1.89) | 2.60(2.41~2.81) |  |
| Previous | Ref. | 1.16(0.85~1.58) | 1.27(0.94~1.72) | 2.20(1.69~2.88) |  |
| Never | Ref. | 1.06(0.80~1.40) | 1.51(1.16~1.96) | 1.71(1.32~2.20) |  |
| TDI |  |  |  |  | 0.408 |
| Q1 | Ref. | 1.22(1.05~1.43) | 1.55(1.34~1.80) | 2.18(1.90~2.51) |  |
| Q2 | Ref. | 1.21(1.03~1.41) | 1.66(1.43~1.92) | 2.35(2.04~2.70) |  |
| Q3 | Ref. | 1.47(1.25~1.72) | 1.82(1.56~2.12) | 2.75(2.38~3.18) |  |
| Q4 | Ref. | 1.34(1.15~1.57) | 1.69(1.45~1.96) | 2.68(2.33~3.07) |  |
| History of anxiety |  |  |  |  | 0.046 |
| Yes | Ref. | 1.31(1.19~1.44) | 1.59(1.45~1.75) | 2.40(2.20~2.63) |  |
| No | Ref. | 1.34(1.17~1.54) | 1.89(1.67~2.14) | 2.79(2.47~3.13) |  |
| History of stroke |  |  |  |  | 0.003 |
| Yes | Ref. | 1.26(1.00~1.58) | 1.43(1.15~1.78) | 1.75(1.43~2.15) |  |
| No | Ref. | 1.27(1.16~1.38) | 1.60(1.48~1.74) | 2.36(2.19~2.54) |  |
| Family history of diabetes | | | | | 0.133 |
| Yes | Ref. | 1.36(1.09~1.69) | 2.01(1.64~2.46) | 2.92(2.41~3.55) |  |
| No | Ref. | 1.30(1.20~1.42) | 1.63(1.51~1.77) | 2.44(2.27~2.64) |  |
| Abbreviations: TDI: Townsend Deprivation index. | | | | | |

**Table S22** Subgroup analysis for TyG-ABSI of all-cause dementia.

| Variable | Q1 | Q2 | Q3 | Q4 | *P* for interaction |
| --- | --- | --- | --- | --- | --- |
| Age | | | | | <0.001 |
| ≤65 | Ref. | 1.33(1.20~1.48) | 1.73(1.57~1.92) | 2.34(2.12~2.58) |  |
| ＞65 | Ref. | 1.07(0.96~1.20) | 1.09(0.98~1.22) | 1.24(1.11~1.38) |  |
| Sex |  |  |  |  | <0.001 |
| Male | Ref. | 1.19(1.00~1.40) | 1.42(1.21~1.67) | 1.78(1.52~2.08) |  |
| Female | Ref. | 1.47(1.34~1.60) | 1.87(1.70~2.06) | 2.70(2.45~2.99) |  |
| Race |  |  |  |  | 0.235 |
| White | Ref. | 1.45(1.34~1.57) | 1.79(1.65~1.93) | 2.32(2.16~2.50) |  |
| Others | Ref. | 1.20(0.78~1.84) | 2.05(1.39~3.02) | 2.71(1.88~3.90) |  |
| Residence |  |  |  |  | 0.411 |
| Urban | Ref. | 1.42(1.30~1.54) | 1.80(1.66~1.95) | 2.33(2.16~2.52) |  |
| Rural | Ref. | 1.63(1.31~2.01) | 1.79(1.45~2.20) | 2.32(1.89~2.84) |  |
| Educational level |  |  |  |  | 0.601 |
| College/Above | Ref. | 1.42(1.21~1.66) | 1.87(1.61~2.18) | 2.23(1.92~2.60) |  |
| Others | Ref. | 1.41(1.29~1.54) | 1.71(1.57~1.86) | 2.23(2.06~2.42) |  |
| Smoking status |  |  |  |  | 0.053 |
| Current | Ref. | 1.14(0.86~1.50) | 1.76(1.36~2.27) | 2.18(1.71~2.78) |  |
| Previous | Ref. | 1.43(1.26~1.62) | 1.78(1.58~2.01) | 2.40(2.14~2.69) |  |
| Never | Ref. | 1.48(1.33~1.64) | 1.73(1.56~1.92) | 2.09(1.89~2.31) |  |
| Drinking status |  |  |  |  | 0.777 |
| Current | Ref. | 1.45(1.33~1.57) | 1.82(1.68~1.97) | 2.36(2.18~2.54) |  |
| Previous | Ref. | 1.22(0.89~1.67) | 1.53(1.14~2.06) | 1.93(1.46~2.55) |  |
| Never | Ref. | 1.55(1.16~2.08) | 1.71(1.28~2.28) | 2.18(1.65~2.87) |  |
| TDI |  |  |  |  | 0.002 |
| Q1 | Ref. | 1.34(1.15~1.56) | 1.65(1.43~1.92) | 2.02(1.75~2.33) |  |
| Q2 | Ref. | 1.42(1.22~1.65) | 1.61(1.39~1.87) | 2.07(1.79~2.39) |  |
| Q3 | Ref. | 1.74(1.48~2.04) | 1.95(1.67~2.28) | 2.68(2.31~3.11) |  |
| Q4 | Ref. | 1.31(1.12~1.53) | 1.99(1.72~2.30) | 2.55(2.22~2.93) |  |
| History of anxiety | | | | | 0.282 |
| Yes | Ref. | 1.45(1.32~1.60) | 1.73(1.58~1.90) | 2.31(2.11~2.53) |  |
| No | Ref. | 1.46(1.28~1.66) | 1.96(1.73~2.21) | 2.47(2.19~2.77) |  |
| History of stroke |  |  |  |  | 0.016 |
| Yes | Ref. | 1.01(0.80~1.26) | 1.37(1.11~1.69) | 1.59(1.30~1.94) |  |
| No | Ref. | 1.44(1.33~1.57) | 1.73(1.60~1.87) | 2.21(2.05~2.39) |  |
| Family history of diabetes | | | | | 0.078 |
| Yes | Ref. | 1.54(1.23~1.92) | 2.00(1.62~2.47) | 2.90(2.38~3.55) |  |
| No | Ref. | 1.44(1.32~1.56) | 1.78(1.65~1.93) | 2.28(2.11~2.46) |  |
| Abbreviations: TDI: Townsend Deprivation Index. | | | | | |

**Table S23** Subgroup analysis for TyG of Alzheimer's dementia.

| Variable | Q1 | Q2 | Q3 | Q4 | *P* for interaction |
| --- | --- | --- | --- | --- | --- |
| Age |  |  |  |  | <0.001 |
| ≤65 | Ref. | 1.26(1.08~1.47) | 1.51(1.30~1.75) | 1.53(1.32~1.77) |  |
| ＞65 | Ref. | 0.87(0.75~1.00) | 0.80(0.69~0.92) | 0.81(0.70~0.93) |  |
| Sex |  |  |  |  | <0.001 |
| Male | Ref. | 1.02(0.86~1.20) | 1.03(0.88~1.20) | 0.94(0.81~1.10) |  |
| Female | Ref. | 1.38(1.20~1.58) | 1.59(1.38~1.83) | 1.80(1.56~2.08) |  |
| Race |  |  |  |  | 0.544 |
| White | Ref. | 1.25(1.12~1.39) | 1.36(1.22~1.51) | 1.36(1.23~1.52) |  |
| Others | Ref. | 0.89(0.52~1.52) | 1.00(0.59~1.70) | 1.00(0.59~1.68) |  |
| Residence |  |  |  |  | 0.889 |
| Urban | Ref. | 1.25(1.12~1.40) | 1.36(1.22~1.52) | 1.35(1.21~1.51) |  |
| Rural | Ref. | 1.14(0.85~1.53) | 1.32(0.99~1.75) | 1.37(1.03~1.82) |  |
| Educational level |  |  |  |  | 0.461 |
| College/Above | Ref. | 1.06(0.85~1.31) | 1.22(0.98~1.51) | 1.11(0.88~1.39) |  |
| Others | Ref. | 1.25(1.11~1.41) | 1.32(1.17~1.49) | 1.32(1.18~1.49) |  |
| Smoking status |  |  |  |  | 0.722 |
| Current | Ref. | 1.11(0.75~1.65) | 1.60(1.11~2.30) | 1.38(0.96~1.99) |  |
| Previous | Ref. | 1.30(1.10~1.53) | 1.34(1.14~1.58) | 1.35(1.15~1.59) |  |
| Never | Ref. | 1.19(1.03~1.38) | 1.29(1.12~1.49) | 1.30(1.12~1.50) |  |
| Drinking status |  |  |  |  | 0.709 |
| Current | Ref. | 1.25(1.12~1.40) | 1.37(1.22~1.52) | 1.33(1.19~1.49) |  |
| Previous | Ref. | 1.12(0.71~1.76) | 1.31(0.85~2.01) | 1.18(0.77~1.81) |  |
| Never | Ref. | 1.15(0.76~1.75) | 1.11(0.73~1.69) | 1.51(1.03~2.23) |  |
| TDI |  |  |  |  | 0.281 |
| Q1 | Ref. | 1.09(0.89~1.34) | 1.17(0.95~1.43) | 1.22(0.99~1.49) |  |
| Q2 | Ref. | 1.20(0.98~1.48) | 1.22(0.99~1.51) | 1.15(0.93~1.42) |  |
| Q3 | Ref. | 1.44(1.16~1.79) | 1.51(1.22~1.86) | 1.50(1.22~1.86) |  |
| Q4 | Ref. | 1.26(1.01~1.56) | 1.56(1.28~1.92) | 1.56(1.28~1.91) |  |
| History of anxiety |  |  |  |  | 0.966 |
| Yes | Ref. | 1.26(1.10~1.44) | 1.39(1.21~1.59) | 1.37(1.20~1.57) |  |
| No | Ref. | 1.22(1.03~1.44) | 1.31(1.12~1.55) | 1.34(1.14~1.58) |  |
| History of stroke |  |  |  |  | 0.182 |
| Yes | Ref. | 1.46(1.02~2.08) | 1.28(0.90~1.84) | 1.13(0.79~1.61) |  |
| No | Ref. | 1.20(1.08~1.34) | 1.34(1.20~1.49) | 1.34(1.20~1.49) |  |
| Family history of diabetes | | | | | 0.374 |
| Yes | Ref. | 1.28(0.94~1.76) | 1.69(1.25~2.27) | 1.54(1.15~2.06) |  |
| No | Ref. | 1.24(1.11~1.38) | 1.32(1.18~1.47) | 1.35(1.21~1.51) |  |
| Abbreviations: TDI: Townsend Deprivation Index. | | | | | |

**Table S24** Subgroup analysis for ABSI of Alzheimer's dementia.

| Variable | Q1 | Q2 | Q3 | Q4 | *P* for interaction |
| --- | --- | --- | --- | --- | --- |
| Age |  |  |  |  | <0.001 |
| ≤65 | Ref. | 1.17(1.00~1.36) | 1.37(1.18~1.59) | 1.84(1.59~2.13) |  |
| ＞65 | Ref. | 1.08(0.92~1.27) | 1.05(0.90~1.22) | 1.15(0.99~1.33) |  |
| Sex |  |  |  |  | 0.772 |
| Male | Ref. | 1.47(1.00~2.14) | 1.87(1.30~2.70) | 2.70(1.89~3.87) |  |
| Female | Ref. | 1.32(1.16~1.49) | 1.61(1.40~1.84) | 2.26(1.94~2.62) |  |
| Race |  |  |  |  | 0.006 |
| White | Ref. | 1.26(1.12~1.41) | 1.41(1.27~1.58) | 1.95(1.76~2.17) |  |
| Others | Ref. | 0.70(0.32~1.53) | 2.22(1.19~4.15) | 2.79(1.53~5.07) |  |
| Residence |  |  |  |  | 0.937 |
| Urban | Ref. | 1.24(1.10~1.40) | 1.42(1.27~1.60) | 1.98(1.77~2.21) |  |
| Rural | Ref. | 1.21(0.89~1.63) | 1.47(1.10~1.97) | 1.87(1.41~2.48) |  |
| Educational level |  |  |  |  | 0.467 |
| College/Above | Ref. | 1.13(0.88~1.44) | 1.52(1.21~1.92) | 1.97(1.57~2.46) |  |
| Others | Ref. | 1.26(1.11~1.43) | 1.39(1.23~1.57) | 1.92(1.71~2.15) |  |
| Smoking status |  |  |  |  | 0.706 |
| Current | Ref. | 1.04(0.68~1.59) | 1.35(0.91~2.00) | 1.91(1.32~2.74) |  |
| Previous | Ref. | 1.39(1.16~1.67) | 1.51(1.26~1.80) | 1.99(1.68~2.35) |  |
| Never | Ref. | 1.15(0.99~1.34) | 1.34(1.16~1.56) | 1.86(1.62~2.15) |  |
| Drinking status |  |  |  |  | 0.200 |
| Current | Ref. | 1.29(1.14~1.45) | 1.48(1.31~1.66) | 2.03(1.82~2.27) |  |
| Previous | Ref. | 0.89(0.57~1.39) | 0.84(0.53~1.33) | 1.43(0.97~2.12) |  |
| Never | Ref. | 1.04(0.69~1.58) | 1.62(1.10~2.39) | 1.75(1.20~2.55) |  |
| TDI |  |  |  |  | 0.998 |
| Q1 | Ref. | 1.28(1.03~1.59) | 1.44(1.16~1.78) | 1.92(1.56~2.35) |  |
| Q2 | Ref. | 1.17(0.93~1.47) | 1.43(1.15~1.78) | 1.92(1.56~2.36) |  |
| Q3 | Ref. | 1.29(1.03~1.61) | 1.41(1.13~1.75) | 2.06(1.68~2.54) |  |
| Q4 | Ref. | 1.21(0.96~1.51) | 1.43(1.15~1.78) | 1.94(1.58~2.38) |  |
| History of anxiety |  |  |  |  | 0.051 |
| Yes | Ref. | 1.29(1.12~1.48) | 1.33(1.15~1.53) | 1.95(1.71~2.22) |  |
| No | Ref. | 1.18(0.98~1.42) | 1.62(1.36~1.93) | 2.07(1.75~2.44) |  |
| History of stroke |  |  |  |  | 0.154 |
| Yes | Ref. | 1.11(0.73~1.68) | 1.36(0.92~2.01) | 1.39(0.95~2.01) |  |
| No | Ref. | 1.23(1.09~1.38) | 1.38(1.24~1.55) | 1.92(1.72~2.14) |  |
| Family history of diabetes | | | | | 0.220 |
| Yes | Ref. | 1.19(0.86~1.64) | 1.59(1.17~2.15) | 2.40(1.80~3.18) |  |
| No | Ref. | 1.25(1.11~1.41) | 1.41(1.26~1.59) | 1.91(1.71~2.13) |  |
| Abbreviations: TDI: Townsend Deprivation Index. | | | | | |

**Table S25** Subgroup analysis for TyG-ABSI of Alzheimer's dementia.

| Variable | Q1 | Q2 | Q3 | Q4 | *P* for interaction |
| --- | --- | --- | --- | --- | --- |
| Age |  |  |  |  | <0.001 |
| ≤65 | Ref. | 1.34(1.15~1.57) | 1.65(1.41~1.92) | 1.89(1.63~2.20) |  |
| ＞65 | Ref. | 1.02(0.87~1.19) | 0.98(0.84~1.15) | 1.01(0.87~1.17) |  |
| Sex |  |  |  |  | 0.097 |
| Male | Ref. | 1.17(0.90~1.52) | 1.47(1.15~1.89) | 1.64(1.29~2.10) |  |
| Female | Ref. | 1.54(1.35~1.75) | 1.82(1.59~2.08) | 2.35(2.03~2.73) |  |
| Race |  |  |  |  | 0.392 |
| White | Ref. | 1.43(1.27~1.60) | 1.65(1.48~1.85) | 1.90(1.70~2.11) |  |
| Others | Ref. | 1.25(0.65~2.41) | 2.29(1.27~4.12) | 2.33(1.31~4.16) |  |
| Residence |  |  |  |  | 0.924 |
| Urban | Ref. | 1.40(1.24~1.58) | 1.66(1.48~1.87) | 1.90(1.69~2.13) |  |
| Rural | Ref. | 1.57(1.16~2.13) | 1.77(1.31~2.39) | 1.99(1.48~2.67) |  |
| Educational level |  |  |  |  | 0.953 |
| College/Above | Ref. | 1.31(1.04~1.66) | 1.55(1.24~1.95) | 1.77(1.41~2.22) |  |
| Others | Ref. | 1.42(1.25~1.61) | 1.64(1.45~1.86) | 1.84(1.63~2.08) |  |
| Smoking status |  |  |  |  | 0.738 |
| Current | Ref. | 1.14(0.74~1.76) | 1.59(1.07~2.37) | 1.92(1.31~2.80) |  |
| Previous | Ref. | 1.42(1.18~1.71) | 1.70(1.42~2.02) | 1.92(1.62~2.28) |  |
| Never | Ref. | 1.44(1.24~1.67) | 1.61(1.39~1.87) | 1.76(1.52~2.05) |  |
| Drinking status |  |  |  |  | 0.577 |
| Current | Ref. | 1.40(1.24~1.57) | 1.66(1.48~1.86) | 1.90(1.69~2.12) |  |
| Previous | Ref. | 1.22(0.77~1.96) | 1.52(0.97~2.37) | 1.42(0.91~2.20) |  |
| Never | Ref. | 2.01(1.29~3.14) | 2.06(1.32~3.23) | 2.51(1.63~3.88) |  |
| TDI |  |  |  |  | 0.284 |
| Q1 | Ref. | 1.35(1.09~1.68) | 1.57(1.27~1.94) | 1.75(1.42~2.16) |  |
| Q2 | Ref. | 1.35(1.08~1.68) | 1.41(1.13~1.75) | 1.67(1.35~2.06) |  |
| Q3 | Ref. | 1.73(1.37~2.18) | 1.89(1.50~2.37) | 2.24(1.79~2.79) |  |
| Q4 | Ref. | 1.32(1.04~1.66) | 1.88(1.52~2.33) | 2.03(1.64~2.50) |  |
| History of anxiety |  |  |  |  | 0.658 |
| Yes | Ref. | 1.44(1.25~1.65) | 1.62(1.41~1.86) | 1.88(1.64~2.15) |  |
| No | Ref. | 1.43(1.18~1.72) | 1.80(1.51~2.14) | 2.02(1.70~2.40) |  |
| History of stroke |  |  |  |  | 0.547 |
| Yes | Ref. | 1.39(0.89~2.16) | 1.82(1.20~2.75) | 1.67(1.11~2.51) |  |
| No | Ref. | 1.40(1.24~1.57) | 1.60(1.43~1.79) | 1.84(1.64~2.05) |  |
| Family history of diabetes | | | | | 0.106 |
| Yes | Ref. | 1.26(0.91~1.76) | 1.77(1.30~2.41) | 2.31(1.72~3.10) |  |
| No | Ref. | 1.45(1.29~1.64) | 1.67(1.49~1.88) | 1.87(1.67~2.10) |  |
| Abbreviations: TDI: Townsend Deprivation Index. | | | | | |

**Table S26** Subgroup analysis for TyG of vascular dementia.

| Variable | Q1 | Q2 | Q3 | Q4 | *P* for interaction |
| --- | --- | --- | --- | --- | --- |
| Age |  |  |  |  | <0.001 |
| ≤65 | Ref. | 1.27(0.99~1.61) | 1.72(1.37~2.16) | 2.50(2.02~3.10) |  |
| ＞65 | Ref. | 0.79(0.64~0.98) | 0.80(0.65~0.98) | 1.09(0.90~1.33) |  |
| Sex |  |  |  |  | <0.001 |
| Male | Ref. | 0.94(0.75~1.17) | 1.05(0.86~1.30) | 1.32(1.09~1.59) |  |
| Female | Ref. | 1.35(1.07~1.70) | 1.63(1.30~2.06) | 2.50(2.00~3.12) |  |
| Race |  |  |  |  | 0.471 |
| White | Ref. | 1.16(0.98~1.36) | 1.42(1.21~1.66) | 2.00(1.72~2.31) |  |
| Others | Ref. | 1.69(0.81~3.51) | 1.48(0.69~3.20) | 1.63(0.77~3.42) |  |
| Residence |  |  |  |  | 0.912 |
| Urban | Ref. | 1.17(0.99~1.39) | 1.44(1.22~1.69) | 1.97(1.69~2.30) |  |
| Rural | Ref. | 1.28(0.78~2.10) | 1.39(0.85~2.25) | 2.19(1.40~3.43) |  |
| Educational level |  |  |  |  | 0.982 |
| College/Above | Ref. | 1.20(0.84~1.72) | 1.41(0.99~2.01) | 1.97(1.41~2.75) |  |
| Others | Ref. | 1.12(0.94~1.34) | 1.34(1.13~1.58) | 1.83(1.56~2.14) |  |
| Smoking status |  |  |  |  | 0.078 |
| Current | Ref. | 1.06(0.63~1.78) | 1.95(1.23~3.08) | 1.59(1.01~2.52) |  |
| Previous | Ref. | 1.27(1.00~1.63) | 1.34(1.05~1.70) | 2.10(1.69~2.62) |  |
| Never | Ref. | 1.10(0.87~1.38) | 1.32(1.05~1.65) | 1.83(1.48~2.26) |  |
| Drinking status |  |  |  |  | 0.122 |
| Current | Ref. | 1.25(1.05~1.48) | 1.52(1.29~1.80) | 2.05(1.75~2.40) |  |
| Previous | Ref. | 0.87(0.47~1.61) | 1.26(0.72~2.20) | 1.54(0.91~2.61) |  |
| Never | Ref. | 0.78(0.43~1.42) | 0.59(0.31~1.12) | 1.48(0.90~2.44) |  |
| TDI |  |  |  |  | 0.732 |
| Q1 | Ref. | 1.18(0.84~1.65) | 1.40(1.01~1.93) | 1.94(1.43~2.64) |  |
| Q2 | Ref. | 1.36(0.99~1.87) | 1.41(1.03~1.93) | 1.80(1.33~2.43) |  |
| Q3 | Ref. | 1.02(0.74~1.41) | 1.29(0.95~1.75) | 1.81(1.36~2.41) |  |
| Q4 | Ref. | 1.19(0.88~1.62) | 1.63(1.22~2.16) | 2.33(1.79~3.04) |  |
| History of anxiety |  |  |  |  | 0.753 |
| Yes | Ref. | 1.23(1.00~1.52) | 1.49(1.22~1.82) | 2.14(1.77~2.59) |  |
| No | Ref. | 1.12(0.87~1.43) | 1.36(1.07~1.72) | 1.82(1.46~2.28) |  |
| History of stroke |  |  |  |  | 0.219 |
| Yes | Ref. | 0.90(0.67~1.22) | 1.07(0.80~1.42) | 1.41(1.08~1.84) |  |
| No | Ref. | 1.23(1.02~1.48) | 1.46(1.22~1.75) | 1.93(1.62~2.29) |  |
| Family history of diabetes | | | | | 0.659 |
| Yes | Ref. | 1.33(0.85~2.08) | 1.42(0.92~2.19) | 2.36(1.59~3.49) |  |
| No | Ref. | 1.17(0.98~1.38) | 1.44(1.23~1.70) | 1.96(1.68~2.29) |  |
| Abbreviations: TDI: Townsend Deprivation Index. | | | | | |

**Table S27** Subgroup analysis for ABSI of vascular dementia.

| Variable | Q1 | Q2 | Q3 | Q4 | *P* for interaction |
| --- | --- | --- | --- | --- | --- |
| Age |  |  |  |  | <0.001 |
| ≤65 | Ref. | 2.02(1.54~2.66) | 2.76(2.12~3.59) | 4.78(3.72~6.13) |  |
| ＞65 | Ref. | 1.07(0.83~1.37) | 1.18(0.94~1.49) | 1.68(1.36~2.08) |  |
| Sex |  |  |  |  | 0.552 |
| Male | Ref. | 1.75(1.00~3.06) | 2.30(1.34~3.93) | 4.19(2.47~7.12) |  |
| Female | Ref. | 1.57(1.28~1.93) | 2.10(1.69~2.62) | 3.22(2.55~4.06) |  |
| Race |  |  |  |  | 0.572 |
| White | Ref. | 1.63(1.35~1.97) | 2.19(1.83~2.61) | 3.85(3.26~4.54) |  |
| Others | Ref. | 0.98(0.40~2.42) | 1.15(0.48~2.77) | 2.59(1.22~5.51) |  |
| Residence |  |  |  |  | 0.417 |
| Urban | Ref. | 1.63(1.35~1.98) | 2.09(1.73~2.51) | 3.73(3.14~4.43) |  |
| Rural | Ref. | 1.26(0.71~2.25) | 2.46(1.47~4.12) | 3.97(2.43~6.46) |  |
| Educational level |  |  |  |  | 0.033 |
| College/Above | Ref. | 2.11(1.36~3.27) | 3.00(1.97~4.55) | 3.68(2.44~5.55) |  |
| Others | Ref. | 1.49(1.22~1.82) | 1.94(1.60~2.35) | 3.66(3.07~4.36) |  |
| Smoking status |  |  |  |  | 0.191 |
| Current | Ref. | 1.78(0.94~3.35) | 1.85(0.99~3.44) | 4.62(2.64~8.09) |  |
| Previous | Ref. | 1.76(1.29~2.39) | 2.42(1.81~3.23) | 4.18(3.19~5.49) |  |
| Never | Ref. | 1.45(1.13~1.85) | 1.90(1.50~2.41) | 2.95(2.36~3.70) |  |
| Drinking status |  |  |  |  | 0.116 |
| Current | Ref. | 1.64(1.34~2.00) | 2.24(1.86~2.71) | 3.94(3.30~4.70) |  |
| Previous | Ref. | 1.77(0.82~3.83) | 2.16(1.02~4.57) | 5.00(2.56~9.77) |  |
| Never | Ref. | 1.26(0.71~2.24) | 1.43(0.81~2.54) | 1.76(1.02~3.05) |  |
| TDI |  |  |  |  | 0.883 |
| Q1 | Ref. | 1.83(1.24~2.68) | 2.37(1.64~3.43) | 3.81(2.69~5.41) |  |
| Q2 | Ref. | 1.31(0.90~1.90) | 2.13(1.52~3.00) | 3.47(2.52~4.77) |  |
| Q3 | Ref. | 1.73(1.20~2.49) | 2.09(1.46~2.99) | 3.73(2.68~5.20) |  |
| Q4 | Ref. | 1.55(1.10~2.18) | 1.95(1.40~2.71) | 3.84(2.84~5.20) |  |
| History of anxiety |  |  |  |  | 0.193 |
| Yes | Ref. | 1.58(1.26~1.97) | 1.98(1.59~2.46) | 3.42(2.80~4.17) |  |
| No | Ref. | 1.70(1.24~2.35) | 2.55(1.89~3.44) | 4.66(3.51~6.18) |  |
| History of stroke |  |  |  |  | 0.030 |
| Yes | Ref. | 1.57(1.08~2.29) | 1.53(1.06~2.20) | 2.36(1.69~3.31) |  |
| No | Ref. | 1.45(1.17~1.78) | 2.00(1.64~2.44) | 3.30(2.74~3.98) |  |
| Family history of diabetes | | | | | 0.711 |
| Yes | Ref. | 1.48(0.90~2.42) | 2.39(1.52~3.77) | 4.14(2.69~6.36) |  |
| No | Ref. | 1.62(1.33~1.97) | 2.09(1.73~2.53) | 3.72(3.12~4.43) |  |
| Abbreviations: TDI: Townsend Deprivation Index. | | | | | |

**Table S28** Subgroup analysis for TyG-ABSI of vascular dementia.

| Variable | Q1 | Q2 | Q3 | Q4 | *P* for interaction |
| --- | --- | --- | --- | --- | --- |
| Age |  |  |  |  | <0.001 |
| ≤65 | Ref. | 1.71(1.30~2.24) | 2.74(2.12~3.53) | 4.34(3.41~5.53) |  |
| ＞65 | Ref. | 0.99(0.78~1.27) | 1.09(0.87~1.38) | 1.52(1.22~1.89) |  |
| Sex |  |  |  |  | 0.352 |
| Male | Ref. | 1.34(0.92~1.95) | 1.69(1.18~2.42) | 2.58(1.82~3.65) |  |
| Female | Ref. | 1.49(1.20~1.86) | 2.26(1.81~2.81) | 3.47(2.77~4.34) |  |
| Race |  |  |  |  | 0.247 |
| White | Ref. | 1.64(1.36~1.97) | 2.31(1.94~2.75) | 3.63(3.07~4.28) |  |
| Others | Ref. | 0.65(0.26~1.66) | 1.62(0.77~3.43) | 2.07(1.03~4.19) |  |
| Residence |  |  |  |  | 0.881 |
| Urban | Ref. | 1.54(1.27~1.87) | 2.22(1.85~2.65) | 3.44(2.90~4.08) |  |
| Rural | Ref. | 1.92(1.08~3.42) | 2.72(1.57~4.70) | 4.25(2.53~7.16) |  |
| Educational level |  |  |  |  | 0.329 |
| College/Above | Ref. | 2.00(1.34~3.00) | 2.44(1.64~3.62) | 3.37(2.30~4.94) |  |
| Others | Ref. | 1.44(1.17~1.76) | 2.12(1.75~2.56) | 3.30(2.76~3.95) |  |
| Smoking status |  |  |  |  | 0.093 |
| Current | Ref. | 0.73(0.40~1.33) | 1.70(1.03~2.80) | 2.32(1.45~3.71) |  |
| Previous | Ref. | 1.76(1.29~2.38) | 2.46(1.85~3.27) | 4.01(3.06~5.25) |  |
| Never | Ref. | 1.63(1.28~2.09) | 2.12(1.67~2.69) | 3.03(2.41~3.82) |  |
| Drinking status |  |  |  |  | 0.098 |
| Current | Ref. | 1.69(1.39~2.06) | 2.30(1.91~2.77) | 3.64(3.05~4.34) |  |
| Previous | Ref. | 1.37(0.58~3.20) | 3.67(1.77~7.62) | 4.47(2.21~9.08) |  |
| Never | Ref. | 0.81(0.43~1.54) | 1.32(0.74~2.34) | 1.95(1.15~3.32) |  |
| TDI |  |  |  |  | 0.056 |
| Q1 | Ref. | 1.78(1.21~2.61) | 2.52(1.75~3.63) | 3.67(2.58~5.22) |  |
| Q2 | Ref. | 2.07(1.43~3.00) | 2.50(1.74~3.59) | 3.67(2.60~5.19) |  |
| Q3 | Ref. | 1.45(1.03~2.05) | 1.61(1.15~2.26) | 2.98(2.19~4.05) |  |
| Q4 | Ref. | 1.18(0.82~1.70) | 2.52(1.84~3.46) | 3.69(2.73~4.99) |  |
| History of anxiety |  |  |  |  | 0.765 |
| Yes | Ref. | 1.58(1.26~1.99) | 2.16(1.74~2.68) | 3.44(2.81~4.21) |  |
| No | Ref. | 1.61(1.18~2.19) | 2.52(1.89~3.35) | 3.83(2.92~5.03) |  |
| History of stroke |  |  |  |  | 0.048 |
| Yes | Ref. | 0.98(0.68~1.41) | 1.37(0.99~1.91) | 1.91(1.40~2.61) |  |
| No | Ref. | 1.63(1.32~2.01) | 2.20(1.80~2.69) | 3.26(2.70~3.94) |  |
| Family history of diabetes | | | | | 0.744 |
| Yes | Ref. | 1.69(1.00~2.84) | 2.66(1.64~4.32) | 4.34(2.74~6.85) |  |
| No | Ref. | 1.57(1.30~1.91) | 2.23(1.85~2.67) | 3.45(2.90~4.10) |  |
| Abbreviations: TDI: Townsend Deprivation Index. | | | | | |

**Table S29** Sensitivity analysis for Cox regression analyses of TyG, ABSI and TyG-ABSI indices with dementia and subtypes excluding participants who developed dementia within 5 years after the follow-up period.

| Classify | Model1 | | Model2 | | Model3 | |
| --- | --- | --- | --- | --- | --- | --- |
|  | HR (95%CI) | *P* | HR (95%CI) | *P* | HR (95%CI) | *P* |
| **TyG** | | | | | | |
| **ACD** |  |  |  |  |  |  |
| Q1 | Ref. |  | Ref. |  | Ref. |  |
| Q2 | 1.26 (1.17~1.35) | <0.001 | 1.07 (1.00~1.16) | 0.061 | 1.08 (1.00~1.16) | 0.052 |
| Q3 | 1.33 (1.24~1.43) | <0.001 | 1.08 (1.00~1.16) | 0.046 | 1.08 (1.00~1.17) | 0.044 |
| Q4 | 1.52 (1.41~1.63) | <0.001 | 1.22 (1.14~1.31) | <0.001 | 1.20 (1.11~1.30) | <0.001 |
| **AD** |  |  |  |  |  |  |
| Q1 | Ref. |  | Ref. |  | Ref. |  |
| Q2 | 1.24 (1.11~1.38) | <0.001 | 1.06 (0.95~1.18) | 0.334 | 1.07 (0.96~1.20) | 0.213 |
| Q3 | 1.35 (1.21~1.50) | <0.001 | 1.10 (0.99~1.22) | 0.092 | 1.13 (1.01~1.26) | 0.036 |
| Q4 | 1.40(1.26~1.56) | <0.001 | 1.16 (1.04~1.29) | 0.008 | 1.19 (1.05~1.34) | 0.005 |
| **VD** |  |  |  |  |  |  |
| Q1 | Ref. |  | Ref. |  | Ref. |  |
| Q2 | 1.18 (1.00~1.40) | 0.051 | 0.98 (0.83~1.16) | 0.837 | 0.94 (0.80~1.12) | 0.506 |
| Q3 | 1.45 (1.24~1.70) | <0.001 | 1.13 (0.96~1.32) | 0.150 | 1.05 (0.88~1.24) | 0.598 |
| Q4 | 2.00 (1.72~2.33) | <0.001 | 1.52 (1.30~1.77) | <0.001 | 1.31 (1.11~1.55) | 0.002 |
| **ABSI** | | | | | | |
| **ACD** |  |  |  |  |  |  |
| Q1 | Ref. |  | Ref. |  | Ref. |  |
| Q2 | 1.28 (1.18~1.38) | <0.001 | 1.20 (1.10~1.30) | <0.001 | 1.18 (1.09~1.29) | <0.001 |
| Q3 | 1.63 (1.51~1.76) | <0.001 | 1.46 (1.33~1.57) | <0.001 | 1.39 (1.28~1.51) | <0.001 |
| Q4 | 2.44 (2.27~2.63) | <0.001 | 1.92 (1.77~2.09) | <0.001 | 1.76 (1.61~1.92) | <0.001 |
| **AD** |  |  |  |  |  |  |
| Q1 | Ref. |  | Ref. |  | Ref. |  |
| Q2 | 1.21 (1.08~1.36) | 0.001 | 1.16 (1.03~1.30) | 0.013 | 1.16 (1.03~1.31) | 0.012 |
| Q3 | 1.41 (1.26~1.57) | <0.001 | 1.31 (1.16~1.47) | <0.001 | 1.29 (1.14~1.46) | <0.001 |
| Q4 | 1.95 (1.75~2.16) | <0.001 | 1.63 (1.44~1.84) | <0.001 | 1.57 (1.38~1.78) | <0.001 |
| **VD** |  |  |  |  |  |  |
| Q1 | Ref. |  | Ref. |  | Ref. |  |
| Q2 | 1.52 (1.26~1.83) | <0.001 | 1.35 (1.11~1.63) | 0.002 | 1.26 (1.04~1.52) | 0.018 |
| Q3 | 1.98 (1.65~2.36) | <0.001 | 1.56 (1.28~1.88) | <0.001 | 1.35 (1.11~1.64) | 0.002 |
| Q4 | 3.54 (3.00~4.17) | <0.001 | 2.36 (1.95~2.85) | <0.001 | 1.88 (1.55~2.28) | <0.001 |
| **TyG-ABSI** | | | | | | |
| **ACD** |  |  |  |  |  |  |
| Q1 | Ref. |  | Ref. |  | Ref. |  |
| Q2 | 1.43 (1.32~1.55) | <0.001 | 1.22 (1.13~1.32) | <0.001 | 1.22 (1.12~1.32) | <0.001 |
| Q3 | 1.77 (1.64~1.92) | <0.001 | 1.39 (1.28~1.51) | <0.001 | 1.38 (1.27~1.50) | <0.001 |
| Q4 | 2.33 (2.16~2.51) | <0.001 | 1.71 (1.58~1.86) | <0.001 | 1.67 (1.53~1.82) | <0.001 |
| **AD** |  |  |  |  |  |  |
| Q1 | Ref. |  | Ref. |  | Ref. |  |
| Q2 | 1.43 (1.28~1.61) | <0.001 | 1.25 (1.11~1.40) | <0.001 | 1.27 (1.13~1.43) | <0.001 |
| Q3 | 1.65 (1.48~1.85) | <0.001 | 1.35 (1.20~1.52) | <0.001 | 1.40 (1.24~1.58) | <0.001 |
| Q4 | 1.95 (1.75~2.18) | <0.001 | 1.54 (1.36~1.74) | <0.001 | 1.59 (1.39~1.81) | <0.001 |
| **VD** |  |  |  |  |  |  |
| Q1 | Ref. |  | Ref. |  | Ref. |  |
| Q2 | 1.51 (1.25~1.82) | <0.001 | 1.22 (1.01~1.47) | 0.043 | 1.12 (0.92~1.35) | 0.256 |
| Q3 | 2.15 (1.80~2.56) | <0.001 | 1.51 (1.26~1.82) | <0.001 | 1.31 (1.08~1.59) | 0.005 |
| Q4 | 3.34 (2.83~3.94) | <0.001 | 2.14 (1.79~2.57) | <0.001 | 1.71 (1.41~2.08) | <0.001 |
| Abbreviations: TyG: Triglyceride glucose; ABSI: A body shape index; TyG-ABSI: Triglyceride Glucose-A Body Shape Index; HR (95% CI): Hazard Ratio (95% Confidence Interval); TDI: Townsend Deprivation Index; CRP: C-Reactive Protein; SBP: Systolic Blood Pressure; HDL-C: High-Density Lipoprotein Cholesterol.  Model1: Crude.  Model2: Adjusted for age, sex, race, and residence.  Model3: Age, sex, race, residence, educational level level, smoking status and drinking status, TDI, history of anxiety, history of stroke, family history of diabetes, CRP, SBP, and HDL-C were adjusted for. | | | | | | |

**Table S30** Sensitivity analysis for Cox regression analyses of TyG,ABSI and TyG-ABSI indices with dementia and subtypes excluding participants who developed dementia within 10 years after the follow-up period.

| **Types** | **Q1** | **Q2** | | **Q3** | | **Q4** | |
| --- | --- | --- | --- | --- | --- | --- | --- |
|  |  | **HR (95%CI)** | **P value** | **HR (95%CI)** | **P value** | **HR (95%CI)** | **P value** |
| **TyG** |  |  |  |  |  |  |  |
| ACD | Ref | 1.13(1.03,1.25) | 0.011 | 1.15(1.04,1.26) | 0.006 | 1.28(1.16,1.42) | <0.001 |
| AD | Ref | 1.16(1.01,1.33) | 0.033 | 1.20(1.04,1.38) | 0.012 | 1.31(1.13,1.52) | <0.001 |
| VD | Ref | 0.86(0.69,1.06) | 0.162 | 1.00(0.81,1.24) | 0.967 | 1.35(1.09,1.66) | 0.006 |
| **ABSI** |  |  |  |  |  |  |  |
| ACD | Ref | 1.17(1.05,1.29) | 0.003 | 1.31(1.18,1.46) | <0.001 | 1.66(1.49,1.85) | <0.001 |
| AD | Ref | 1.18(1.02,1.36) | 0.023 | 1.25(1.08,1.45) | 0.003 | 1.58(1.36,1.84) | <0.001 |
| VD | Ref | 1.21(0.95,1.54) | 0.129 | 1.31(1.03,1.68) | 0.031 | 1.74(1.36,2.23) | <0.001 |
| **TyG-ABSI** |  |  |  |  |  |  |  |
| ACD | Ref | 1.12(1.02,1.23) | 0.020 | 1.35(1.24,1.47) | <0.001 | 1.54(1.39,1.71) | <0.001 |
| AD | Ref | 1.15(1.00,1.32) | 0.043 | 1.34(1.18,1.53) | <0.001 | 1.48(1.26,1.73) | <0.001 |
| VD | Ref | 1.26(1.03,1.55) | 0.021 | 1.28(1.06,1.55) | 0.011 | 1.96(1.60,2.41) | <0.001 |
| Abbreviations: TyG: Triglyceride glucose; ABSI: A body shape index; TyG-ABSI: Triglyceride Glucose-A Body Shape Index; HR (95% CI): Hazard Ratio (95% Confidence Interval); TDI: Townsend Deprivation Index; CRP: C-Reactive Protein; SBP: Systolic Blood Pressure; HDL-C: High-Density Lipoprotein Cholesterol.  Model1: Crude.  Model2: Adjusted for age, sex, race, and residence.  Model3: Age, sex, race, residence, educational level, smoking status and drinking status, TDI, history of anxiety, history of stroke, family history of diabetes, SBP , CRP, and HDL were adjusted for. | | | | | | | |

**Table S31** Sensitivity analysis for Cox regression analyses of TyG,ABSI and TyG-ABSI indices with dementia and subtypes post-imputation.

| Classify | Model1 | | Model2 | | Model3 | |
| --- | --- | --- | --- | --- | --- | --- |
|  | HR (95%CI) | *P* | HR (95%CI) | *P* | HR (95%CI) | *P* |
| **TyG** | | | | | | |
| **ACD** |  |  |  |  |  |  |
| Q1 | Ref. |  | Ref. |  | Ref. |  |
| Q2 | 1.22 (1.14~1.30) | <0.001 | 1.04 (0.98~1.11) | 0.218 | 1.05 (0.98~1.12) | 0.154 |
| Q3 | 1.32 (1.24~1.41) | <0.001 | 1.07 (1.00~1.14) | 0.039 | 1.07 (1.00~1.15) | 0.036 |
| Q4 | 1.48 (1.39~1.58) | <0.001 | 1.19 (1.12~1.27) | <0.001 | 1.17 (1.09~1.26) | <0.001 |
| **AD** |  |  |  |  |  |  |
| Q1 | Ref. |  | Ref. |  | Ref. |  |
| Q2 | 1.25 (1.13~1.37) | <0.001 | 1.07 (0.97~1.17) | 0.200 | 1.08 (0.98~1.19) | 0.128 |
| Q3 | 1.34 (1.22~1.47) | <0.001 | 1.09 (0.99~1.20) | 0.087 | 1.10 (1.00~1.22) | 0.053 |
| Q4 | 1.37(1.24~1.50) | <0.001 | 1.13 (1.02~1.24) | 0.015 | 1.14 (1.02~1.26) | 0.016 |
| **VD** |  |  |  |  |  |  |
| Q1 | Ref. |  | Ref. |  | Ref. |  |
| Q2 | 1.21 (1.04~1.40) | 0.011 | 1.01 (0.87~1.16) | 0.937 | 0.98 (0.84~1.14) | 0.801 |
| Q3 | 1.44 (1.25~1.65) | <0.001 | 1.11 (0.97~1.28) | 0.131 | 1.05 (0.91~1.22) | 0.483 |
| Q4 | 1.98 (1.74~2.27) | <0.001 | 1.50 (1.31~1.72) | <0.001 | 1.32 (1.14~1.53) | <0.001 |
| **ABSI** | | | | | | |
| **ACD** |  |  |  |  |  |  |
| Q1 | Ref. |  | Ref. |  | Ref. |  |
| Q2 | 1.30 (1.21~1.40) | <0.001 | 1.22 (1.14~1.31) | <0.001 | 1.20 (1.12~1.30) | <0.001 |
| Q3 | 1.70 (1.59~1.82) | <0.001 | 1.50 (1.40~1.62) | <0.001 | 1.44 (1.34~1.55) | <0.001 |
| Q4 | 2.53 (2.38~2.70) | <0.001 | 1.99 (1.84~2.14) | <0.001 | 1.82 (1.69~1.96) | <0.001 |
| **AD** |  |  |  |  |  |  |
| Q1 | Ref. |  | Ref. |  | Ref. |  |
| Q2 | 1.23 (1.11~1.36) | <0.001 | 1.17 (1.06~1.30) | 0.003 | 1.17 (1.06~1.30) | 0.003 |
| Q3 | 1.47 (1.33~1.62) | <0.001 | 1.36 (1.22~1.51) | <0.001 | 1.34 (1.20~1.49) | <0.001 |
| Q4 | 2.02 (1.84~2.22) | <0.001 | 1.68 (1.51~1.87) | <0.001 | 1.61 (1.44~1.80) | <0.001 |
| **VD** |  |  |  |  |  |  |
| Q1 | Ref. |  | Ref. |  | Ref. |  |
| Q2 | 1.57 (1.33~1.85) | <0.001 | 1.38 (1.17~1.63) | <0.001 | 1.29 (1.09~1.53) | 0.003 |
| Q3 | 2.10 (1.79~2.46) | <0.001 | 1.63 (1.38~1.93) | <0.001 | 1.42 (1.20~1.68) | <0.001 |
| Q4 | 3.66 (3.16~4.24) | <0.001 | 2.40 (2.03~2.84) | <0.001 | 1.91 (1.61~2.27) | <0.001 |
| **TyG-ABSI** | | | | | | |
| **ACD** |  |  |  |  |  |  |
| Q1 | Ref. |  | Ref. |  | Ref. |  |
| Q2 | 1.45 (1.35~1.55) | <0.001 | 1.23 (1.15~1.32) | <0.001 | 1.22 (1.14~1.32) | <0.001 |
| Q3 | 1.79 (1.67~1.92) | <0.001 | 1.39 (1.29~1.50) | <0.001 | 1.39 (1.29~1.49) | <0.001 |
| Q4 | 2.36 (2.21~2.52) | <0.001 | 1.73 (1.61~1.86) | <0.001 | 1.69 (1.56~1.82) | <0.001 |
| **AD** |  |  |  |  |  |  |
| Q1 | Ref. |  | Ref. |  | Ref. |  |
| Q2 | 1.42 (1.28~1.57) | <0.001 | 1.23 (1.11~1.37) | <0.001 | 1.25 (1.12~1.39) | <0.001 |
| Q3 | 1.66 (1.50~1.83) | <0.001 | 1.34 (1.21~1.49) | <0.001 | 1.37 (1.23~1.53) | <0.001 |
| Q4 | 1.95 (1.77~2.15) | <0.001 | 1.52 (1.37~1.69) | <0.001 | 1.55 (1.38~1.74) | <0.001 |
| **VD** |  |  |  |  |  |  |
| Q1 | Ref. |  | Ref. |  | Ref. |  |
| Q2 | 1.69 (1.43~1.99) | <0.001 | 1.36 (1.15~1.60) | 0.005 | 1.25 (1.05~1.48) | 0.010 |
| Q3 | 2.27 (1.94~2.66) | <0.001 | 1.58 (1.34~1.87) | <0.001 | 1.40 (1.18~1.66) | <0.001 |
| Q4 | 3.59 (3.09~4.17) | <0.001 | 2.28 (1.94~2.69) | <0.001 | 1.85 (1.56~2.21) | <0.001 |
| Abbreviations: TyG: Triglyceride glucose; ABSI: A body shape index; TyG-ABSI: Triglyceride Glucose-A Body Shape Index; HR (95% CI): Hazard Ratio (95% Confidence Interval); TDI: Townsend Deprivation Index; CRP: C-Reactive Protein; SBP: Systolic Blood Pressure; HDL-C: High-Density Lipoprotein Cholesterol.  Model1: Crude.  Model2: Adjusted for age, sex, race, and residence.  Model3: Age, sex, race, residence, educational level level, smoking status and drinking status, TDI, history of anxiety, history of stroke, family history of diabetes, CRP, SBP, and HDL-C were adjusted for. | | | | | | |

**Table S32** Sensitivity analysis for performing Cox regression analyses of TyG,ABSI and TyG-ABSI indices with dementia and subtypes after removing outliers.

| Classify | Model1 | | Model2 | | Model3 | |
| --- | --- | --- | --- | --- | --- | --- |
|  | HR (95%CI) | *P* | HR (95%CI) | *P* | HR (95%CI) | *P* |
| **TyG** | | | | | | |
| **ACD** |  |  |  |  |  |  |
| Q1 | Ref. |  | Ref. |  | Ref. |  |
| Q2 | 1.27 (1.18~1.37) | <0.001 | 1.08 (1.00~1.16) | 0.057 | 1.08 (1.00~1.17) | 0.053 |
| Q3 | 1.37 (1.27~1.47) | <0.001 | 1.09 (1.01~1.17) | 0.033 | 1.09 (1.01~1.18) | 0.033 |
| Q4 | 1.48 (1.37~1.59) | <0.001 | 1.16 (1.08~1.25) | <0.001 | 1.15 (1.06~1.25) | 0.001 |
| **AD** |  |  |  |  |  |  |
| Q1 | Ref. |  | Ref. |  | Ref. |  |
| Q2 | 1.28 (1.14~1.43) | <0.001 | 1.08 (0.97~1.21) | 0.169 | 1.10 (0.99~1.24) | 0.088 |
| Q3 | 1.40 (1.25~1.56) | <0.001 | 1.12 (1.00~1.25) | 0.048 | 1.16 (1.03~1.30) | 0.013 |
| Q4 | 1.36(1.22~1.52) | <0.001 | 1.10 (0.98~1.23) | 0.108 | 1.15 (1.01~1.30) | 0.033 |
| **VD** |  |  |  |  |  |  |
| Q1 | Ref. |  | Ref. |  | Ref. |  |
| Q2 | 1.21 (1.02~1.44) | 0.031 | 1.00 (0.84~1.19) | 0.972 | 0.97 (0.81~1.15) | 0.694 |
| Q3 | 1.47 (1.24~1.73) | <0.001 | 1.12 (0.94~1.32) | 0.199 | 1.06 (0.89~1.26) | 0.529 |
| Q4 | 1.91 (1.63~2.24) | <0.001 | 1.41 (1.20~1.66) | <0.001 | 1.26 (1.06~1.51) | 0.010 |
| **ABSI** | | | | | | |
| **ACD** |  |  |  |  |  |  |
| Q1 | Ref. |  | Ref. |  | Ref. |  |
| Q2 | 1.33 (1.22~1.45) | <0.001 | 1.24 (1.14~1.35) | <0.001 | 1.23 (1.13~1.34) | <0.001 |
| Q3 | 1.68 (1.54~1.82) | <0.001 | 1.47 (1.35~1.60) | <0.001 | 1.42 (1.30~1.56) | <0.001 |
| Q4 | 2.55 (2.36~2.75) | <0.001 | 1.96 (1.79~2.14) | <0.001 | 1.84 (1.68~2.01) | <0.001 |
| **AD** |  |  |  |  |  |  |
| Q1 | Ref. |  | Ref. |  | Ref. |  |
| Q2 | 1.28 (1.14~1.44) | <0.001 | 1.22 (1.08~1.38) | 0.001 | 1.23 (1.09~1.40) | <0.001 |
| Q3 | 1.43 (1.27~1.61) | <0.001 | 1.32 (1.16~1.50) | <0.001 | 1.32 (1.16~1.50) | <0.001 |
| Q4 | 2.05 (1.83~2.29) | <0.001 | 1.68 (1.48~1.91) | <0.001 | 1.66 (1.46~1.90) | <0.001 |
| **VD** |  |  |  |  |  |  |
| Q1 | Ref. |  | Ref. |  | Ref. |  |
| Q2 | 1.61 (1.32~1.96) | <0.001 | 1.42 (1.16~1.74) | <0.001 | 1.34 (1.09~1.65) | 0.005 |
| Q3 | 2.16 (1.79~2.62) | <0.001 | 1.68 (1.37~2.07) | <0.001 | 1.49 (1.21~1.84) | <0.001 |
| Q4 | 3.79 (3.17~4.53) | <0.001 | 2.46 (2.01~3.02) | <0.001 | 2.04 (1.66~2.51) | <0.001 |
| **TyG-ABSI** | | | | | | |
| **ACD** |  |  |  |  |  |  |
| Q1 | Ref. |  | Ref. |  | Ref. |  |
| Q2 | 1.51 (1.39~1.64) | <0.001 | 1.27 (1.17~1.38) | <0.001 | 1.27 (1.17~1.38) | <0.001 |
| Q3 | 1.82 (1.68~1.97) | <0.001 | 1.39 (1.28~1.52) | <0.001 | 1.40 (1.28~1.53) | <0.001 |
| Q4 | 2.37 (2.19~2.56) | <0.001 | 1.68 (1.54~1.83) | <0.001 | 1.67 (1.52~1.84) | <0.001 |
| **AD** |  |  |  |  |  |  |
| Q1 | Ref. |  | Ref. |  | Ref. |  |
| Q2 | 1.51 (1.34~1.70) | <0.001 | 1.30 (1.15~1.46) | <0.001 | 1.33 (1.18~1.50) | <0.001 |
| Q3 | 1.73 (1.54~1.94) | <0.001 | 1.39 (1.22~1.57) | <0.001 | 1.46 (1.28~1.66) | <0.001 |
| Q4 | 1.97 (1.76~2.21) | <0.001 | 1.50 (1.32~1.71) | <0.001 | 1.60 (1.39~1.84) | <0.001 |
| **VD** |  |  |  |  |  |  |
| Q1 | Ref. |  | Ref. |  | Ref. |  |
| Q2 | 1.67 (1.37~2.03) | <0.001 | 1.32 (1.08~1.61) | 0.007 | 1.23 (1.01~1.51) | 0.042 |
| Q3 | 2.30 (1.91~2.78) | <0.001 | 1.58 (1.30~1.92) | <0.001 | 1.43 (1.16~1.75) | <0.001 |
| Q4 | 3.48 (2.92~4.16) | <0.001 | 2.14 (1.76~2.60) | <0.001 | 1.82 (1.47~2.24) | <0.001 |
| Abbreviations: TyG: Triglyceride glucose; ABSI: A body shape index; TyG-ABSI: Triglyceride Glucose-A Body Shape Index; HR (95% CI): Hazard Ratio (95% Confidence Interval); TDI: Townsend Deprivation Index; CRP: C-Reactive Protein; SBP: Systolic Blood Pressure; HDL-C: High-Density Lipoprotein Cholesterol.  Model1: Crude.  Model2: Adjusted for age, sex, race, and residence.  Model3: Age, sex, race, residence, educational level level, smoking status and drinking status, TDI, history of anxiety, history of stroke, family history of diabetes, CRP, SBP, and HDL-C were adjusted for. | | | | | | |

**Table S33** Sensitivity analysis for performing Cox regression analyses of TyG, ABSI and TyG-ABSI indices with dementia and subtypes after adding sleep duration to the full adjustment model.

| Classify | Model1 | | Model2 | | Model3 | |
| --- | --- | --- | --- | --- | --- | --- |
|  | HR (95%CI) | *P* | HR (95%CI) | *P* | HR (95%CI) | *P* |
| **TyG** | | | | | | |
| **ACD** |  |  |  |  |  |  |
| Q1 | Ref. |  | Ref. |  | Ref. |  |
| Q2 | 1.24 (1.15~1.33) | <0.001 | 1.06 (0.98~1.13) | 0.141 | 1.06 (0.98~1.14) | 0.135 |
| Q3 | 1.33 (1.24~1.43) | <0.001 | 1.08 (1.00~1.16) | 0.040 | 1.08 (1.00~1.16) | 0.040 |
| Q4 | 1.48 (1.38~1.59) | <0.001 | 1.19 (1.11~1.27) | <0.001 | 1.17 (1.08~1.26) | <0.001 |
| **AD** |  |  |  |  |  |  |
| Q1 | Ref. |  | Ref. |  | Ref. |  |
| Q2 | 1.24 (1.11~1.38) | <0.001 | 1.06 (0.95~1.17) | 0.308 | 1.07 (0.96~1.19) | 0.212 |
| Q3 | 1.35 (1.22~1.50) | <0.001 | 1.10 (0.99~1.22) | 0.070 | 1.13 (1.01~1.26) | 0.030 |
| Q4 | 1.35(1.22~1.50) | <0.001 | 1.11 (1.00~1.24) | 0.049 | 1.13 (1.01~1.27) | 0.034 |
| **VD** |  |  |  |  |  |  |
| Q1 | Ref. |  | Ref. |  | Ref. |  |
| Q2 | 1.18 (1.00~1.38) | 0.045 | 0.98 (0.83~1.15) | 0.781 | 0.95 (0.80~1.12) | 0.519 |
| Q3 | 1.43 (1.23~1.67) | <0.001 | 1.10 (0.95~1.29) | 0.209 | 1.05 (0.89~1.23) | 0.553 |
| Q4 | 1.98 (1.72~2.29) | <0.001 | 1.49 (1.29~1.73) | <0.001 | 1.32 (1.12~1.55) | <0.001 |
| **ABSI** | | | | | | |
| **ACD** |  |  |  |  |  |  |
| Q1 | Ref. |  | Ref. |  | Ref. |  |
| Q2 | 1.31 (1.21~1.42) | <0.001 | 1.23 (1.14~1.33) | <0.001 | 1.22 (1.12~1.32) | <0.001 |
| Q3 | 1.69 (1.56~1.82) | <0.001 | 1.49 (1.37~1.61) | <0.001 | 1.43 (1.31~1.55) | <0.001 |
| Q4 | 2.52 (2.35~2.70) | <0.001 | 1.96 (1.81~2.13) | <0.001 | 1.79 (1.65~1.95) | <0.001 |
| **AD** |  |  |  |  |  |  |
| Q1 | Ref. |  | Ref. |  | Ref. |  |
| Q2 | 1.24 (1.11~1.39) | <0.001 | 1.19 (1.06~1.33) | 0.003 | 1.19 (1.06~1.34) | 0.003 |
| Q3 | 1.44 (1.29~1.61) | <0.001 | 1.33 (1.19~1.50) | <0.001 | 1.32 (1.17~1.49) | <0.001 |
| Q4 | 1.98 (1.79~2.20) | <0.001 | 1.64 (1.46~1.85) | <0.001 | 1.58 (1.40~1.79) | <0.001 |
| **VD** |  |  |  |  |  |  |
| Q1 | Ref. |  | Ref. |  | Ref. |  |
| Q2 | 1.59 (1.33~1.92) | <0.001 | 1.41 (1.17~1.70) | <0.001 | 1.33 (1.10~1.60) | 0.003 |
| Q3 | 2.12 (1.78~2.53) | <0.001 | 1.65 (1.37~1.99) | <0.001 | 1.44 (1.20~1.75) | <0.001 |
| Q4 | 3.79 (3.22~4.46) | <0.001 | 2.48 (2.06~2.99) | <0.001 | 1.99 (1.64~2.40) | <0.001 |
| **TyG-ABSI** | | | | | | |
| **ACD** |  |  |  |  |  |  |
| Q1 | Ref. |  | Ref. |  | Ref. |  |
| Q2 | 1.46 (1.35~1.58) | <0.001 | 1.24 (1.15~1.35) | <0.001 | 1.24 (1.14~1.34) | <0.001 |
| Q3 | 1.81 (1.68~1.96) | <0.001 | 1.41 (1.30~1.53) | <0.001 | 1.40 (1.29~1.52) | <0.001 |
| Q4 | 2.34 (2.20~2.54) | <0.001 | 1.72 (1.59~1.86) | <0.001 | 1.67 (1.53~1.82) | <0.001 |
| **AD** |  |  |  |  |  |  |
| Q1 | Ref. |  | Ref. |  | Ref. |  |
| Q2 | 1.44 (1.29~1.61) | <0.001 | 1.25 (1.11~1.40) | <0.001 | 1.27 (1.13~1.43) | <0.001 |
| Q3 | 1.69 (1.51~1.88) | <0.001 | 1.37 (1.22~1.54) | <0.001 | 1.42 (1.25~1.60) | <0.001 |
| Q4 | 1.93 (1.73~2.15) | <0.001 | 1.50 (1.34~1.69) | <0.001 | 1.55 (1.36~1.76) | <0.001 |
| **VD** |  |  |  |  |  |  |
| Q1 | Ref. |  | Ref. |  | Ref. |  |
| Q2 | 1.63 (1.36~1.96) | <0.001 | 1.30 (1.08~1.57) | 0.005 | 1.21 (1.00~1.46) | 0.047 |
| Q3 | 2.32 (1.95~2.76) | <0.001 | 1.62 (1.35~1.94) | <0.001 | 1.43 (1.19~1.73) | <0.001 |
| Q4 | 3.59 (3.05~4.23) | <0.001 | 2.26 (1.89~2.71) | <0.001 | 1.86 (1.54~2.26) | <0.001 |
| Abbreviations: TyG: Triglyceride glucose; ABSI: A body shape index; TyG-ABSI: Triglyceride Glucose-A Body Shape Index; HR (95% CI): Hazard Ratio (95% Confidence Interval); TDI: Townsend Deprivation Index; CRP: C-Reactive Protein; SBP: Systolic Blood Pressure; HDL-C: High-Density Lipoprotein Cholesterol.  Model1: Crude.  Model2: Adjusted for age, sex, race, and residence.  Model3: Age, sex, race, residence, educational level level, smoking status and drinking status, TDI, history of anxiety, history of stroke, family history of diabetes, CRP, SBP, and HDL-C were adjusted for. | | | | | | |

**Table S34** Sensitivity analysis was performed using a Fine-Gray model to evaluate the robustness of the association between indices and dementia, with death treated as a competing event in the model.

| Classify | Model1 | | | Model2 | | | | Model3 | | | |  |
| --- | --- | --- | --- | --- | --- | --- | --- | --- | --- | --- | --- | --- |
|  | HR (95%CI) | | *P* | HR (95%CI) | | *P* | | HR (95%CI) | | *P* | |  |
| **TyG** | | | | | | | | | | | |  |
| **ACD** |  | |  |  | |  | |  | |  | |  |
| Q1 | Ref. | |  | Ref. | |  | | Ref. | |  | |  |
| Q2 | 1.23 (1.14~1.32) | | <0.001 | 1.06 (0.98~1.14) | | 0.130 | | 1.06 (0.99~1.14) | | 0.093 | |  |
| Q3 | 1.31 (1.22~1.40) | | <0.001 | 1.07 (1.00~1.15) | | 0.590 | | 1.9(1.01~1.17) | | 0.030 | |  |
| Q4 | 1.44 (1.35~1.54) | | <0.001 | 1.17 (1.09~1.26) | | <0.001 | | 1.17 (1.08~1.26) | | <0.001 | |  |
| **AD** |  | |  |  | |  | |  | |  | |  |
| Q1 | Ref. | |  | Ref. | |  | | Ref. | |  | |  |
| Q2 | 1.23 (1.10~1.36) | | <0.001 | 1.06 (0.95~1.18) | | 0.300 | | 1.08 (0.97~1.20) | | 0.180 | |  |
| Q3 | 1.33 (1.20~1.48) | | <0.001 | 1.10 (0.99~1.22) | | 0.082 | | 1.13 (1.02~1.27) | | 0.025 | |  |
| Q4 | 1.31(1.18~1.46) | | <0.001 | 1.10 (0.99~1.22) | | 0.094 | | 1.14 (1.01~1.28) | | 0.034 | |  |
| **VD** |  | |  |  | |  | |  | |  | |  |
| Q1 | Ref. | |  | Ref. | |  | | Ref. | |  | |  |
| Q2 | 1.17 (1.00~1.38) | | 0.050 | 0.98 (0.84~1.16) | | 0.850 | | 0.96 (0.81~1.13) | | 0.590 | |  |
| Q3 | 1.41 (1.21~1.64) | | <0.001 | 1.10 (0.95~1.29) | | 0.210 | | 1.05 (0.90~1.24) | | 0.520 | |  |
| Q4 | 1.94 (1.68~2.24) | | <0.001 | 1.48 (1.28~1.72) | | <0.001 | | 1.32(1.13~1.55) | | <0.001 | |  |
| **ABSI** | | | | | | | | | | | |  |
| **ACD** |  | |  |  | |  | |  | |  | |  |
| Q1 | Ref. | |  | Ref. | |  | | Ref. | |  | |  |
| Q2 | 1.29 (1.20~1.40) | | <0.001 | 1.22 (1.12~1.32) | | <0.001 | | 1.21 (1.12~1.31) | | <0.001 | |  |
| Q3 | 1.64 (1.52~1.77) | | <0.001 | 1.46 (1.35~1.58) | | <0.001 | | 1.41 (1.30~1.53) | | <0.001 | |  |
| Q4 | 2.37 (2.21~2.54) | | <0.001 | 1.86 (1.71~2.02) | | <0.001 | | 1.72 (1.58~1.87) | | <0.001 | |  |
| **AD** |  | |  |  | |  | |  | |  | |  |
| Q1 | Ref. | |  | Ref. | |  | | Ref. | |  | |  |
| Q2 | 1.24 (1.11~1.38) | | <0.001 | 1.18 (1.06~1.33) | | 0.004 | | 1.19 (1.06~1.33) | | 0.003 | |  |
| Q3 | 1.43 (1.28~1.60) | | <0.001 | 1.32 (1.18~1.49) | | <0.001 | | 1.31 (1.16~1.47) | | <0.001 | |  |
| Q4 | 1.97 (1.78~2.18) | | <0.001 | 1.63 (1.45~1.84) | | <0.001 | | 1.52 (1.34~1.72) | | <0.001 | |  |
| **VD** |  | |  |  | |  | |  | |  | |  |
| Q1 | Ref. | |  | Ref. | |  | | Ref. | |  | |  |
| Q2 | 1.58 (1.32~1.90) | | <0.001 | 1.40 (1.17~1.69) | | <0.001 | | 1.33 (1.10~1.60) | | 0.003 | |  |
| Q3 | 2.08(1.75~2.48) | | <0.001 | 1.64 (1.36~1.98) | | <0.001 | | 1.44 (1.19~1.74) | | <0.001 | |  |
| Q4 | 3.57(3.03~4.19) | | <0.001 | 2.36 (1.95~2.84) | | <0.001 | | 1.91 (1.58~2.31) | | <0.001 | |  |
| **TyG-ABSI** | | | | | | | | | | | |  |
| **ACD** | |  |  | |  | |  | |  | |  | |
| Q1 | | Ref. |  | | Ref. | |  | | Ref. | |  | |
| Q2 | | 1.44 (1.34~1.56) | <0.001 | | 1.23 (1.13~1.33) | | <0.001 | | 1.23 (1.13~1.33) | | <0.001 | |
| Q3 | | 1.80 (1.67~1.94) | <0.001 | | 1.40 (1.29~1.51) | | <0.001 | | 1.40 (1.29~1.52) | | <0.001 | |
| Q4 | | 2.34 (2.18~2.51) | <0.001 | | 1.70 (1.57~1.85) | | <0.001 | | 1.67 (1.53~1.82) | | <0.001 | |
| **AD** | |  |  | |  | |  | |  | |  | |
| Q1 | | Ref. |  | | Ref. | |  | | Ref. | |  | |
| Q2 | | 1.42 (1.27~1.59) | <0.001 | | 1.24 (1.10~1.38) | | <0.001 | | 1.26 (1.12~1.41) | | <0.001 | |
| Q3 | | 1.68 (1.50~1.87) | <0.001 | | 1.36 (1.21~1.53) | | <0.001 | | 1.40 (1.24~1.58) | | <0.001 | |
| Q4 | | 1.91 (1.72~2.13) | <0.001 | | 1.49 (1.32~1.68) | | <0.001 | | 1.50 (1.31~1.70) | | <0.001 | |
| **VD** | |  |  | |  | |  | |  | |  | |
| Q1 | | Ref. |  | | Ref. | |  | | Ref. | |  | |
| Q2 | | 1.56 (1.30~1.87) | <0.001 | | 1.26 (1.04~1.51) | | 0.002 | | 1.17 (0.97~1.41) | | 0.100 | |
| Q3 | | 2.21 (1.86~2.62) | <0.001 | | 1.56 (1.30~1.87) | | <0.001 | | 1.39 (1.15~1.68) | | <0.001 | |
| Q4 | | 3.35 (2.85~3.94) | <0.001 | | 2.14 (1.78~2.57) | | <0.001 | | 1.78 (1.47~2.15) | | <0.001 | |
| Abbreviations: TyG: Triglyceride glucose; ABSI: A body shape index; TyG-ABSI: Triglyceride Glucose-A Body Shape Index; HR (95% CI): Hazard Ratio (95% Confidence Interval); TDI: Townsend Deprivation Index; CRP: C-Reactive Protein; SBP: Systolic Blood Pressure; HDL-C: High-Density Lipoprotein Cholesterol.  Model1: Crude.  Model2: Adjusted for age, sex, race, and residence.  Model3: Age, sex, race, residence, educational level level, smoking status and drinking status, TDI, history of anxiety, history of stroke, family history of diabetes, CRP, SBP, and HDL-C were adjusted for. | | | | | | | | | | | |  |

**Table S35** Sensitivity analysis for Cox regression analyses of TyG, ABSI and TyG-ABSI indices with dementia and subtypes was performed without adjusting for HDL-C and CRP in the fully adjusted model.

| **Types** | **Q1** | **Q2** | | **Q3** | | **Q4** | |
| --- | --- | --- | --- | --- | --- | --- | --- |
|  |  | **HR (95%CI)** | **P value** | **HR (95%CI)** | **P value** | **HR (95%CI)** | **P value** |
| **TyG** |  |  |  |  |  |  |  |
| ACD | Ref | 1.04(0.97,1.12) | 0.230 | 1.05(0.98,1.12) | 0.199 | 1.12(1.04,1.20) | 0.002 |
| AD | Ref | 1.04(0.94,1.16) | 0.429 | 1.07(0.97,1.19) | 0.195 | 1.06(0.95,1.18) | 0.301 |
| VD | Ref | 0.96(0.82,1.13) | 0.648 | 1.07(0.91,1.24) | 0.424 | 1.37(1.18,1.59) | <0.001 |
| **ABSI** |  |  |  |  |  |  |  |
| ACD | Ref | 1.20(1.11,1.30) | <0.001 | 1.40(1.29,1.52) | <0.001 | 1.76(1.62,1.91) | <0.001 |
| AD | Ref | 1.17(1.04,1.31) | 0.008 | 1.28(1.14,1.44) | <0.001 | 1.52(1.35,1.72) | <0.001 |
| VD | Ref | 1.35(1.12,1.62) | 0.002 | 1.49(1.24,1.80) | <0.001 | 2.05(1.70,2.47) | <0.001 |
| **TyG-ABSI** |  |  |  |  |  |  |  |
| ACD | Ref | 1.05(0.98,1.13) | 0.160 | 1.38(1.29,1.47) | <0.001 | 1.46(1.35,1.58) | <0.001 |
| AD | Ref | 1.02(0.92,1.14) | 0.655 | 1.31(1.19,1.45) | <0.001 | 1.25(1.10,1.41) | <0.001 |
| VD | Ref | 1.24(1.07,1.44) | 0.005 | 1.40(1.22,1.61) | <0.001 | 1.95(1.68,2.26) | <0.001 |
| **Abbreviations:** TyG: Triglyceride glucose; ABSI: A body shape index; TyG-ABSI: Triglyceride Glucose-A Body Shape Index; HR (95% CI): Hazard Ratio (95% Confidence Interval); TDI: Townsend Deprivation Index; SBP: Systolic Blood Pressure.  Model1: Crude.  Model2: Adjusted for age, sex, race, and residence.  Model3: Age, sex, race, residence, educational level, smoking status and drinking status, TDI, history of anxiety, history of stroke, family history of diabetes, SBP were adjusted for. | | | | | | | |

**Table S36** Sensitivity analysis for Cox regression analyses of TyG, ABSI and TyG-ABSI indices with dementia and subtypes was performed additionally adjusting for glycated haemoglobin (HbA1c) in the fully adjusted model.

| **Types** | **Q1** | **Q2** | | **Q3** | | **Q4** | |
| --- | --- | --- | --- | --- | --- | --- | --- |
|  |  | **HR (95%CI)** | **P value** | **HR (95%CI)** | **P value** | **HR (95%CI)** | **P value** |
| **TyG** |  |  |  |  |  |  |  |
| ACD | Ref | 1.05(0.98,1.13) | 0.174 | 1.07(0.99,1.15) | 0.076 | 1.13(1.04,1.22) | 0.003 |
| AD | Ref | 1.08(0.97,1.21) | 0.165 | 1.13(1.01,1.26) | 0.035 | 1.10(0.98,1.24) | 0.110 |
| VD | Ref | 0.96(0.81,1.14) | 0.640 | 1.06(0.90,1.26) | 0.455 | 1.29(1.10,1.52) | 0.002 |
| **ABSI** |  |  |  |  |  |  |  |
| ACD | Ref | 1.20(1.11,1.31) | <0.001 | 1.41(1.29,1.53) | <0.001 | 1.75(1.61,1.91) | <0.001 |
| AD | Ref | 1.16(1.03,1.31) | 0.011 | 1.28(1.13,1.45) | <0.001 | 1.52(1.34,1.72) | <0.001 |
| VD | Ref | 1.32(1.09,1.60) | 0.004 | 1.44(1.19,1.75) | <0.001 | 1.96(1.61,2.37) | <0.001 |
| **TyG-ABSI** |  |  |  |  |  |  |  |
| ACD | Ref | 1.05(0.98,1.13) | 0.071 | 1.37(1.28,1.47) | <0.001 | 1.44(1.33,1.57) | <0.001 |
| AD | Ref | 1.05(0.94,1.17) | 0.655 | 1.32(1.20,1.47) | <0.001 | 1.24(1.09,1.41) | <0.001 |
| VD | Ref | 1.16(0.99,1.35) | 0.071 | 1.36(1.18,1.57) | <0.001 | 1.82(1.56,2.13) | <0.001 |
| Abbreviations: TyG: Triglyceride glucose; ABSI: A body shape index; TyG-ABSI: Triglyceride Glucose-A Body Shape Index; HR (95% CI): Hazard Ratio (95% Confidence Interval); TDI: Townsend Deprivation Index; CRP: C-Reactive Protein; SBP: Systolic Blood Pressure; HDL-C: High-Density Lipoprotein Cholesterol; HbA1c: glycated haemoglobin.  Model1: Crude.  Model2: Adjusted for age, sex, race, and residence.  Model3: Age, sex, race, residence, educational level, smoking status and drinking status, TDI, history of anxiety, history of stroke, family history of diabetes, SBP , CRP, HDL, and **HbA1c** were adjusted for. | | | | | | | |


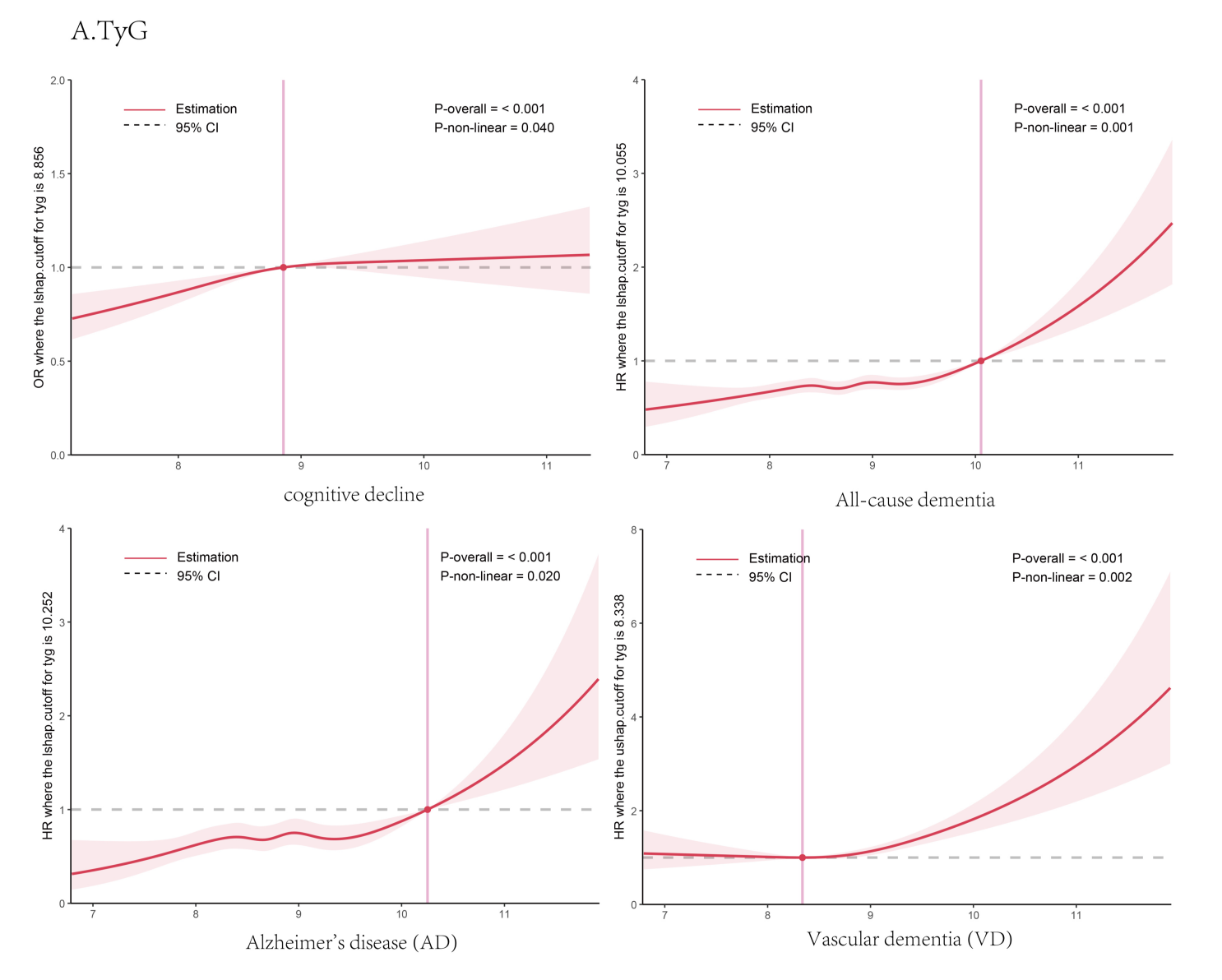


**Fig. S1** RCS curves for TyG index with cognitive function, all-cause dementia, Alzheimer's dementia and vascular dementia.


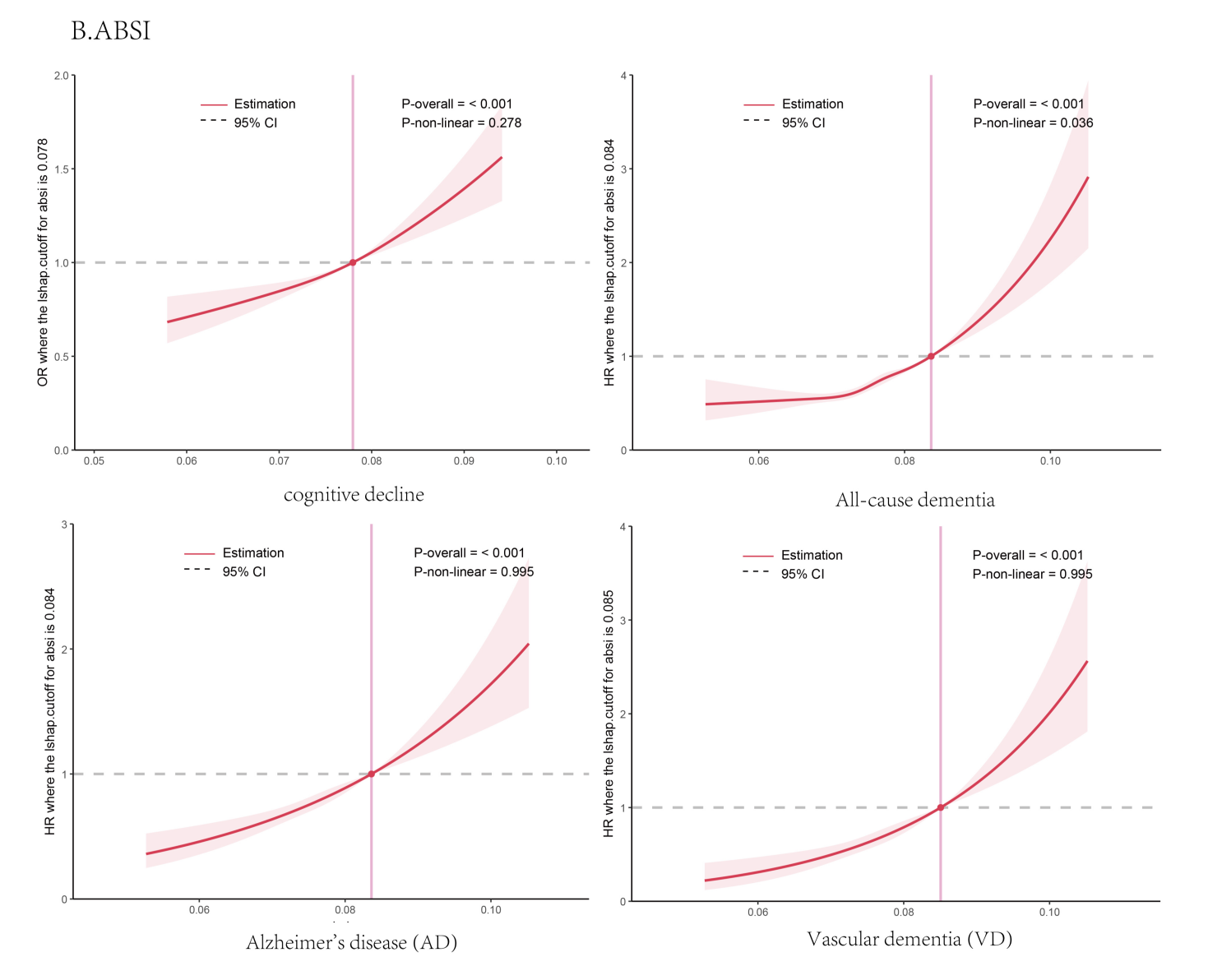


**Fig. S2** RCS curves for ABSI with cognitive function, all-cause dementia, Alzheimer's dementia and vascular dementia.


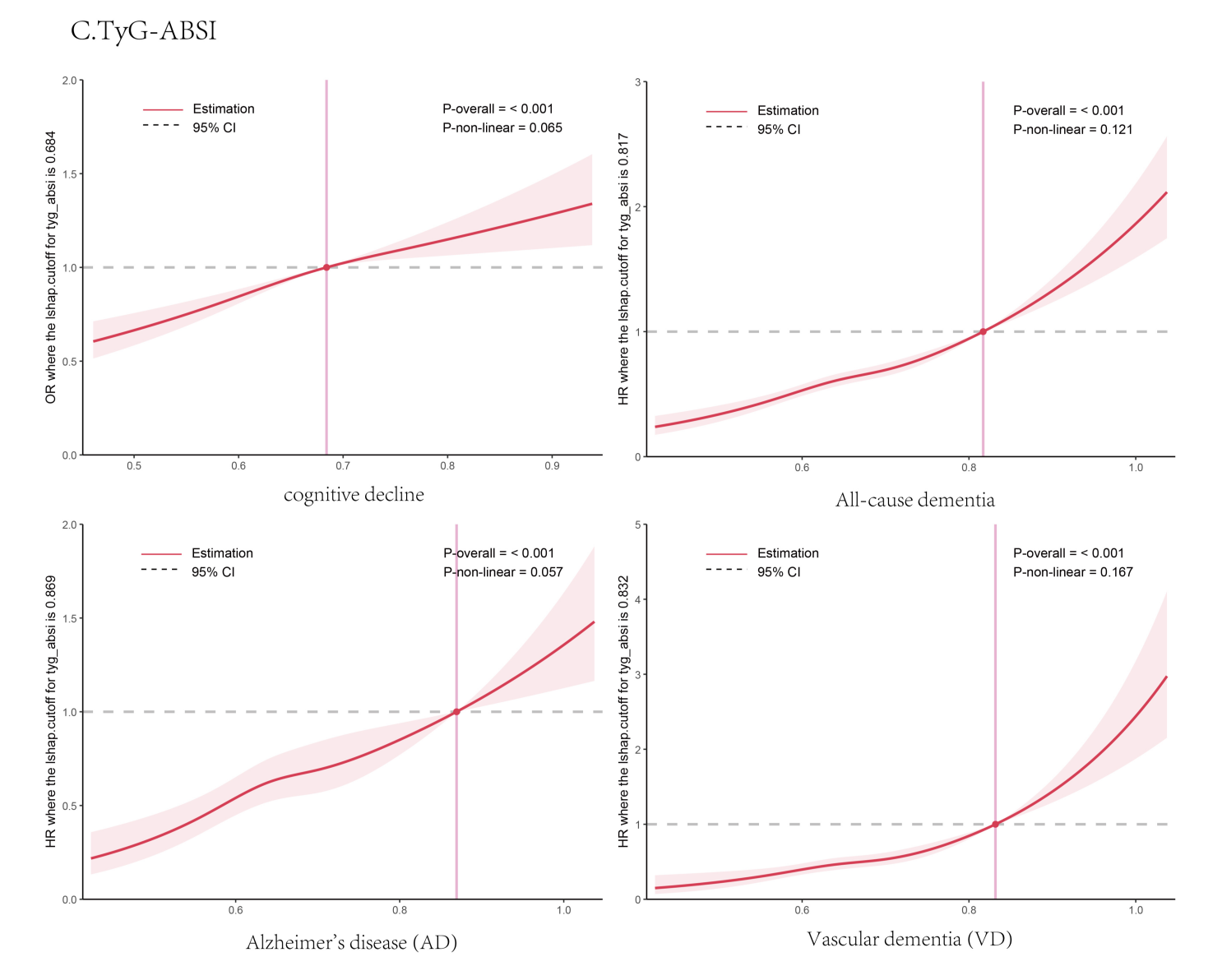


**Fig. S3** RCS curves for TyG-ABSI index with cognitive function, all-cause dementia, Alzheimer's dementia and vascular dementia.
